# Furofuranoid-Type Lignans and Related Phenolics from *Anisacanthus virgularis* (Salisb.) Nees with Promising Anticholinesterase and Anti-Ageing Properties: A Study Supported by Molecular Modelling

**DOI:** 10.3390/plants13020150

**Published:** 2024-01-05

**Authors:** Mohamed A. A. Orabi, Reda A. Abdelhamid, Hanan Elimam, Yaseen A. M. M. Elshaier, Ahmed A. Ali, Nayef Aldabaan, Abdulaziz Hassan Alhasaniah, Mohamed S. Refaey

**Affiliations:** 1 Department of Pharmacognosy, College of Pharmacy, Najran University, Najran 66454, Saudi Arabia; 2 Department of Pharmacognosy, Faculty of Pharmacy, Al-Azhar University, Assiut-Branch, Assiut 71524, Egypt; reda.ahmed@azhar.edu.eg; 3 Department of Biochemistry, Faculty of Pharmacy, University of Sadat City, Sadat City 32958, Egypt; hanan.elimam@fop.usc.edu.eg; 4 Department of Organic and Medicinal Chemistry, Faculty of Pharmacy, University of Sadat City, Sadat City 32958, Egypt; yaseen.elshaier@fop.usc.edu.eg; 5 Department of Pharmacognosy, Faculty of Pharmacy, Assiut University, Assiut 71526, Egypt; ahmed.ibrahim4@pharm.aun.edu.eg; 6 Department of Pharmacology, College of Pharmacy, Najran University, Najran 66454, Saudi Arabia; naaldabaan@nu.edu.sa; 7 Department of Clinical Laboratory Sciences, College of Applied Medical Sciences, Najran University, Najran 66454, Saudi Arabia; ahalhasaniah@nu.edu.sa; 8 Department of Pharmacognosy, Faculty of Pharmacy, University of Sadat City, Sadat City 32958, Egypt

**Keywords:** *Anisacanthus virgularis*, furofuranoid lignans, anticholinesterase, telomerase, Alzheimer’s disease, molecular docking

## Abstract

Lignan phytomolecules demonstrate promising anti-Alzheimer activity by alleviating dementia and preserving nerve cells. The purpose of this work is to characterize the lignans of *Anisacanthus virgularis* and explore their potential anti-acetylcholinesterase and anti-ageing effects. Phytochemical investigation of *A. virgularis* aerial parts afforded a new furofuranoid-type lignan (**1**), four known structural analogues, namely pinoresinol (**2**), epipinoresinol (**3**), phillyrin (**4**), and pinoresinol 4-*O*-*β*-d-glucoside (**5**), in addition to *p*-methoxy-*trans*-methyl cinnamate (**6**) and 1H-indole-3-carboxaldehyde (**7**). The structures were established from thorough spectroscopic analyses and comparisons with the literature. Assessment of the anticholinesterase activity of the lignans **1**–**5** displayed noticeable enzyme inhibition of **1** (IC_50_ = 85.03 ± 4.26 nM) and **5** (64.47 ± 2.75 nM) but lower activity of compounds **2**–**4** as compared to the reference drug donepezil. These findings were further emphasized by molecular docking of **1** and **5** with acetylcholinesterase (AChE). Rapid overlay chemical similarity (ROCS) and structure–activity relationships (SAR) analysis highlighted and rationalized the anti-AD capability of these compounds. Telomerase activation testing of the same isolates revealed 1.64-, 1.66-, and 1.72-fold activations in cells treated with compounds **1**, **5**, and **4**, respectively, compared to untreated cells. Our findings may pave the way for further investigations into the development of anti-Alzheimer and/or anti-ageing drugs from furofuranoid-type lignans.

## 1. Introduction

Lignans are a group of secondary metabolites widely encountered in significant levels in a variety of fruits and vegetables with wide structural diversity [1]. Among dietary components, lignans are abundant in barley, oats, buckwheat, millet, wheat, sesame seeds, rye, and flax. Fruits and vegetables such as grapes, kiwi, oranges, pineapple, asparagus, coffee, and tea contain significant amounts of lignans [2,3,4,5]. Lignans have been shown to protect against a variety of neuronal cell damage-related diseases, including Parkinson’s disease, Alzheimer’s disease (AD), stroke, and other neurodegenerative diseases. AD is an irreversible, progressive neurological illness that gradually ruins memory and thinking skills and the capacity to perform simple tasks [6,7]. The anti-Alzheimer’s actions of lignans, including protecting nerve cells and reducing dementia, are linked with their antioxidant capacity, lowering acetylcholinesterase (AChE) activity and vascular disease management in addition to manipulating many inflammatory cytokine pathways, such as NF-kB, NO, TNF-α, and IL-1β pathways [8,9,10,11]. There is evidence connecting the earliest symptoms of AD, such as memory and attention deficiency, to decreased brain acetylcholine levels and depletion of cholinergic neurons [12]. Thus, drugs that bring the brain’s acetylcholine levels back to normal provide promise for the treatment of AD.

A significant subclass of the lignan family of natural products comprise the furofuranoid lignans with reported beneficial effects on brain health, including anti-AD activity and anti-neurotoxicity [7,13]. Furofuranoid lignans from *Leucophyllum ambiguum* Bonpl. (Scrophulariaceae), *Leptadenia arborea* (Forssk.) Schweinf. (Asclepiadaceae), and *Sesamum indicum* L. (Acanthaceae) plant species exhibited potent AChE-inhibitory activity [13,14,15].

The furofuranoid lignans are widely encountered throughout the plant kingdom and have been isolated from 53 species in 41 genera of 27 plant families, including Acanthaceae [16]. *Anisacanthus virgularis* (Salisb.) Nees, Syn. of *Anisacanthus quadrifidus* var. *quadrifidus* (Acanthaceae), a native of tropical and subtropical regions of Mexico and West and South-Central Texas [17], is a popular cultivated ornamental shrub in many countries including the Middle East [18]. It can be recognized by its striking reddish–orange tubular flowers, which attract hummingbirds and butterflies [18]. *A. virgularis* has been shown to produce furofuranoid-type lignans, flavonoids, triterpenes, sterols, and iridoid glucosides [19,20,21] and has been investigated for its antiamoebic properties [20].

As a part of our ongoing effort [22,23] to find new anti-Alzheimer’s drugs and given the aforementioned anti-AChE properties of furofuranoid lignans and plant extracts rich in furofuranoid lignans [13,14,15], we continued to explore new anti-AChE furofuranoid lignans by considering phytochemical investigation of the ornamental plant *A. virgularis*.

On the other hand, AD and ageing are so intimately related that longer life expectancies will inevitably result in more AD cases [24]. Recent research has revealed several biological pathways as significant contributors to ageing, including telomere depletion, cellular senescence, genomic instability, stem cell exhaustion, mitochondrial malfunction, and epigenetic changes [25,26]. Therefore, examining the possible anti-ageing (telomere restoring) properties of the explored furofuranoid lignans from *A. virgularis* was also among the targets of the study.

In summary, the current study reports the structural elucidation of a new furofuranoid-type lignan (**1**), in addition to the identification of other six known compounds (**2**–**7**) from *A. virgularis* aerial parts. A possible biosynthetic mechanism for the furofuranoid-type lignans was suggested. The anticholinesterase activity and capability to improve telomerase activity in the normal melanocyte (HFB4) cell line of the lignans **1**–**5** were investigated by in vitro assay methods. In silico studies involving molecular docking, structural similarity to donepezil, and structure–property relationships were carried out to explore the active moiety or function group responsible for the anti-AChE activity and to highlight the potential of these compounds as scaffolds for the synthesis of other anti-AD drug candidates.

## 2. Results

### 2.1. Structural Elucidation

**Figure 1:**
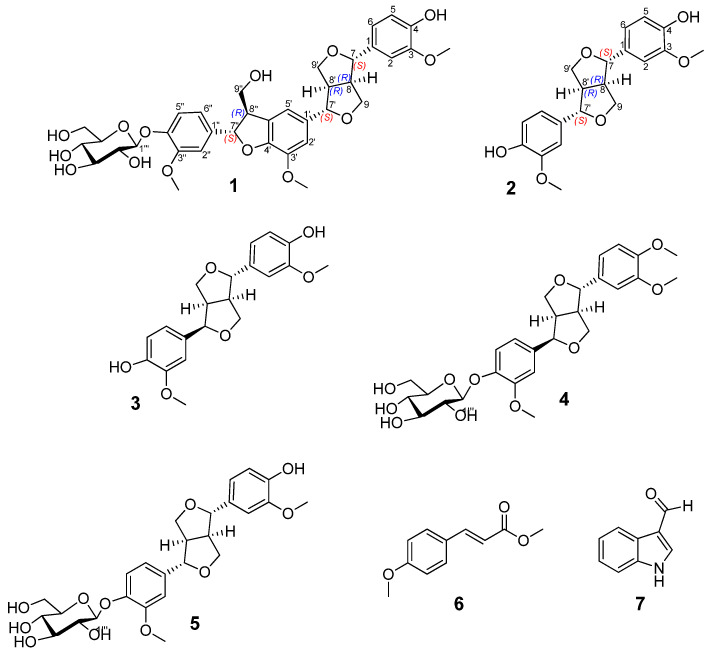
The structures of the purified compounds **1**–**7** from *A. virgularis*.

The dry powder of *A. virgularis* aerial parts was extracted by maceration in MeOH/H_2_O (8:2, *v*/*v*) at room temperature. The obtained extract was partitioned between distilled water and dichloromethane (DCM). The DCM-soluble extract was repeatedly chromatographed to afford seven compounds (**1**–**7**, Figure 1).

**Figure 2:**
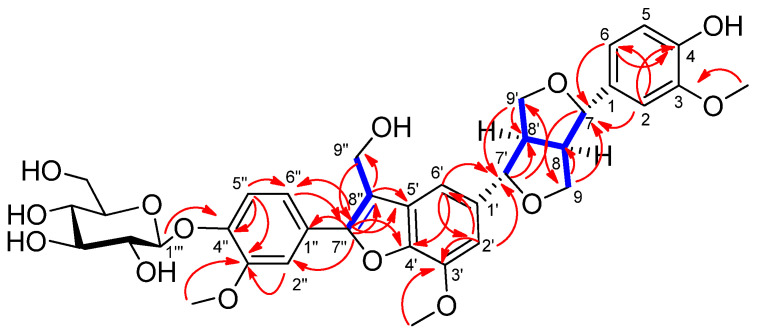
The key ^1^H–^1^H COSY (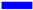) and HMBC (H
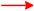
C) correlations of compound **1**.

Compound **1** was obtained as a yellow residue. Its HRESIMS spectrum (Figure S2) exhibited an ion peak at *m*/*z* 721.2442 [M + Na]^+^ (Calcd for C_36_H_42_NaO_14_, 721.2472) together with a positive FAB-MS [M + Na]^+^ ion peak at *m*/*z* 721 (Figure S1), indicating its molecular formula as C_36_H_42_O_14_. The ^1^H NMR data of **1** (Table 1) exhibited four aliphatic methine proton signals at *δ*_H_ (4.74 (d, *J* = 4.5 Hz), 4.72 (d, *J* = 4.5 Hz), 3.17 (m), and 3.15 (m). Figures S3–S5) and exhibited HSQC correlations with the carbons at *δ*_C_ 87.6 (C-7), 87.4 (C-7′), 55.6 (C-8), and 55.3 (C-8′), respectively. Two pairs of methylene proton signals were found [(*δ*_H_ 4.23 (ddd, *J* = 11.5, 7.0, 2.0 Hz) and 3.85 overlapped (ov)], and [*δ*_H_ 4.25 (ddd, *J* = 11.5, 7.0, 2.0 Hz) and 3.84 (ov)]. The first pair exhibited an HSQC correlation with the carbon at *δ*_C_ 72.7 (C-9), and the second pair correlated with the carbon at *δ*_C_ 72.5 (C-9′). These findings suggest the presence of two tetrahydrofurofuran moieties [27]. Signals of a tri-substituted phenyl ring [*δ*_H_ 6.95 (1H, d, *J* = 1.5 Hz, H-2), 6.77 (1H, d, *J* = 8.0 Hz, H-5) and 6.82 (1H, dd, *J* = 8.0, 2.0 Hz, H-6)], together with signals of a tetra-substituted phenyl ring [*δ*_H_ 6.92 (1H, brs, H-2′), 6.90 (1H, brs, H-6′)] were observed in the aromatic region of the ^1^H NMR spectrum (Figures S3–S5). The ^13^C NMR spectrum (Figures S9 and S10) exhibited carbon peaks (among those observed in the range *δ*_C_ 112.1–149.0) assignable to C-1–C-6 and C-1′–C-6′ (Table 1) of these tri- and tetra-substituted phenyl moieties. In addition, protons and carbon signals of two methoxy groups [(*δ*_H_ 3.85/*δ*_C_ 56.7) and (*δ*_H_ 3.88/*δ*_C_ 56.8)] were observed in the aliphatic region of the NMR spectrum. These NMR data of **1** are similar to those of the furanofuranoid lignan pinoresinol (**2**) (see comparison in Table S1). Additionally, ^1^H NMR signals (*δ*_H_ 7.03 (1H, d, *J* = 1.6 Hz, H-2″), 7.14 (1H, d, *J* = 8.5 Hz, H-5″), and 6.93 (1H, dd, *J* = 8.5, 1.5 Hz, H-6″)) exhibited HSQC correlations to the carbons *δ*_C_ 111.1, 118.0, and 119.3 and indicated the presence of an additional tri-substituted phenyl ring. Moreover, two distinct methine proton signals (*δ*_H_ 5.58 (d, *J* = 5.5 Hz) and 3.48, (m)) and a methylene proton signal (*δ*_H_ 3.78 and (dd, *J* = 11.0, 7.0 Hz) and 3.85) exhibited HSQC correlations with the respective carbons (*δ*_C_ 88.6 (C-7″), 55.6 (C-8″), and 64.9 (C-9″) (Figures S11–S13) and are assignable for a substituted propanol moiety forming a dihydrofuran ring [28]. The 1D NMR spectra also exhibited methoxy-group NMR data (*δ*_H_ 3.83 (3H, s)/(*δ*_C_ 56.7). Finally, a characteristic sugar anomeric proton signal (*δ*_H_ 4.89, d, *J* = 7.5 Hz, H-1‴) and a group of ^1^H–^1^H COSY coupled (Figures S6–S9) aliphatic signals (*δ*_H_ 3.39–3.87, H-2‴–H6‴, Table 1), which exhibited HSQC correlations with an anomeric carbon (102.7) and the aliphatic carbons *δ*_C_ 74.9 (C-2‴), 778.2 (C-3‴), 71.3 (C-4‴), 77.9 (C-5‴), and 62.4 (C-6‴), respectively (Figure S11), are characteristics for a β-d-glucosyl moiety [21]. These NMR data indicated the presence of a glucosylated dihydroconiferyl alcohol moiety, which together with the aforementioned pinoresinoyl moiety participates in the biogenesis of compound **1**. The connectivity of these structural components of **1** was established by ^1^H–^1^H COSY correlations (Figures S6–S9), as represented by blue bold bond (Figure 2), and HMBC correlations (Figures S14–S16) as represented by red arrows in (Figure 2).

Regarding the stereochemistry of **1**, it has been reported that naturally occurring furofuranoid lignans adopt a *cis*-fused configuration of the 7,9′:7′,9-diepoxy moiety [29,30]. According to the literature, the relative configuration of C-7/C-8 and C-7′/C-8′ of 8-H-type furofuranoid lignans was solved by using the rule of the chemical shift differences of the geminal protons of C-9 and C-9′ (Δ*δ*_H-9_ and Δ*δ*_H-9′_) [31], in which the 7-H/8-H *trans* and 7′-H/8′-H *trans* compounds showed Δδ_H-9_ and Δδ_H-9′_ = 0.30–0.40. Applying this rule to the fused di-tetrahydrofuran moiety of compound **1** revealed that Δ*δ*_H-9′_ and Δ*δ*_H-9_ were 0.41 and 0.38, respectively (Table S2), emphasizing that the 7′-H/8′-H and 7-H/8-H are adopting *trans* orientation [31]. The compounds **2**–**5** are also following the rule of the values of Δ*δ*_H-9_ and Δ*δ*_H-9′_ (Table S2).

**Scheme 1:**
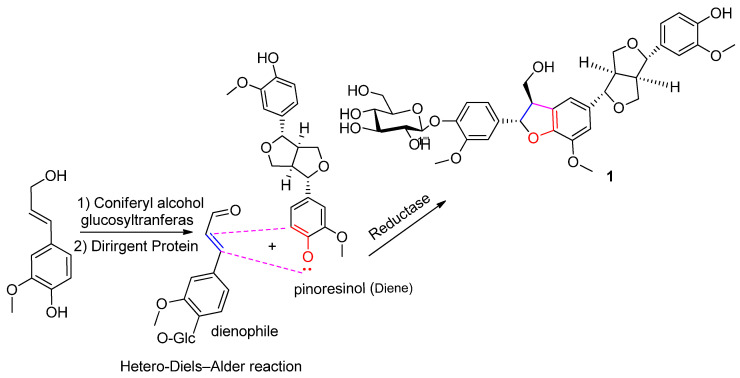
Suggested biosynthetic pathway for compound **1**.

The aryl and methylol substituents at the stereocenters C-7″ and C-8″ were suggested the more stable *trans* configuration, as determined from the coupling constant (*J*_7”,8”_ = 5.5 Hz), which is identical to the coupling (*J*_7,8_ = 5.4 (measured in CD_3_OD) shown by identical protons in dehydrodiconiferylalcohol-4-*β*-d-glucoside [28], where the 7″*S*, 8″*R* type of chirality is consistent with the biogenetic origin of the 7″-aryl-8″ methylol part of the molecule from the *trans*-coniferyl alcohol; biosynthetically, compound **1** could be originated from the cycloaddition reaction and oxidative coupling of the major *E*-isomer coniferyl alcohol with pinoresinol [(**2**), Scheme 1].

Furthermore, the planar structure of the 7″-aryl-8″-methyloyldihydrobenzofuran part of compound **1** is identical to the 7-aryl-8-methyloyldihydrobenzofuran part of the structure of gardenifolins a–h (Figure S34). Among those structures, the isomers (gardenifolins a, c, e, and g) with the configuration 7*S* exhibited specific optical rotation ([α]_D_) of positive values. On the other hand, the reverse isomers gardenifolins b, d, f, and h with 7″*R* configurations exhibited [α]_D_ with negative values [32]. Correspondingly, the positive value of the optical rotation ${\left[\alpha \right]}_{\;D}^{25}$ +25 of **1** is also consistent with the proposed relative configurations 7″*S* and, consequently, 8″*R* of compound **1**. The stereo-structure of **1** (Figure 1) was, thus, proposed by comparing the NMR and alpha-D data with findings reported in the literature as discussed above; nevertheless, a circular dichroism (CD) investigation is still required to establish **1**′s absolute configuration. Since **1** was first reported in nature, its protons and carbons were assigned from 1D and 2D NMR spectra (Figures S3–S16) and comparisons with data of known compounds, and it was given the name anisacanthin (**1**).

The structure of the known compounds (**2**–**7**, Figure 1) were established based on data obtained from NMR (1D and 2D), EI-MS, and FAB-MS spectroscopic data and comparisons with reported values as pinoresinol (**2**) [33], epipinoresinol (**3**) [34], phillyrin (**4**) [35], pinoresinol 4-*O*-*β*-d-glucoside (**5**) [36], *p*-methoxy-*trans*-methyl cinnamate (**6**) [37], and 1H-indole-3-carboxaldehyde (**7**) [38]. As far as we know, compounds **5**–**7** are initially reported in the genus *Anisacanthus*, while compounds **2** and **3** are first reported in this species.

**Scheme 2:**
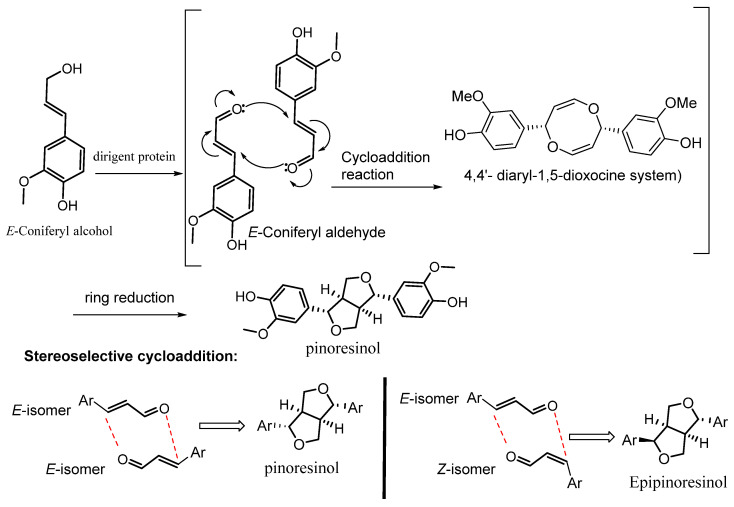
Suggested biosynthetic pathway for pinoresinol (**2**) and epipinoresinol (**3**).

Compounds **1**–**5** could be generated by a common biosynthetic pathway from the dimerization of two coniferyl alcohol units with the guide of dirigent protein [39]. The possible mechanism for the formation of final compounds is the cycloaddition reaction as depicted in Scheme 2. We anticipate the cycloaddition as an alternative mechanism to the early suggested free radical mechanism [40]. The current mechanism better explains the formation of the pinoresinol and epipinoresinol system, whereas the free radical mechanism may lead to many side products. Upon the presence of dirigent protein as an oxidant, the coniferyl alcohol (alley alcohol type) is oxidized in situ to coniferyl aldehyde. Every two molecules of generated aldehyde (major *E*-isomer) undergo a cycloaddition reaction followed by ring contraction to form the final pinoresinol skeleton. For the epipinoresinol system (not quite common), it could be obtained by cycloaddition of an *E*-isomer with minor *Z*-form. Regarding the formation of compound **1**, it is clear that under the effect of coniferyl alcohol glucosyltransferase and the oxidant dirigent protein, the α,β-unsaturated aldehyde (glucosylated coniferyl) was formed. The α,β-unsaturated aldehyde (as dienophile) interacts with the phenolic aryl of the pinoresinol ring (as diene) under the known hetero-Diels–Alder reaction in a cycloaddition pathway to finally form compound **1** (Scheme 1).

### 2.2. Bioactivity of the Isolated Lignans ***1**–**5***

As part of our ongoing interest in exploring anti-AD and anti-ageing drug candidates from natural sources [22,23], encouraged by the reported AChE-inhibitory effects of furofuranoid lignans [13,14,15], the isolated lignans **1**–**5** were evaluated for anti-AChE activity as well as their ability to boost telomerase activity.

#### 2.2.1. Anti-AChE Activity

The lignan-type compounds **1**–**5** were investigated in vitro against AChE using the modified Ellman’s method [41]. Compounds **1** and **5** showed significant enzyme inhibition activity with IC_50_ 85 ± 4 and 64 ± 3 nM, respectively, compared to the standard drug donepezil (IC_50_ 31 ± 3 nM) (Table 2). Compounds **2**–**4** showed relatively weak enzyme inhibition activity (IC_50_ 292 ± 9, 242 ± 9, 242 ± 9, and 279 ± 11 nM, respectively) (Table 2).

#### 2.2.2. Anti-Ageing Activity

Telomere shortening has been associated with multi-diseases such as cellular ageing and telomerase-related gene mutations. Telomerase activators are, thus, critically anti-ageing and a possible treatment for telomerase-dependent diseases [42]. Different studies have shown that telomerase activity in mice and rats can overturn the ageing process. For example, up to 10% longer life spans in experimental mice are observed when telomerase activation and overexpression of telomerase reverse transcriptase (TERT) occur in different tissues as compared to wild-type mice [43]. Another study showed that increased TERT expression in cancer-resistant mice delayed ageing and prolonged survival by 40% [44,45]. Telomerase activation is, thus, a potentially useful strategy to tackle age-related diseases and to combat ageing.

In the present study, we assessed the in vitro anti-ageing activity of the lignan-type compounds **1**–**5** utilizing a TERT enzyme assay (Table 3). The normal melanocyte cell line (HFB4) was used as a model system in this study [46], and curcumin (1 μM) was used as a standard telomerase activator [47]. According to the report, curcumin has made it possible for model organisms to live longer, reduced the signs of ageing, and slowed the onset of age-related illnesses where cellular senescence is a direct cause [47].

The results (Table 3) showed that compound **4** triggered a 1.71-fold increase in telomerase activity followed by compounds **5** and **1** with a 1.66 and 1.64-fold increases in telomerase activity. Telomerase activity triggered by compounds **2** and **3** was a little weak as compared to the untreated cells, and the standard curcumin induced a 1.62–fold increase in telomerase activity as compared to the control untreated cells at the examined concentration (1 μM).

### 2.3. Molecular Modelling Studies

The creation of compounds as anticholinesterase drugs is frequently aided by molecular modelling techniques including molecular docking and rapid overlay chemical similarity (ROCS). These techniques can shorten development time and costs by offering valuable insights into the design of new inhibitors [48].

#### 2.3.1. Molecular Docking

**Figure 3:**
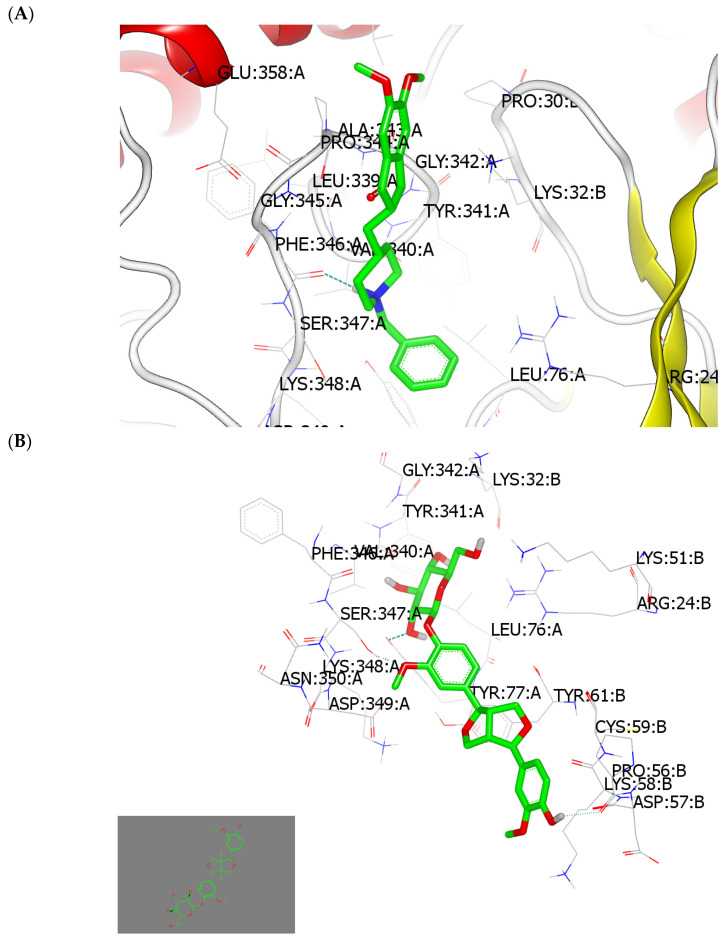

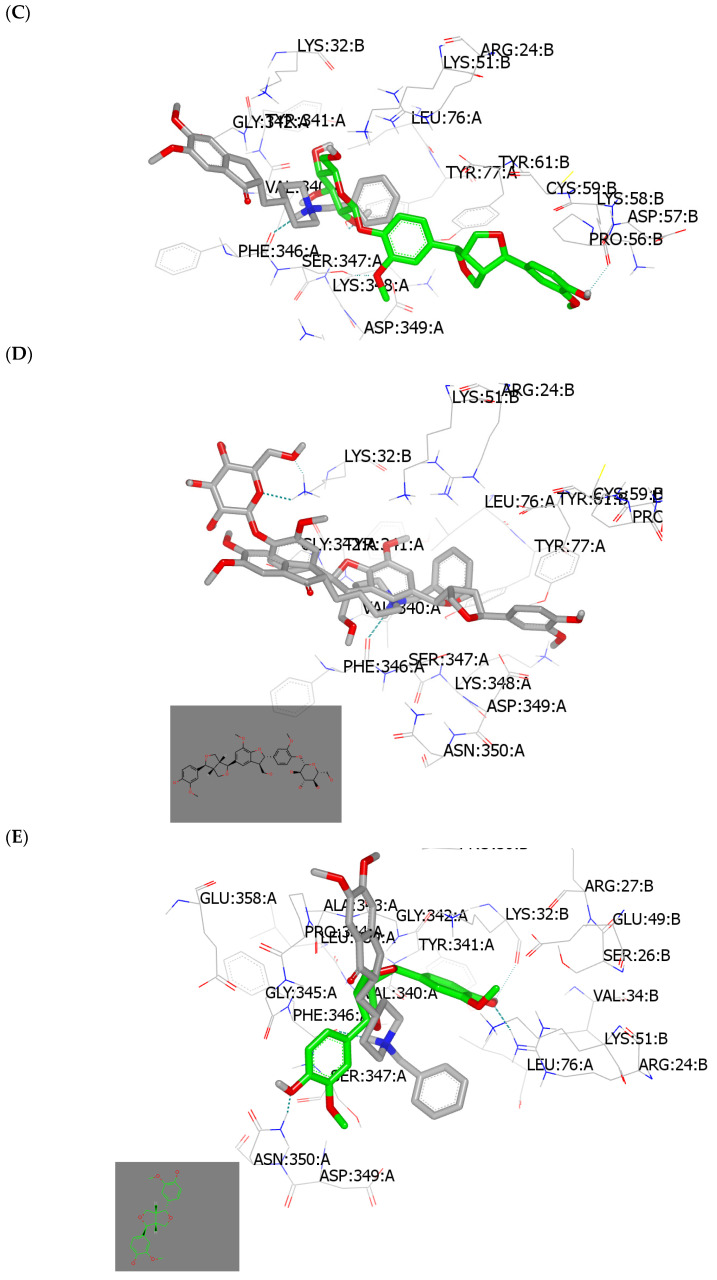
(**A**–**E**) Visual representation of compounds docked with PDB ID: 4BDT: (**A**) donepezil; (**B**) compound **5** formed HBs with SER: 347A, Tyr:77A, and Pro: 56A; (**C**) compound **5** (green colour) overlay with donepezil (grey colour); (**D**) compound **1** represented HBs with SER: 347A, Gly: 324A, and Lys: 32A and is overlaid with donepezil; (**E**) compound **3** overlay with donepezil. H-bonds are shown with green dotted lines.

To understand the mechanism of AChE inhibition by isolated compounds, molecular docking of compounds (**1**–**5**) and the standard drug donepezil with the human cholinesterase protein (PDB ID: 4BDT) was carried out [49]. The AChE receptor consists of two regions: hydrophobic cavities and polar cavities. The hydrophobic cavity was delimited by Tyr: 337A, Pro: 446A, Tyr: 449A, Met: 443A, and Trp: 439A. The polar cavity includes peptide VAL: 340A—PHE: 346A. Donepezil was docked by its amino group with the receptor through the formation of one hydrogen bond (HB) with PHE: 346A (HB donor) and hydrophobic–hydrophobic interaction (Figure 3A). Among the isolated compounds, compound **5** showed HB formation with SER: 347A through the oxygen of the methoxy group with internal phenyl. Furthermore, the OH of C-2 on the sugar moiety participated in HB formation (as acceptor) with Tyr:77A. Moreover, the OH of the outer phenyl ring interacted with the receptor cavity through the formation of HB with Pro: 56A as shown in (Figure 3B). The overlay of compound **5** (grey colour) with donepezil (green colour) illustrated different poses while they formed HB with the same peptide moiety (with SER: 347A and Phe: 346A, respectively (Figure 3C).

Compound **1** presented HB with SER: 347A through the oxygen of the furofuranoid ring. The oxygen of anomeric carbon and H of OH at C1 in the glucose moiety formed two HBs with Gly: 324A and Lys: 32A, respectively. The other part of the compound exhibited hydrophobic–hydrophobic interaction (Figure 3D). Additionally, the presence of a phenylpropanoid moiety as a spacer between the furofuranoid skeleton and sugar decreases the activity of compound **1** compared to that of compound **5**. This may be attributable to the large volume of the total surface area of compound **1** which is not a quick fit inside the receptor cavity, together with the lack of HB formation with TYR: 77A as shown in compound **5** (Figure 3D). Compound **3**, an isomer of compound **5**, exhibited a different binding mode and pose as compared to compound **5**. It participated in extra HBs with Asn: 350 A, Arg: 24A, and weak HB with Lys: 32. Figure 3E displays compound **3** with donepezil. The overlays of donepezil with compounds **3** and **4** are illustrated in Supplementary Materials (Figure S40A and Figure S40B, respectively).

#### 2.3.2. Structure–Activity Relationship Using Rapid Overlay Chemical Similarity (ROCS) and Shape Alignment

Shape similarity and shape alignment are methods used in virtual screening to foretell structure–activity links and lead hopping. In this regard, rapid overlay chemical similarity (ROCS) analysis, a useful command in the OpenEye program, was performed [50]. The results are illustrated in Table 4 using donepezil as a query. The similarity of our compounds to donepezil was sorted according to the Tanimoto combo (TC). The Tanimoto combo is the summation of the shape and colour Tanimoto. The maximum value of TC is 2. According to the generated data (Table 4), compound **2** represented higher similarity to donepezil than compounds **5** and **3**. The aglycon part of compound **1** showed the best TC to compound **4**.

**Figure 4:**
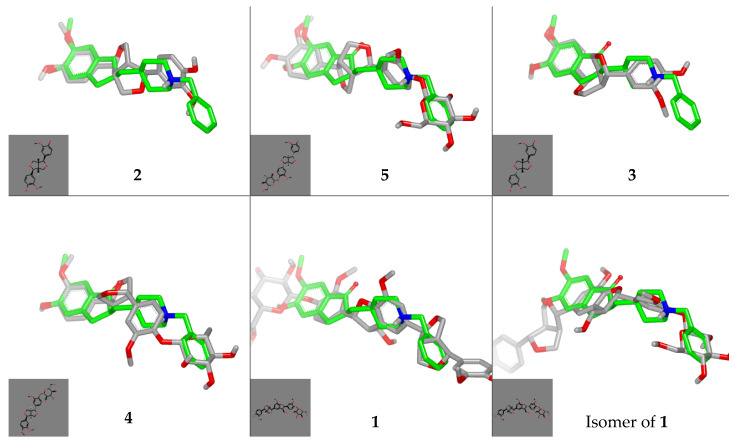
Visual representation by Vida application: ROCS analysis of isolated compounds **1**–**5** and the isomer of **1** in comparison to donepezil.

The shape alignments of compounds **1**–**5** and the isomer of **1** in comparison to donepezil are displayed in Figure 4. The absolute configuration of compounds is critical in shape alignment and molecular similarity. This evidence is very clear in the comparison of compound **2** with **3** and the comparison of compound **4** with **5**.

## 3. Discussion

Lignans are intriguing candidates for new anti-AD drugs due to their neuroprotective and cognitively improving qualities, which are mediated via their enhanced nerve protection, anti-inflammatory, and anti-AChE activity characteristics [7]. AChE inhibitors are used to treat Alzheimer’s disease because they help increase acetylcholine levels in the brain. The ability of lignans to suppress AChE has been well investigated. Furofuranoid lignans *Leucophyllum ambiguum* Bonpl., *Sesamum indicum* L., and *Leptadenia arborea* (Forssk.) Schweinf. plant species significantly reduced AChE activity [13,14,15].

A previous study has demonstrated that *A. virgularis* harbours plenty of Furofuranoid lignans [21], which can serve as antiacetylcholinesterase agents. Therefore, *A. virgularis* was considered for phytochemical isolation. Among the isolates, five compounds (**1**–**5**) were identified as furofuranoid lignans. Interpretation of the spectroscopic and physicochemical data and comparisons with the literature enabled substantiation of the structure of 1 as a furofuranoid lignan glucoside, which has not been reported hitherto. The other compounds were identified as furofuranoid lignans (pinoresinol (**2**), epipinoresinol (**3**), phillyrin (**4**), and pinoresinol 4-*O*-*β*-d-glucoside (**5**)), in addition to *p*-methoxy-*trans*-methyl cinnamate (**6**) and 1H-indole-3-carboxaldehyde (**7**), by comparing their spectroscopic data with reported data [33,34,35,36,37,38].

It is important to note that the free radical cyclization process was used to explain furofuranoid lignan biosynthesis [51]; however, this mechanism has been shown to depend on specific synthetic chemical conditions in a certain pH range to achieve optimal reaction conditions [51]. Herein, an alternate mechanism based on cycloaddition reaction for the explanation of furofuranoid lignan biosynthesis based on cycloaddition reaction was, thus, proposed since it is simpler than the free radical pathway and more closely resembles the metabolic process inside the plants [52].

The acetylcholinesterase-inhibitory effects of the lignans **1**–**5** were then studied by an in vitro enzyme-inhibitory assay and by computational methods. The best enzyme inhibition activity was shown by compounds **1** and **5** (IC_50_ 85 ± 4 and 64 ± 3 nM, respectively), as compared to donepezil (IC_50_ 31 ± 3 nM). The compounds **2**–**4** revealed weak enzyme inhibition (IC_50_ 292 ± 9, 242 ± 9, and 279 ± 11 nM, respectively). To the best of our knowledge, the AChE-inhibitory effect of compounds **1**, **3**, and **4** is shown first in this study, while the anticholinesterase activity of pinoresinol (**2**) and pinoresinol 4-*O*-*β*-d-glucoside (**5**) was shown before. Pinoresinol (**2**) (25 mg/kg, p.o.) improved memory decline in a dementia model caused by cholinergic blockade in a dose-dependent manner. Furthermore, pinoresinol (50 μM) aided the induction of hippocampal long-term potentiation (a cellular model of memory and learning), inhibited the activity of acetylcholinesterase in a concentration-dependent manner, and aided calcium influx into neuro2a cells [53]. Pinoresinol glucoside (**5**) exhibits a moderate inhibitory effect on the AChE enzyme (20.17 ± 0.44%) at a concentration of 250 µg/mL and a low inhibitory effect (11.02 ± 0.35%) on the butyrylcholinesterase enzyme at the same concentration [54].

The in silico studies offer a promising venue to explain the binding mode and pose for isolated compounds. These studies help researchers discover structure–activity relationships (SAR) and, thus, guide them to design and synthesize new derivatives based on isolated scaffolds. Molecular docking of the isolated compounds **1** and **5** and donepezil with the human acetylcholinesterase protein (PDB ID: 4BDT) clarified that both compounds interacted with the protein active site through the formation of multiple binding modes. Compounds **1** and **5** formed HBs with essential amino acids. Additionally, docking results revealed the importance of compound geometry and glycosylation in activity as described below in the SAR section. Furthermore, the shape alignments (ROCS analysis) demonstrate the close similarity of compounds **2** (aglycone scaffold) and **5** to donepezil. Guided by the data generated from the computational studies, the following SAR could be formulated:

- The sugar moiety at the C-4 position of the furofuranoid lignan skeleton is crucial in the compound’s potent activity provided that no HB can be formed with the oxygen atom of the hydroxyl group of C-4 of the furofuranoid skeleton;
- The HB formation of key amino acids with the hydroxyl groups at C-4 or C-4′ is better than their formation with the methoxy groups at C-3 or C-3′;
- The direct attachment of the sugar moiety at the C-4 position of the furofuranoid lignan system without a spacer (dihydrofuran moiety) is preferable;
- The ligands’ interaction with the key amino acids ARG: 24A, TYR: 77A, VAL: 340A, and PHE: 346A are crucial for the anticholinesterase activity.

Given the strong correlation between AD and ageing, longer life expectancies inevitably result in a rise in AD cases [24]; the potential of the furofuranoid lignans from *A. virgularis* in anti-ageing was investigated using an in vitro TERT enzyme assay. Phillyrin (**4**) increased telomerase activity by 1.71 times, whereas pinoresinol 4-*O*-*β*-d-glucoside (**5**) and anisacanthin (**1**) increased telomerase activity by 1.66 and 1.64 times, respectively. The anti-ageing properties of lignans were previously studied using *Caenorhabditis elegans* nematode as an animal model [55]. Another study measured the anti-ageing properties of the lignans by assessing the in vitro inhibition of the enzymes collagenase, tyrosinase, elastase, and hyaluronidase [56]. However, this is the first study reporting the anti-ageing activity of lignans using the TERT assay.

## 4. Materials and Methods

### 4.1. General Experimental Procedures

Sephadex LH-20 (25–100 µm, Sigma-Aldrich, Stockholm, Sweden), silica gel (E-Merk, Darmstadt, Germany) for the column, and silica gel 60 F_254_ aluminium sheets ((20 × 20, 0.2 mm, E-Merck, Germany) were used for thin-layer chromatography (TLC) analysis. For NMR analysis, the spectrometers Varian unity plus (Palo Alto, CA, USA) (400 MHz (^1^H NMR) and 100 MHz (^13^C NMR)) and Bruker Avance DRX-500 (Berlin, Germany) (500 MHz (^1^H NMR) and 125 MHz (^13^C NMR)) were employed. FAB-MS and EI-MS spectra were recorded on a JMS spectrometer (JEOL Ltd., Tokyo, Japan). FAB-MS positive ion mode with glycerol or *m*-nitrobenzyl alcohol, with or without NaCl, was used as the matrix. A Thermo Scientific LTQ/XL Orbitrap analyser (FTMS) was implemented for high-resolution electrospray ionization mass spectra (HRESIMS). A Jasco P-1020 polarimeter (Jasco Co. Ltd., Tokyo, Japan) was used for optical rotation measurements. Deuterated NMR solvents (DMSO-*d*_6_ (δ_H_ 2.50, δ_C_ 39.51), CD_3_OD (δ_H_ 3.31 and 4.78, δ_C_ 49.15), and CDCl_3_ (δ_H_ 7.24, δ_C_ 77.23)) were purchased from Nacalai Tesuque, Inc., Kyoto, Japan.

### 4.2. Plant Material

*A. virgularis* (Salisb.) Nees, Syn. of *Anisacanthus quadrifidus* var. *quadrifidus*, (Acanthaceae) fresh aerial parts were gathered from El-Orman Botanical Garden, Giza, Egypt. Authentication of the plant and preservation of the herbarium (USC-PH-Cog-02) were mentioned in our preceding paper [20].

### 4.3. Extraction and Isolation

*A. virgularis* dry aerial parts (1.8 Kg) were soaked in 80% aqueous methanol repeatedly (4 × 8 L) until exhaustion. All extracts were collected and dried under vacuum at 40 °C, yielding 320 g. The extract was fractionated by partitioning between water and DCM (5 L × 3) to yield DCM-soluble (DCM-F, 17 g) and aqueous-soluble (Aq-F, 300 g) fractions. First, the DCM-F was fractionated by vacuum liquid chromatography (VLC) with a non-polar solvent (*n*-hexane) followed by polar solvents (EtOAc and MeOH) to afford the fractions Ⅰ–X.

The fraction VI (96 mg), eluted with *n*-hexane/DCM (6:4, *v*/*v*), was chromatographed using silica gel column chromatography (CC) (1.5 × 100 cm, i.d.) with DCM, isocratically, and afforded five sub-fractions (VI-1–VI-5). The sub-fraction VI-1 (15 mg) was chromatographed on silica gel CC (1 × 25 cm, i.d.) with *n*-hexane/DCM (1:1, *v*/*v*), isocratically, to afford compound **6** (1 mg).

The fraction X (9 g) was chromatographed on silica gel CC (2 × 100 cm, i.d.), eluted with DCM, then DCM/MeOH gradients (9.5:0.5, 9:1, 8.5:1.5, and 8:2, *v*/*v*), to afford eight sub-fractions (X-a–X-h). The sub-fraction X-f (5.5 g) was chromatographed on silica gel CC (1.5 × 100 cm, i.d.), which was developed in the same manner, and afforded thirteen sub-fractions (f1–13). The sub-fraction X-f5 (199 mg) was chromatographed on silica gel CC (1 × 50 cm, i.d.) eluted with DCM, then DCM/MeOH gradients (9.5:0.5, 9:1, and 8.5:1.5, *v*/*v*), to afford compound **2** (2 mg) in the DCM/MeOH (9:1, *v*/*v*) eluate. The next sub-fraction (X-f6, 185 mg) was chromatographed on a Sephadex LH-20 (10 g) column (1 × 50 cm, i.d.) using isocratic elution with MeOH. The late eluate (7 mg) was further purified using silica gel CC (1 × 50 cm, i.d.) with DCM then DCM/MeOH (9:1, *v*/*v*) to afford compound **7** (1 mg). The sub-fraction X-f10 (199 mg) was chromatographed on silica gel CC (1.5 × 100 cm, i.d.) and eluted with EtOAc/DCM/MeOH/H_2_O (8:4:1.1:0.2) to afford compound **4** (25 mg). The sub-fraction X-f12 (200 mg) was chromatographed on silica gel CC (1 × 50 cm, i.d.) with DCM then DCM/MeOH gradients (9.5:0.5, 9:1, 8.5:1.5 and, 8:2, *v*/*v*) to afford compounds **3** (1.4 mg) and **5** (36 mg). The sub-fraction X-f13 (192 mg) was chromatographed on silica gel CC (1 × 50 cm, i.d.) and eluted by the same mode. The eluate with DCM/MeOH (9.5:0.5, *v*/*v*) (56 mg) was chromatographed on a Sephadex LH-20 (10 g) column (1 × 50 cm, i.d.) and eluted with MeOH isocratically. The late eluate (22 mg) was finally purified on silica gel CC (0.5 × 20 cm, i.d.) with DCM/MeOH (9:1, 8:2, *v*/*v*) to afford compound **1** (2 mg) in the latter mobile phase.

### 4.4. Spectroscopic Data of Isolated Compounds

- Anisacanthin (**1**): yellow residue; ${\left[\alpha \right]}_{\;D}^{25}$ +25 (c 0.08, CH_3_OH); ^1^H and ^13^C NMR data (Table 1); HRESIMS *m*/*z* 721.2442 [M + Na]^+^ (calcd for C_36_H_42_NaO_14_, 721.2472).
- Spectroscopic data of the known compound **2**–**7**: see Supplementary Materials.

### 4.5. Cholinesterase-Inhibitory Assay

The preparation of the sample was the same as has been previously described [22]. The AChE-inhibitory effect of the furofuranoid lignans **1**–**5** was determined using an AChE inhibitor screening kit (the Quanti-Chrome assay kit). The AChE enzyme (from *Electrophorus electricus*) hydrolyses the substrate acetylthiocholine, yielding thiocholine which reacts with Ellman’s reagent (5,5-dithiobis-(2-nitrobenzoic acid) (DTNB)) to produce 2-nitrobenzoate-5-mercaptothiocholine and 5-thio-2-nitrobenzoate, detectable at 412 nm [41]. The 96-well plate reaction was prepared according to the manufacturer’s protocol [22,57]. The standard drug donepezil was used as a positive control. The % inhibition of AChE activity was determined from the following formula:

% inhibition = 1 − ΔOD test compound/ΔOD no inhibitor × 100

where the Δ_OD_ test compound is the difference in optical density (OD) between the test compound and the enzyme reaction mixture, while the Δ_OD_ no inhibitor is the difference in OD between the enzyme reaction mixture and a control mixture without any inhibitor [22,57].

### 4.6. TERT Activity Assay

TERT activity assay was used for in vitro investigation of the compound’s anti-ageing properties [22]. The HFB4 (VACSERA, Giza, Egypt) was used as a model system for the study of amplification of telomers in response to our test compounds and curcumin. HFB4 cells were cultivated in RPMI-1640 medium enriched with heat-inactivated FBS (10%) and penicillin–streptomycin (100 U/mL). HFB4 cells (2 × 10^5^) were plated and incubated in a 5% CO_2_ humidified atmosphere at 37 °C for 24 h. The cells were then left untreated (negative control), treated with tested compounds and with 1 μM curcumin, a standard activator for TERT. The supernatant from the various conditions was collected after 24 h, centrifuged for 20 min at 1000× *g* at 4 ℃, and immediately submitted for the assay as previously described [22]. The different supernatants were added separately to a microtiter plate pre-coated with a TERT-specific antibody (Ab) and then incubated with a TERT-specific biotin detection Ab. Then, streptavidin/horseradish peroxidase (SABC) conjugate was added to all wells and incubated. Only the wells that contained TERT, biotin detection antibody, and enzyme-conjugated avidin exhibited a colour change after adding 3,3′,5,5′-tetramethylbenzidine (TMB) substrate for 15 min. The optical density (OD) was measured spectrophotometrically at 450 nm. The TERT concentration was determined by comparing the OD of the samples with the standard curve prepared according to the instructions of the manufacturer. The relative OD_450_ = (OD_450_ of the reaction well) − (OD_450_ of no treatment well). The standard curve was plotted by drawing the relative OD_450_ of each standard solution (Y) versus the respective concentration of the standard solution (X). The human TERT concentration of the samples was interpolated from the standard curve [58].

### 4.7. Statistical Analysis

Microsoft Excel 2010 and GraphPad Prism 7 were used for statistical analysis (the mean and standard deviation (SD)) as well as for the graphical display of the data. All experiments were conducted in triplicates.

### 4.8. Molecular Docking

To compare the binding poses with the highest binding affinities, the interaction between the ligands and AChE was examined using the crystal structures of AChE proteins (PDB ID: 4BDT) using Open Eye^®^ software, SantaFe, NM, USA (Academic licences 2021, Yaseen A. M. M. Elshaier) to develop various ligand conformations. Docking was conducted using Fred, and structure visualization was performed with Vida [59,60,61].

## 5. Conclusions

This study describes the isolation and characterization of a new furofuranoid-type lignan, anisacanthin (**1**), together with six known compounds (**2**–**7**) from the aerial parts of *A. virgularis*. A suggested mechanism for the biosynthesis of the furofuranoid lignans **1**–**5** was addressed in this work. Compounds **1**–**5** have been assessed for their inhibitory potential against AChE. Compounds **1** and **5** displayed significant inhibition of AChE. The practical findings were confirmed by molecular docking studies of **1** and **5** with AChE. The greater affinity of these compounds to the AChE peptide was also matched with great structural similarity, especially compounds **2** and **5**, as evident from ROCS analysis of isolated compounds **1**–**5** in comparison to donepezil. These molecules may find application in the treatment of Alzheimer’s disease and other neurodegenerative illnesses associated with brain acetylcholine deficit. Our study results highlight significant structure–activity relationship criteria that may be helpful in the design and synthesis of compounds that function as anti-AChE agents. This might occur if semisynthetic derivatives are created and examined using in vitro and in vivo techniques, as we intend to do in our future study.

## Figures and Tables

**Table 1:** ^1^H and ^13^C NMR spectral data of compound **1** (CD_3_OD, 500 and 125 MHz, respectively).

Position	*δ*_H_ in ppm, Multiplicity (*J* in Hz)	*δ*_C_ in ppm
1		136.1
2	6.95, d (*J* = 1.5)	110.9
3		149.1
4		147.3
5	6.77, d (*J* = 8.0)	116
6	6.92, dd (*J* = 8.0, 2.0)	119.3
7	4.74 ^a^, d (*J* = 4.5)	87.6
8	3.17 ^b^, m	55.6
9	4.23 ^c^, ddd (*J* = 11.5, 7.0, 2.0) 3.85 * (ov)^d^	72.7
1′	-	133.7
2′	6.92, brs.	112.1
3′		145.5
4′		149
5′		129.9
6′	6.90, brs.	116
7′	4.72 ^a^, d (*J* = 4.5)	87.4
8′	3.15 ^b^, m	55.3
9′	4.25 ^c^, ddd (*J* = 11.5, 7.0, 2.0)3.84 * (ov)^d^	72.5
1″	-	138.1
2″	7.03, d (*J* = 1.6)	111.1
3″	-	150.9
4″	-	147.6
5″	7.14, d (*J* = 8.5)	118
6″	6.82, dd (*J* = 8.5, 1.5)	120
7″	5.58, d (*J* = 5.5)	88.6
8″	3.48, m	55.6
9″	3.78, dd (*J* = 11.0, 7.0) 3.85 * (ov)^d^	64.9
1‴	4.89, d (*J* = 7.5)	102.7
2‴	3.47, dd (*J* = 10, 7.5)	74.9
3‴	3.39, t (*J* = 10)	78.2
4‴	3.39, m	71.3
5‴	3.45, m	77.9
6‴	3.68, ddd (*J* = 12.0, 5.5,1.5) 3.87 * (ov)	62.4
3′-OCH_3_	3.85, s	56.4
3″-OCH_3_	3.83, s	56.7
3-OCH_3_	3.88, s	56.8

* Multiplicity was not calculated because signals partially overlapped with methoxy signals. ^a,b,c^ exchangeable. (ov)^d^ means overlapped.

**Table 2:** In vitro AChE-inhibitory activity of compounds **1**–**5** isolated from *A. virgularis*.

Compounds	Concentrations (µM)				IC_50_ (nM) ^a^
	% Inhibition				
	0.01	0.1	1	10	
**1**	24 ± 1	61 ± 1	75 ± 2	83 ± 2	85 ± 4
**2**	26 ± 1	41 ± 1	60 ± 3	75 ± 2	292 ± 9
**3**	23 ± 1	44 ± 1	60 ± 2	82 ± 3	242 ± 9
**4**	26 ± 3	41 ± 2	64 ± 2	73 ± 2	279 ± 11
**5**	28 ± 3	59 ± 4	79 ± 1	88 ± 3	64 ± 3
**Donepezil**	34 ± 3	69 ± 3	79 ± 2	89 ± 2	31 ± 3

^a^ Results were expressed as mean ± SD.

**Table 3:** Results of the TERT enzyme assay of the isolated compounds **1**–**5** from *A. virgularis*.

Compound	TERT Concentration Mean ± SD in ng/mL	Relative Increase
**1**	2.8 ± 0.04	1.64
**2**	2.2 ± 0.03	1.28
**3**	2.0 ± 0.06	1.18
**4**	2.9 ± 0.06	1.72
**5**	2.8 ± 0.09	1.66
**Curcumin**	2.8 ± 0.03	1.62
**HFB4** (**control**)	1.78 ± 0.07	1

**Table 4:** TC score of isolated compounds in comparison to donepezil.

Compound Name	Tanimoto Combo	Shape Tanimoto	Colour Tanimoto
**Donepezil**	2.00	1.00	1.00
**2**	0.93	0.71	0.23
**5**	0.87	0.74	0.14
**3**	0.82	0.21	0.62
**Isomer of 1**	0.80	0.66	0.14
**4**	0.79	0.60	0.18
**1**	0.61	0.49	0.12

## Data Availability

The data is contained within the manuscript and Supplementary Materials.

## Supplementary Materials

The following supporting information can be downloaded at: https://www.mdpi.com/article/10.3390/plants13020150/s1, List of abbreviations; Figure S1: Positive FAB-MS spectrum of compound **1**; Figure S2: Positive HRESIMS spectrum of compound **1**; Figure S3: ^1^H NMR spectrum of compound **1** (CD_3_OD, 500 MHz); Expanded ^1^H NMR spectrum of compound **1** (CD_3_OD, 500 MHz); Figures S4 and S5: Expanded ^1^H NMR spectrum of compound **1** (CD_3_OD, 500 MHz); Figure S6: ^1^H–^1^H COSY spectrum of compound **1** (CD_3_OD, 500 MHz); Figures S7 and S8: Expanded ^1^H–^1^H COSY spectrum of compound **1** (CD_3_OD, 500 MHz); Figure S9: ^13^C NMR spectrum of compound **1** (CD_3_OD, 125 MHz); Figure S10: Expanded ^13^C NMR spectrum of compound **1** (CD_3_OD, 125 MHz); Figure S11: HSQC spectrum of compound **1**; Figures S12 and S13: Expanded HSQC spectrum of compound **1;** Figure S14: HMBC spectrum of compound **1**; Figures S15 and S16: Expanded HMBC spectrum of compound **1;** Figure S17: EI-MS spectrum of compound **2**; Figure S18: ^1^H NMR spectrum of compound **2** (CDCl_3_, 400 MHz); Figure S19: ^13^C NMR spectrum of compound **2** (CDCl_3_, 100 MHz); Figure S20: EI-MS spectrum of compound **3**; Figure S21: ^1^H NMR spectrum of compound **3** (CDCl_3_, 400 MHz); Figure S22: ^13^C NMR spectrum of compound **3** (CDCl_3_, 100 MHz); Figure S23: Positive FAB-MS spectrum of compound **4**; Figure S24: ^1^H NMR spectrum of compound **4** (DMSO-*d*_6_, 400 MHz); Figure S25: ^13^C NMR spectrum of compound **4** (DMSO-*d*_6_, 100 MHz); Figure S26: Positive FAB-MS spectrum of compound **5**; Figure S27: ^1^H NMR spectrum of compound **5** (CD_3_OD, 400 MHz); Figure S28: ^13^C NMR spectrum of compound **5** (CD_3_OD, 100 MHz); Figure S29: EI-MS spectrum of compound **6**; Figure S30: ^1^HNMR spectrum of compound **6** (CDCl_3_, 400 MHz); Figure S31: EI-MS spectrum of compound **7**; Figure S32: ^1^H NMR spectrum of compound **7** (CD_3_OD, 400 MHz); Figure S33: ^13^CNMR spectrum of compound **7** (CD_3_OD, 100 MHz); Figure S34: Structures of gardenifolins A, C, E, and G and their respective mirror images gardenifolins B, D, F, and H; Spectroscopic data of the isolated known compounds **2**–**7**; Figure S35: Dose–response curve of the anti-AChE assay of compound **1**; Figure S36: Dose–response curve of the anti-AChE assay of compound **2**; Figure S37: Dose–response curve of the anti-AChE assay of compound **3**; Figure S38: Dose–response curve of the anti-AChE assay of compound **4**; Figure S39: Dose–response curve of the anti-AChE assay of compound **5**; Figure S40: The overlay of donepezil with compounds **3** (A) and **4** (B); Table S1. ^1^H and ^13^C NMR spectral data of compound **1** (CD_3_OD, 500 and 125 MHz, respectively) and pinoresinol (**2**) (CDCl_3_, 400 and 100, respectively); Table S2. Chemical shift differences of H_2_-9 and H_2_-9′ (ΔδH-9 and ΔδH-9′) of compounds **1**–**5**. References [32,33,34,35,36,37,38,62] are cited in the supplementary materials.
