# Supplementary material for: Furofuranoid-Type Lignans and Related Phenolics from Anisacanthus virgularis (Salisb.) Nees with Promising Anticholinesterase and Anti-Ageing Properties: A Study Supported by Molecular Modelling
Source: Plants (Basel). 2024 Jan 5;13(2):150. doi: 10.3390/plants13020150 (PMC10820861; doi:10.3390/plants13020150)
Supplement: Supplementary file 1 [file plants-13-00150-s001.zip › plants-2704012-supplementary.pdf]

SUPPLEMENTARY MATERIALS

Furofuranoid-type lignans and related phenolics from *Anisacanthus virgularis* (Salisb.) Nees with promising anticholinesterase and anti-ageing properties: A study supported by molecular modelling

Mohamed A. A. Orabi[1,*,#], Reda A. Abdelhamid[2], Hanan Elimam[3], Yaseen A.M.M. Elshaier[4], A. A. Ali[5], Nayef Aldabaan[6], Abdulaziz Hassan Alhasaniah[7], and Mohamed S. Refaey[8,*,#]

[1] Department of Pharmacognosy, College of Pharmacy, Najran University, Najran 66454, Saudi Arabia; mohamedorabi@azhar.edu.eg; maorabi@nu.edu.sa.
[2] Department of Pharmacognosy, Faculty of Pharmacy, Al-Azhar University, Assiut-Branch, 71524 Assiut, Egypt; reda.ahmed@azhar.edu.eg.
[3] Department of Biochemistry, Faculty of Pharmacy, University of Sadat City, Sadat City, 32958, Egypt; hanan.elimam@fop.usc.edu.eg.
[4] Department of Organic and Medicinal chemistry, Faculty of Pharmacy, University of Sadat City, Sadat City, Menoufiya 32958, Egypt; yaseen.elshaier@fop.usc.edu.eg.
[5] Department of Pharmacognosy, Faculty of Pharmacy, Assiut University, Assiut 71526, Egypt; ahmed.ibrahim4@pharm.aun.edu.eg.
[6] Department of Pharmacology, College of Pharmacy, Najran University 1988, Najran 66454, Saudi Arabia; naaldabaan@nu.edu.sa
[7] Department of Clinical Laboratory Sciences, Faculty of Applied Medical Sciences, Najran University, Najran 66454, Saudi Arabia; Ahalhasaniah@nu.edu.sa (AHA).
[8] Department of Pharmacognosy, Faculty of Pharmacy, University of Sadat City, Sadat City, Menoufiya, 32958, Egypt; mohamed.said@fop.usc.edu.eg.
* Correspondence: mohamedorabi@azhar.edu.eg (MAAO); mohamed.said@fop.usc.edu.eg (MSR).
# Both authors contributed equally to this work

List of content

No.	**Contents**	**Page**
List of abbreviation		S1
Figure S1	Positive FAB-MS spectrum of compound 1	S2
Figure S2	Positive HRESIMS spectrum of compound 1	S3
Figure S3	^{1}H NMR spectrum of compound **1** (CD_3OD, 500 MHz).	S4
Figure S4	Expanded ^{1}H NMR spectrum of compound **1** (CD_3OD, 500 MHz).	S5
Figure S5	Expanded ^{1}H NMR spectrum of compound **1** (CD_3OD, 500 MHz).	S6
Figure S6	^{1}H–^{1}H COSY spectrum of compound **1** (CD_3OD, 500 MHz).	S7
Figure S7	Expanded ^{1}H–^{1}H COSY spectrum of compound **1** (CD_3OD, 500 MHz).	S8
Figure S8	Expanded ^{1}H–^{1}H COSY spectrum of compound **1** (CD_3OD, 500 MHz).	S9
Figure S9	^{13}C NMR spectrum of compound **1** (CD_3OD, 125 MHz).	S10
Figure S10	Expanded ^{13}C NMR spectrum of compound **1** (CD_3OD, 125 MHz).	S11
Figure S11	HSQC spectrum of compound **1**.	S12
Figure S12	Expanded HSQC spectrum of compound **1**.	S13
Figure S13	Expanded HSQC spectrum of compound **1**.	S14
Figure S14	HMBC spectrum of compound **1**.	S15
Figure S15	Expanded HMBC spectrum of compound **1**.	S16
Figure S16	Expanded HMBC spectrum of compound **1**.	S17
Figure S17	EI-MS spectrum of compound **2**	S18
Figure S18	^{1}H NMR spectrum of compound **2** ($CDCl_3$, 400 MHz).	S19
Figure S19	^{13}C NMR spectrum of compound **2** ($CDCl_3$, 100 MHz).	S20
Figure S20	EI-MS spectrum of compound **3**	S21
Figure S21	^{1}H NMR spectrum of compound **3** ($CDCl_3$, 400 MHz).	S22
Figure S22	^{13}C NMR spectrum of compound **3** ($CDCl_3$, 100 MHz).	S23
Figure S23	Positive FAB-MS spectrum of compound **4**.	S24
Figure S24	^{1}H NMR spectrum of compound **4** (DMSO-d_6, 400 MHz).	S25
Figure S25	^{13}C NMR spectrum of compound **4** (DMSO-d_6, 100 MHz).	S26
Figure S26	Positive FAB-MS spectrum of compound **5**.	S27
Figure S27	^{1}H NMR spectrum of compound **5** (CD_3OD, 400 MHz).	S28
Figure S28	^{13}C NMR spectrum of compound **5** (CD_3OD, 100 MHz).	S29

Figure S29	EI-MS spectrum of compound **6**.	S30
Figure S30	^{1}HNMR spectrum of compound **6** ($CDCl_3$, 400 MHz).	S31
Figure S31	EI-MS spectrum of compound **7**.	S32
Figure S32	^{1}H NMR spectrum of compound **7** (CD_3OD, 400 MHz).	S33
Figure S33	^{13}CNMR spectrum of compound **7** (CD_3OD, 100 MHz).	S34
Figure S34	Structures of gardenifolins A, C, E, and G and their respective mirror images gardenifolins B, D, F, and H.	S35
Spectroscopic data of the isolated known compounds **2–7**.		S36–37
Figure S35	Dose-response curve of the anti-AChE assay of compound **1**	S38
Figure S36	Dose-response curve of the anti-AChE assay of compound **2**	S39
Figure S37	Dose-response curve of the anti-AChE assay of compound **3**	S40
Figure S38	Dose-response curve of the anti-AChE assay of compound **4**	S41
Figure S39	Dose-response curve of the anti-AChE assay of compound **5**	S42
Figure S40:	The overlay of donepezil with compounds **3** (A) and **4** (B).	S43
Table S1.	^{1}H and ^{13}C NMR spectral data of compound **1** **(**CD_3OD, 500 and 125 MHz, respectively) and pinoresinol (**2**) ($CDCl_3$, 400 and 100, respectively).	S44
Table S2.	Chemical shift differences of H_2-9 and H_2-9′ (ΔδH-9 and ΔδH-9′) of compounds **1–5**.	S45
**References**		S44

List of abbreviation:

ACh: acetylcholine

AChE: acetyl cholinesterase

AD: Alzheimer's disease

ARG: arginine

CC: column chromatography

CNS: central nervous system

DCM: dichloromethane

DMSO: dimethyl sulphoxide

DTNB: 5,5-dithiobis-2-nitrobenzoic acid

EtOAc: ethyl acetate

GLY: glycine

HFB4: normal melanocytes cell line

LYS: lysine

MeOH: methanol

NMDA: N-methyl-D-aspartate

OD: optical density

PHE: phenylalanine

RPE: retinal pigment epithelial

SABC: streptavidin conjugated to Horseradish peroxidase

SER: serine

TERT: telomerase reverse transcriptase

TMB: 3,3′,5,5′ - tetramethylbenzidine

VLC: vacuum liquid chromatography

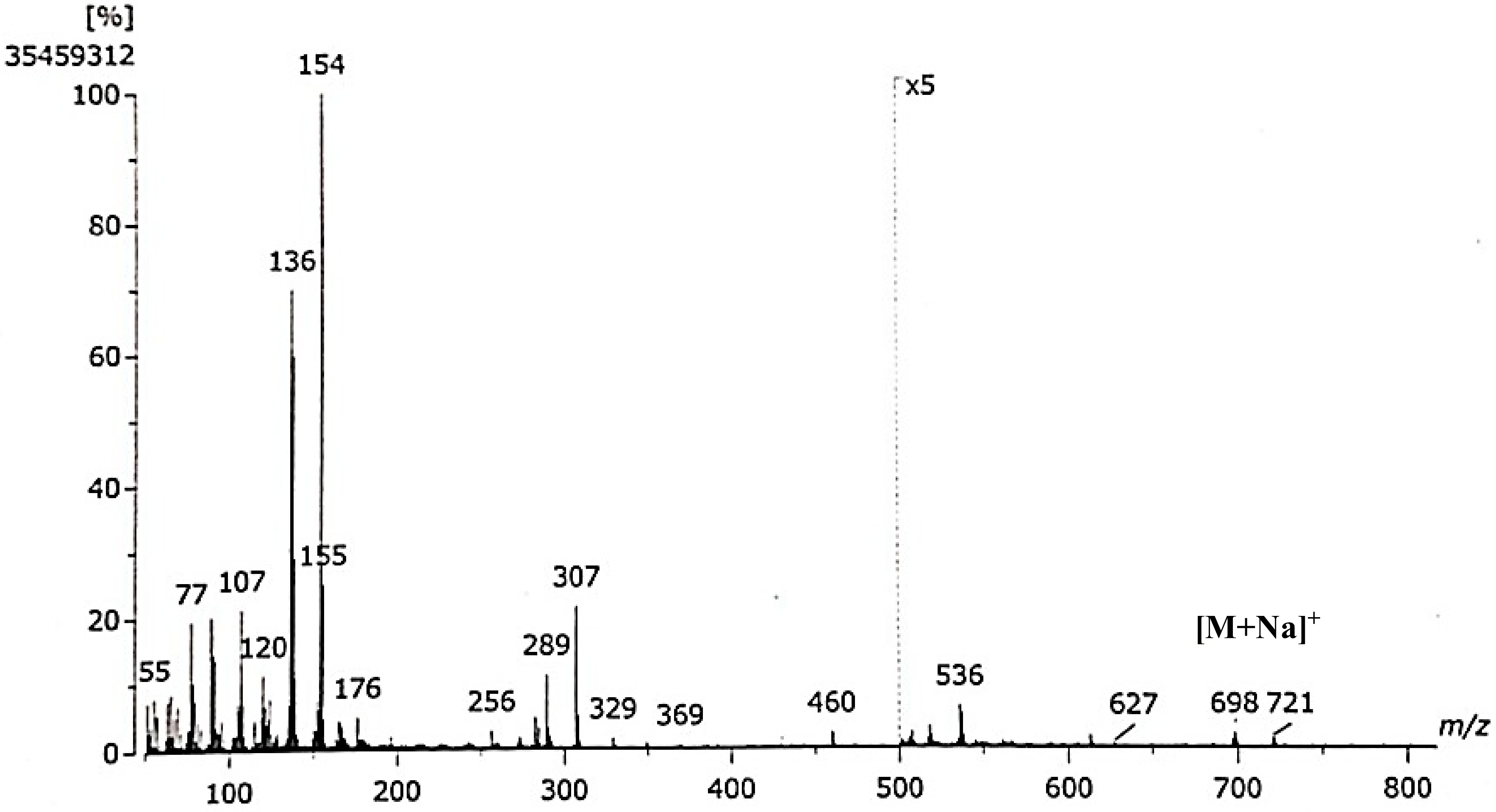

Figure S1. Positive FAB-MS spectrum of compound 1

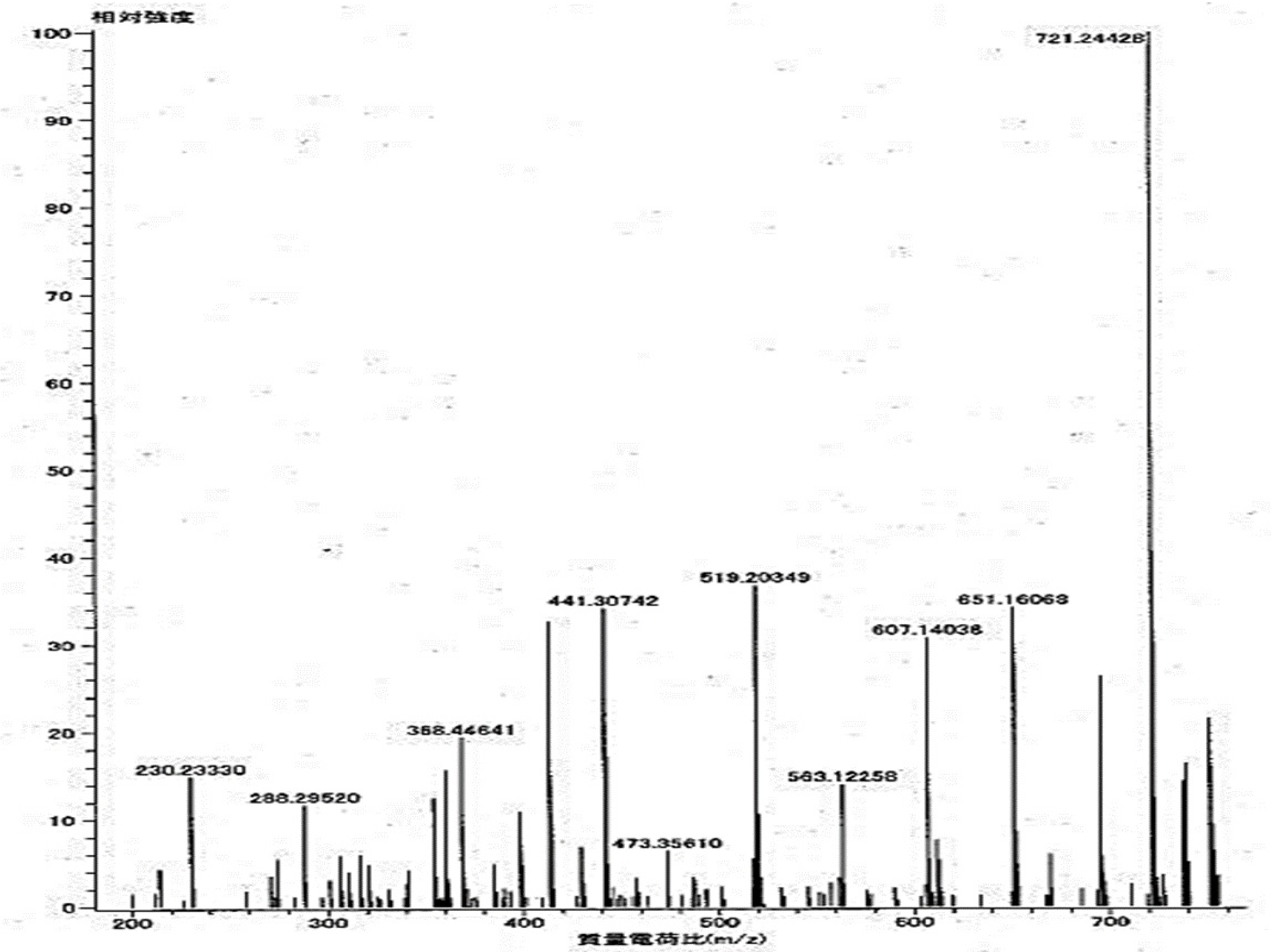

Figure S2. Positive HRESIMS spectrum of compound 1

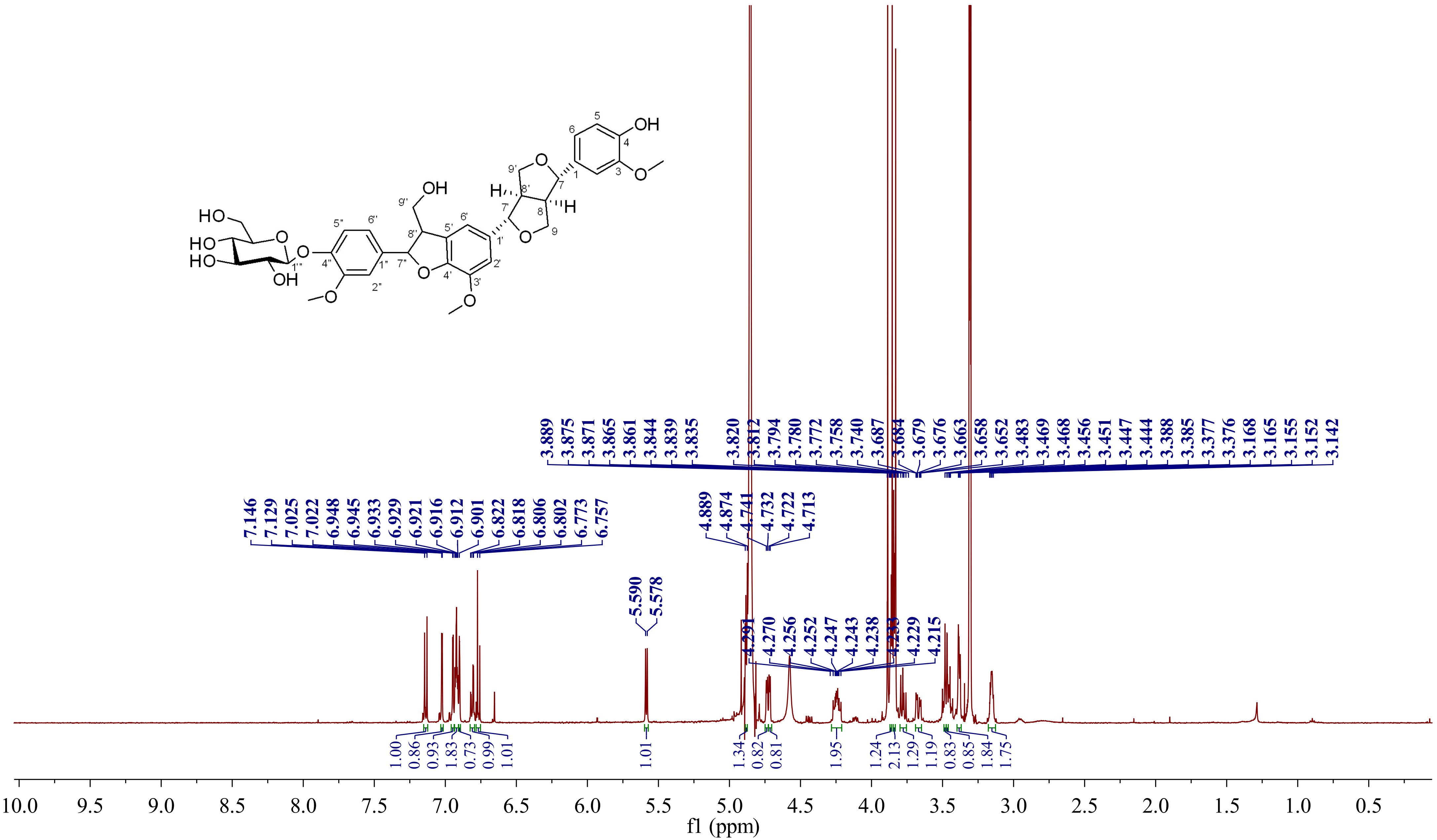

Figure S3. ^{1}H NMR spectrum of compound 1 (CD_3OD, 500 MHz)

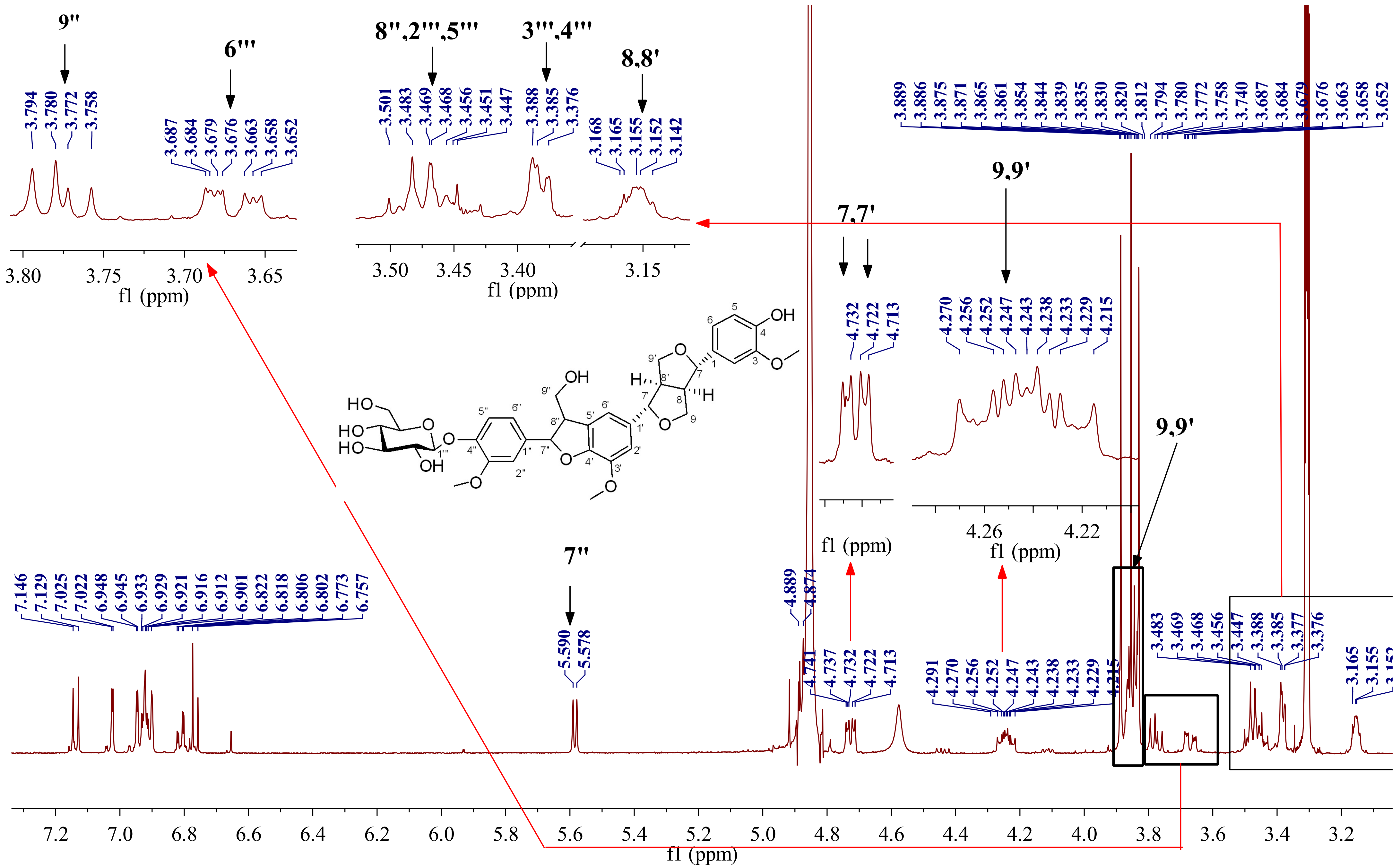

Figure S4. Expanded ^{1}H NMR spectrum of compound 1 (CD_3OD, 500 MHz)

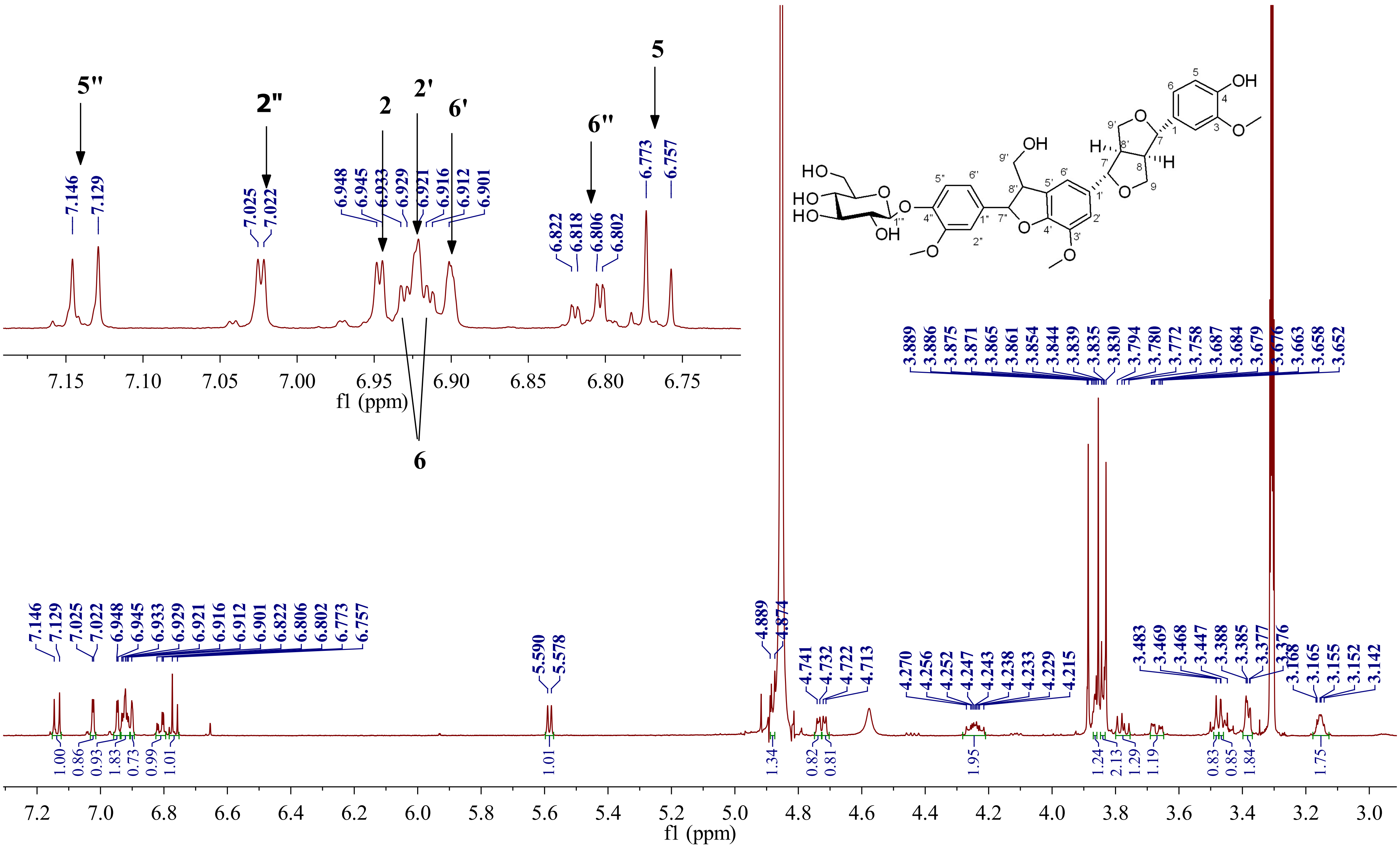

Figure S5. Expanded ^1H NMR spectrum of compound 1 (CD_3OD, 500 MHz)

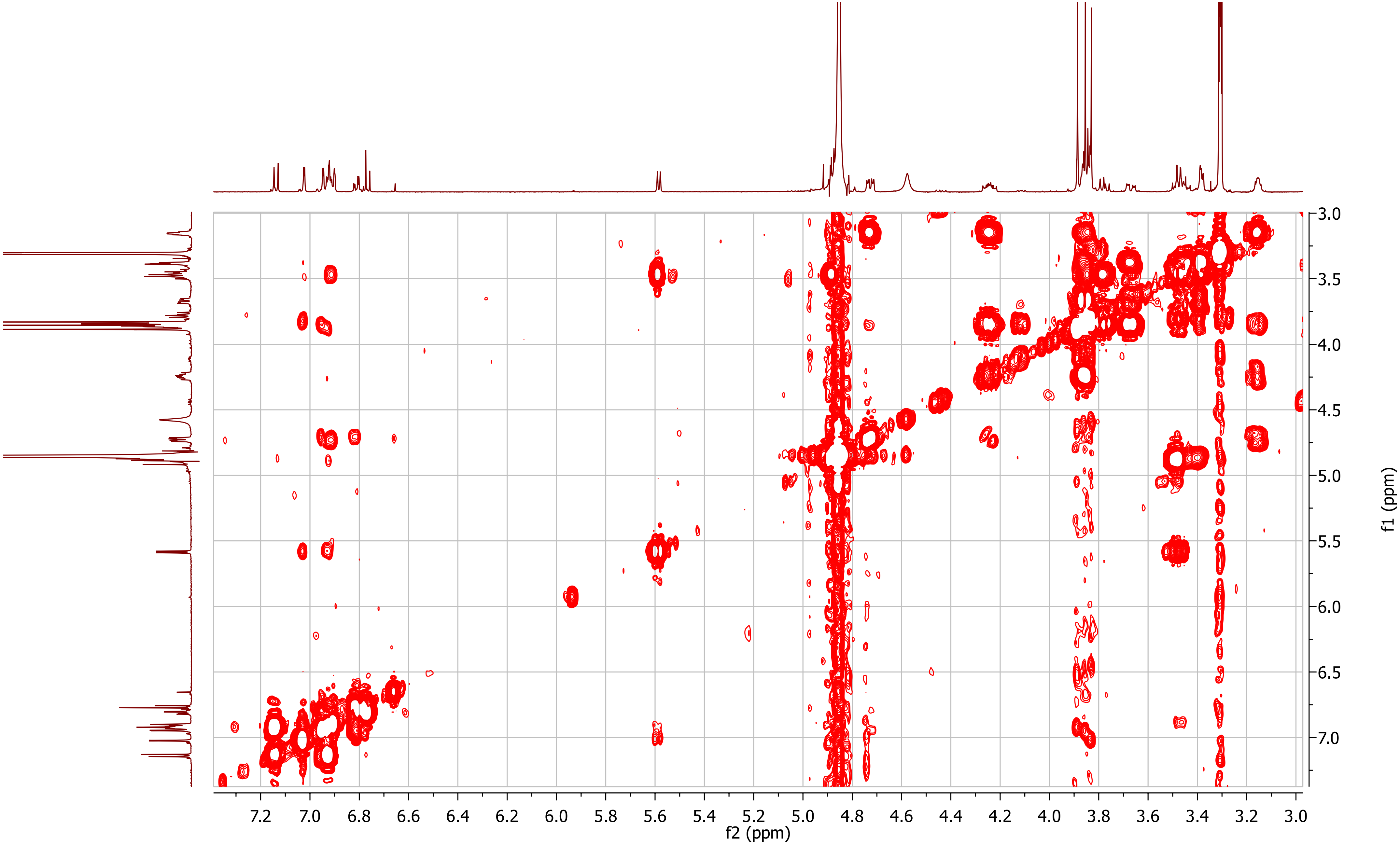

Figure S6. ^{1}H-^{1}H COSY spectrum of compound 1 (CD_3OD, 500 MHz)

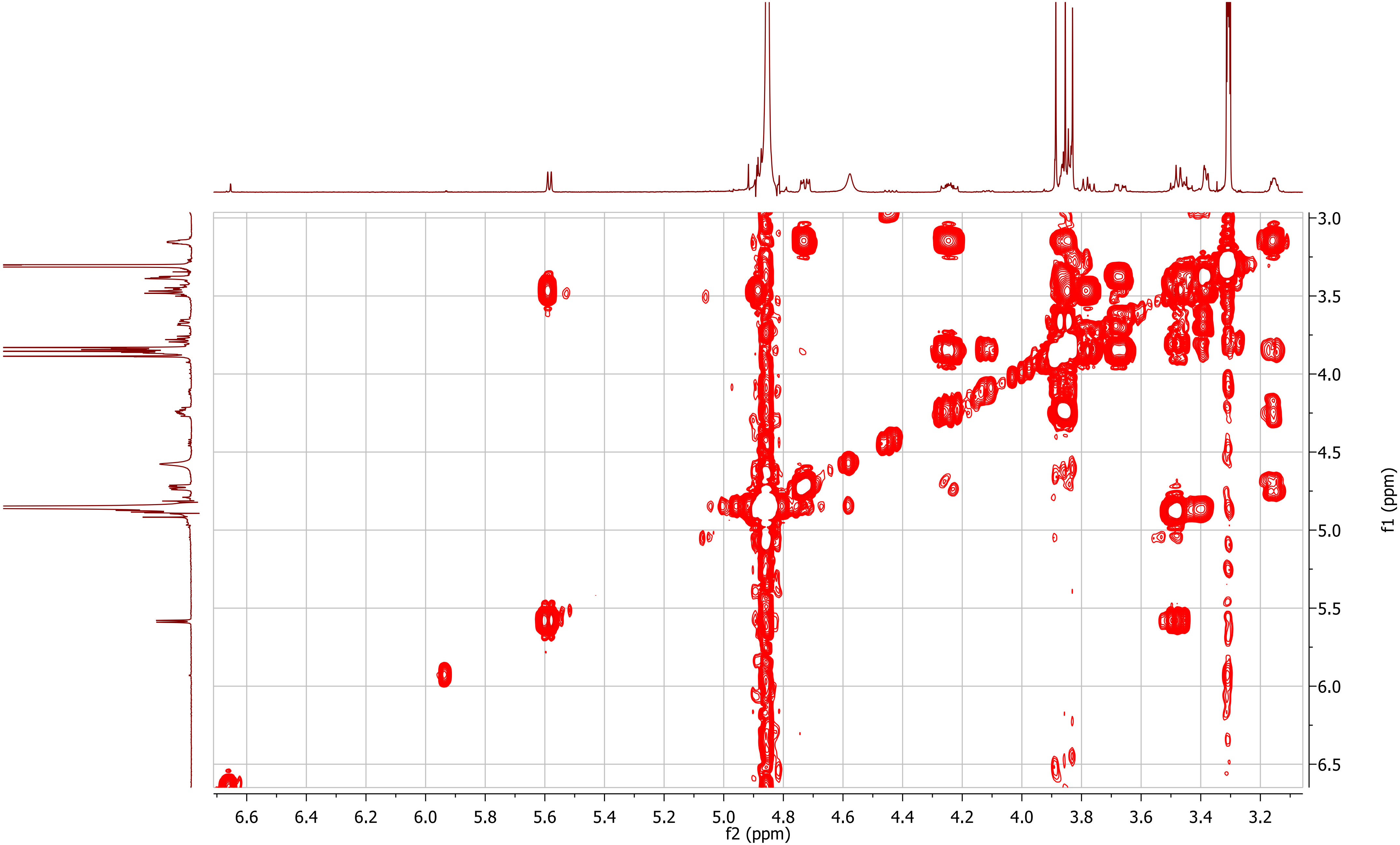

Figure S7. Expanded ^{1}H-^{1}H COSY spectrum of compound 1 (CD_3OD, 500 MHz)

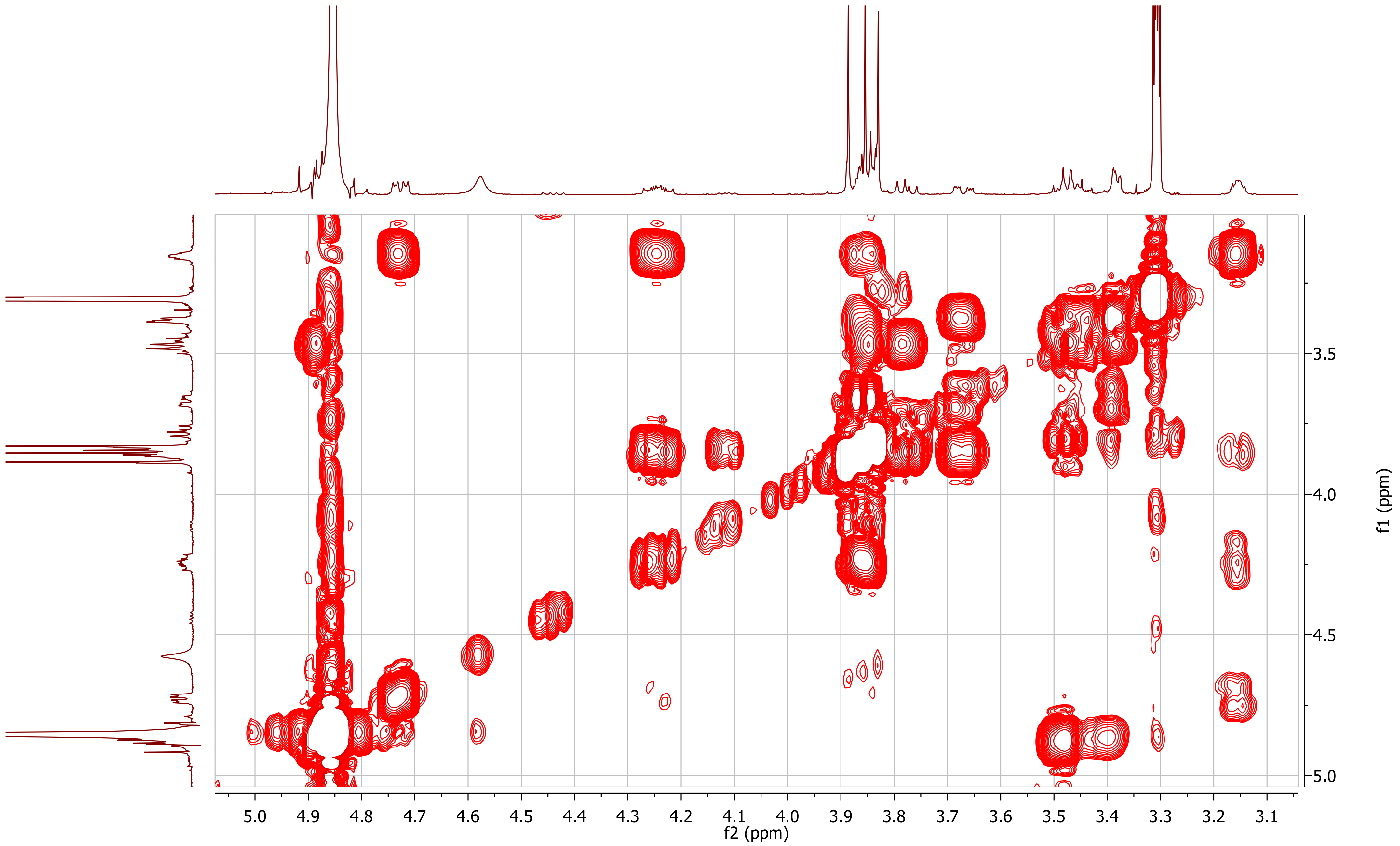

Figure S8. Expanded ^{1}H-^{1}H COSY spectrum of compound 1 (CD_3OD, 500 MHz)

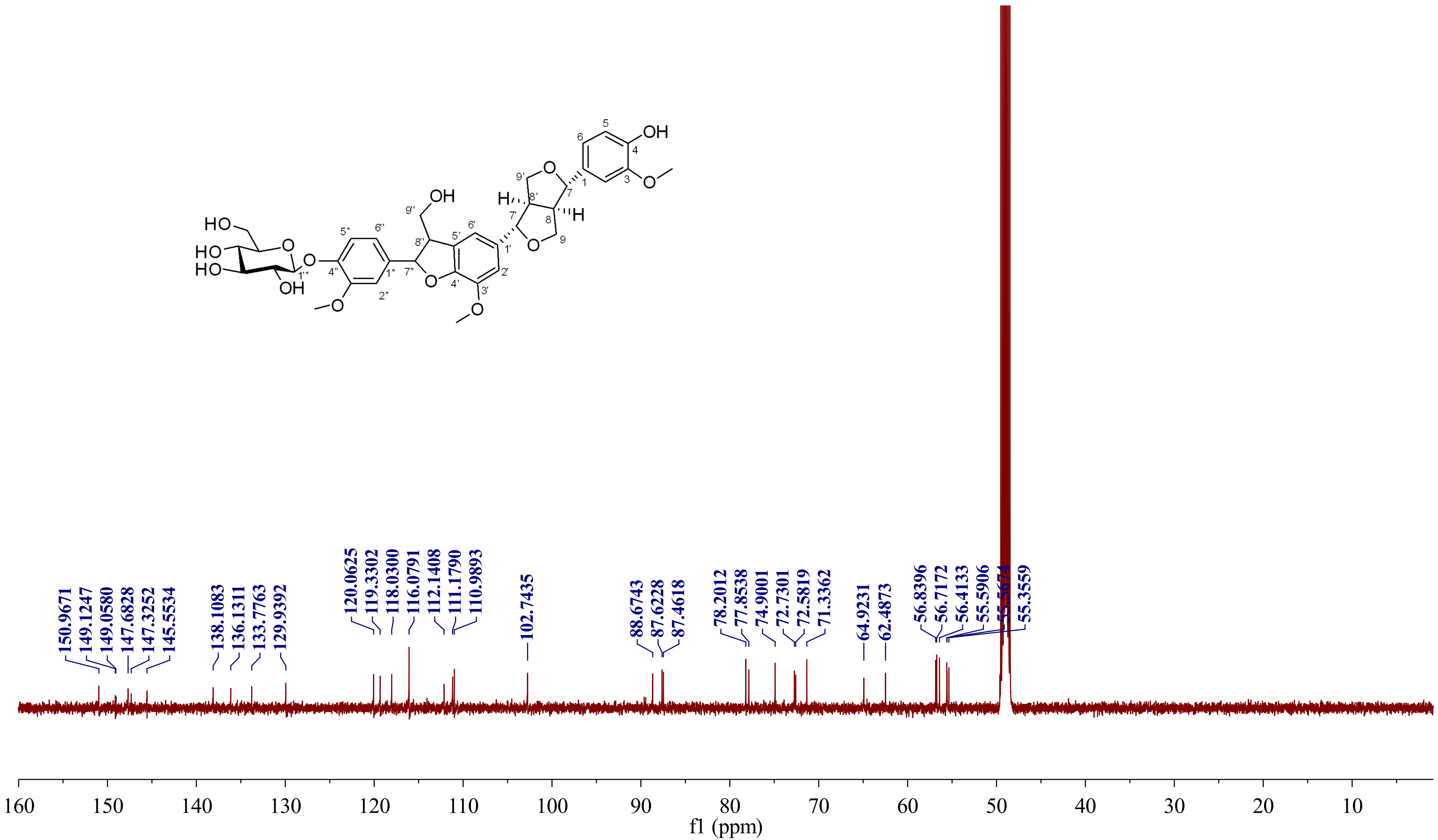

Figure S9. ^{13}**CNMR spectrum of compound 1 (CD$_3$OD, 125 MHz)**

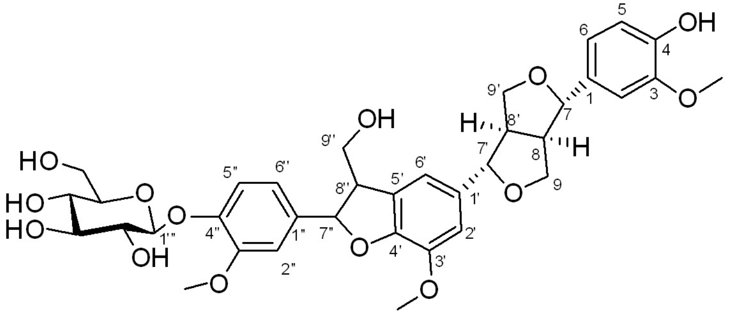

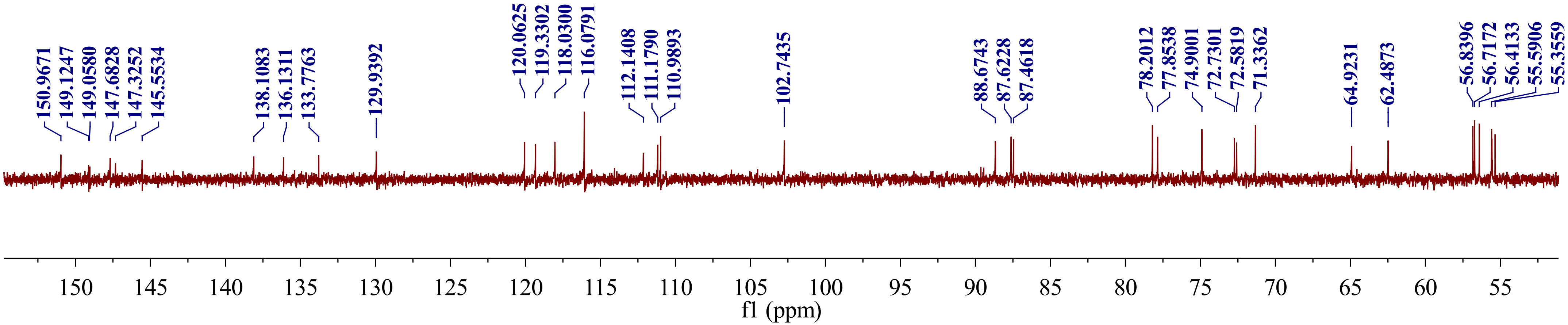

Figure S10. Expanded ^{13}CNMR spectrum of compound 1 (CD_3OD, 125 MHz)

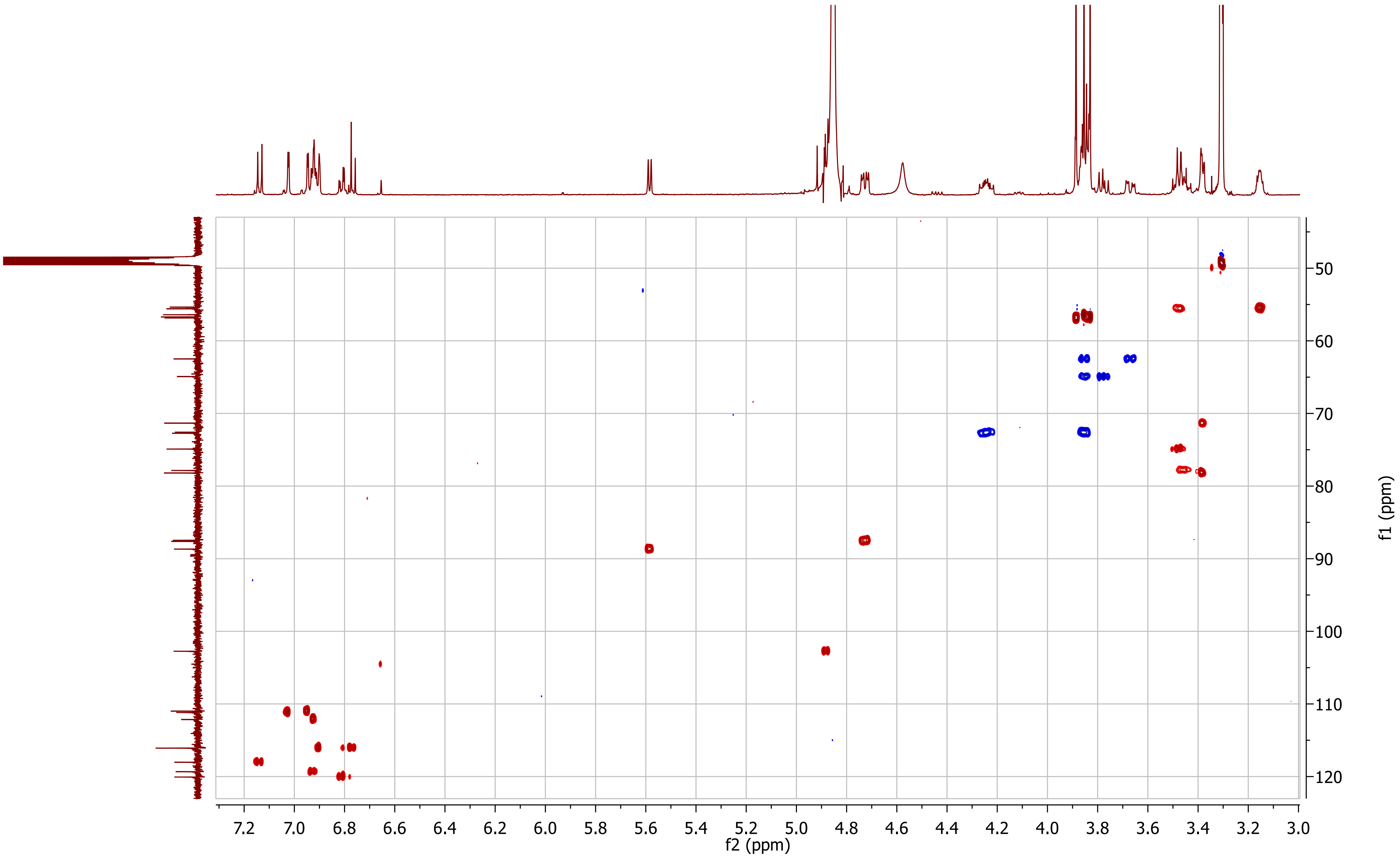

Figure S11. HSQC spectrum of compound 1

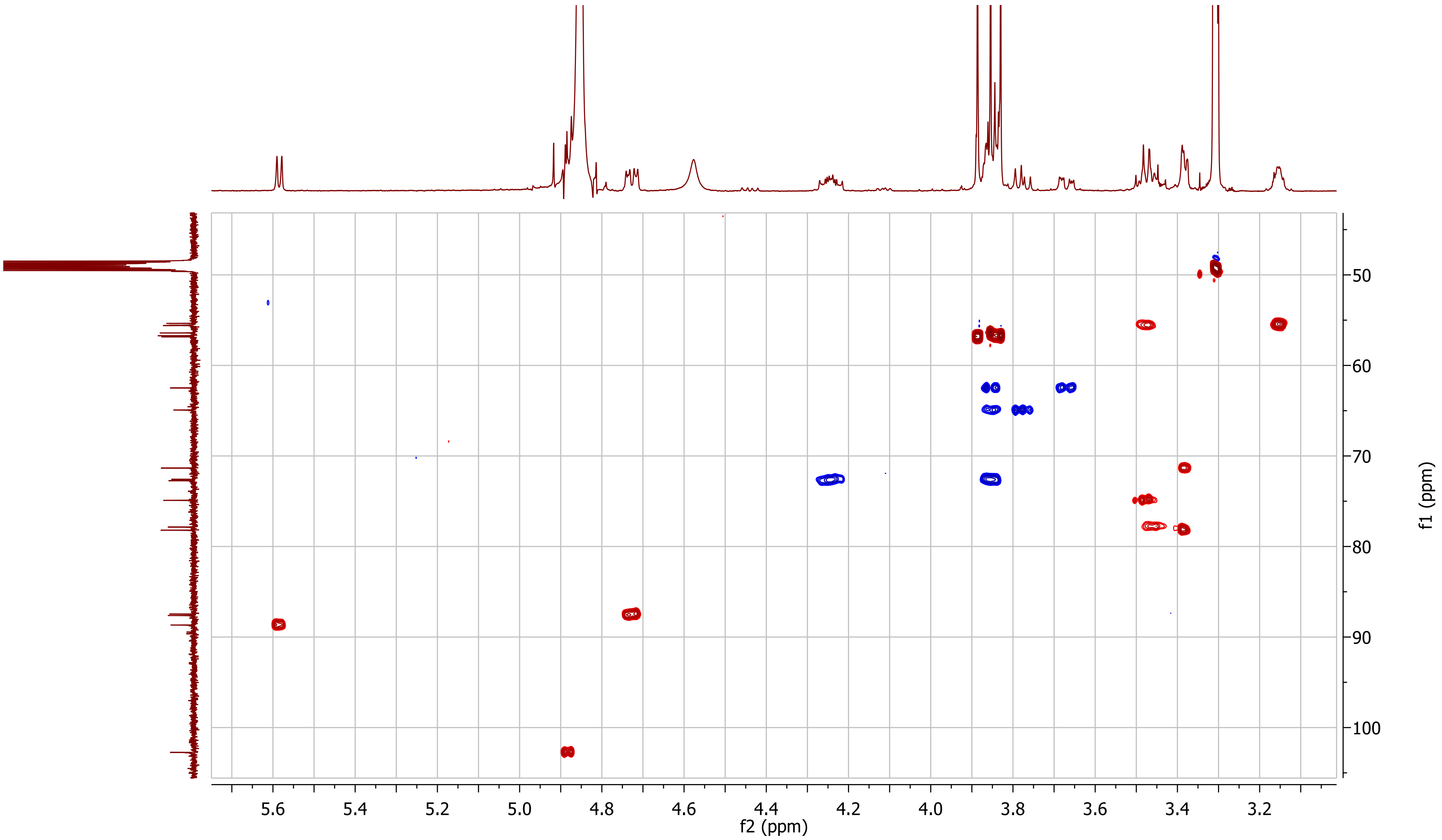

Figure S12. Expanded HSQC spectrum of compound 1

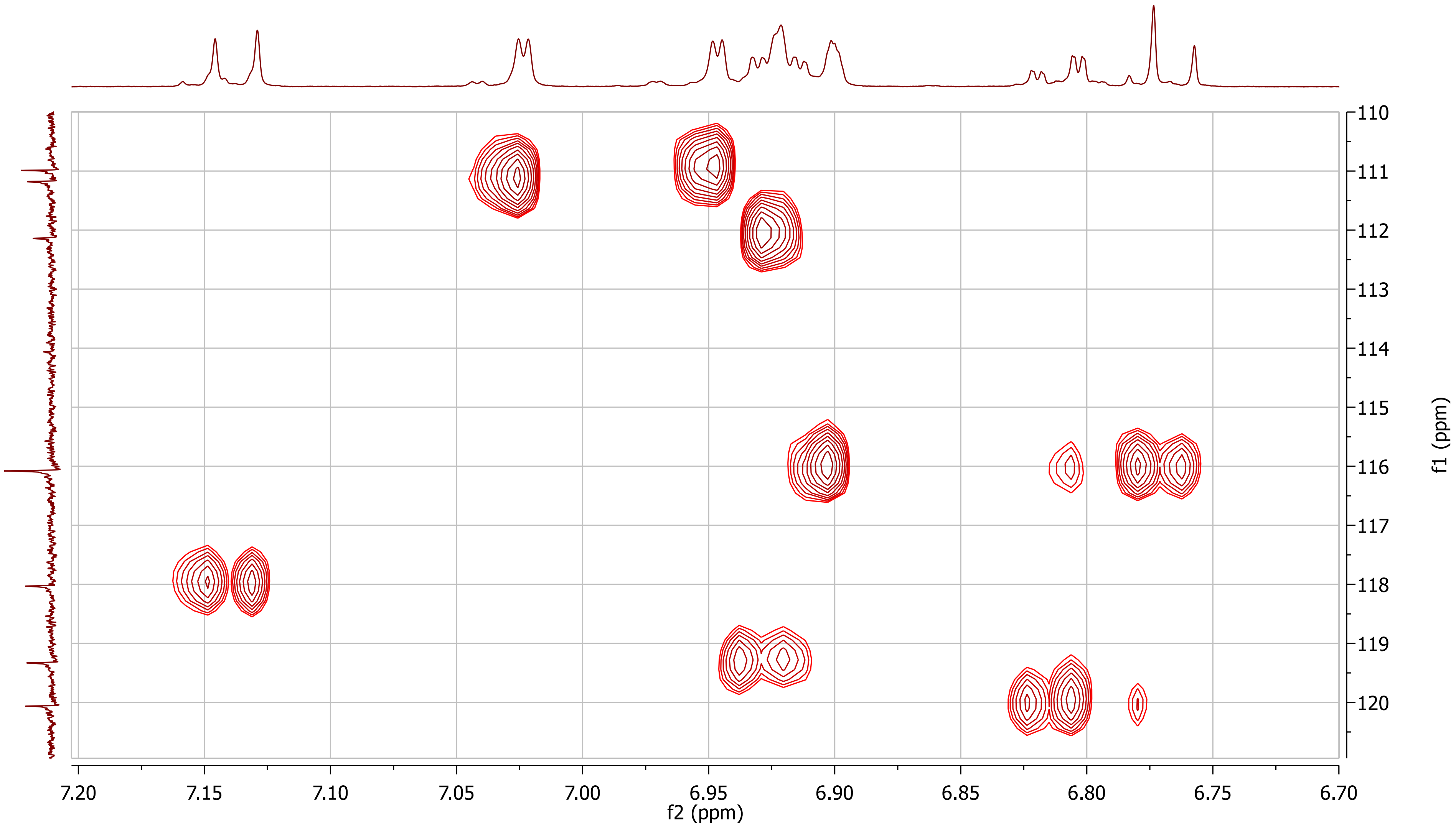

Figure S13. Expanded HSQC spectrum of compound 1

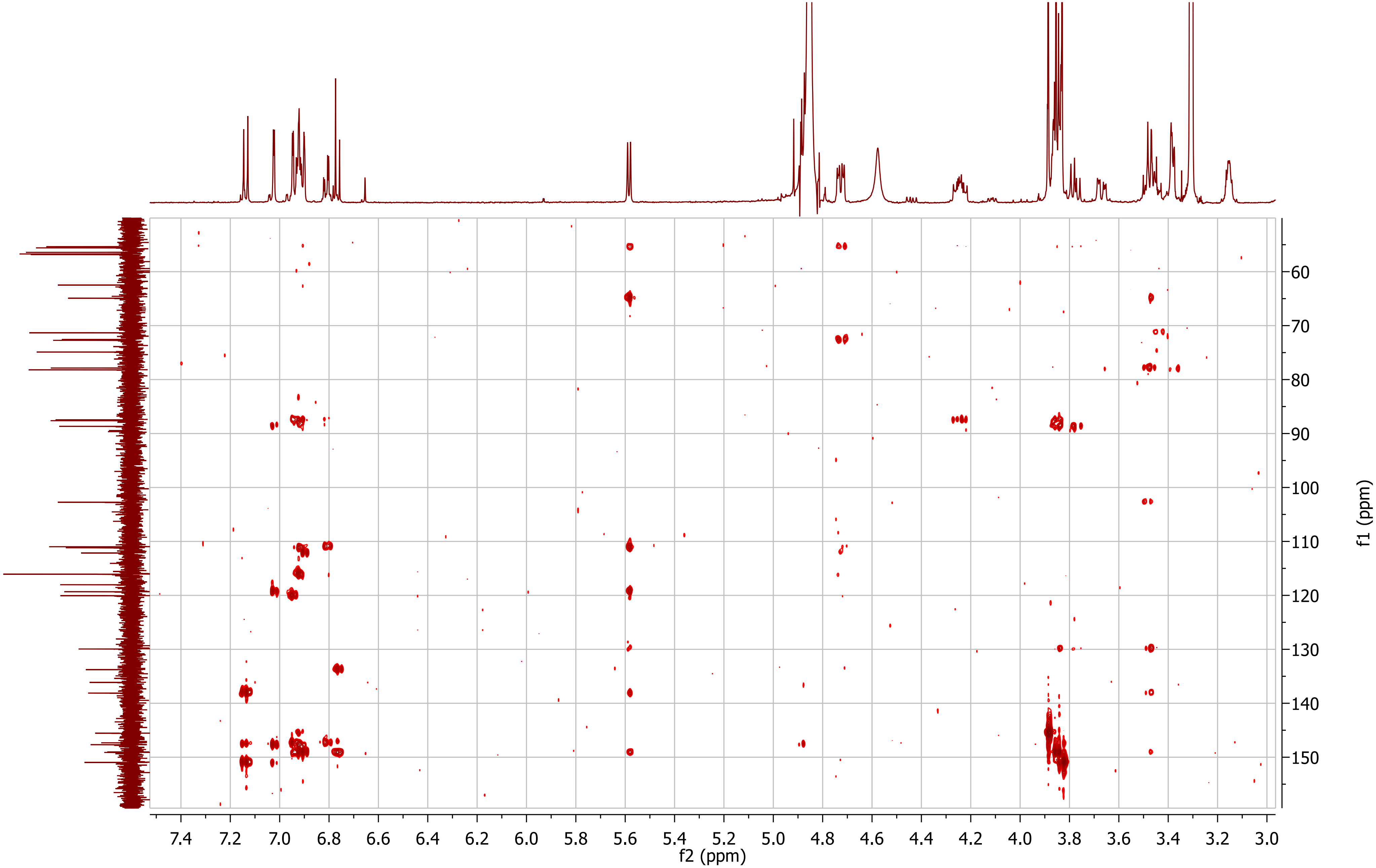

Figure S14. HMBC spectrum of compound 1

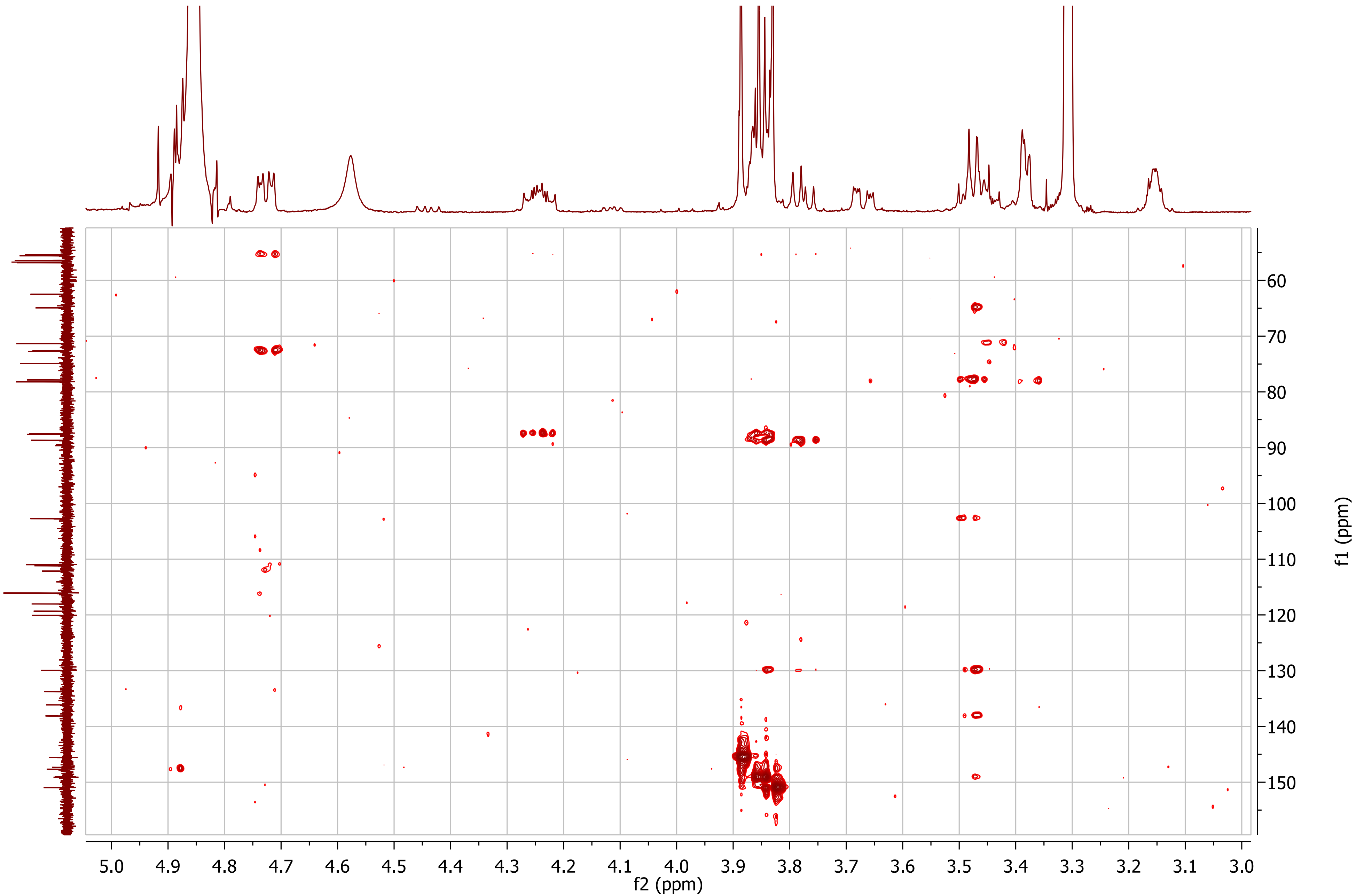

Figure S15. Expanded HMBC spectrum of compound 1

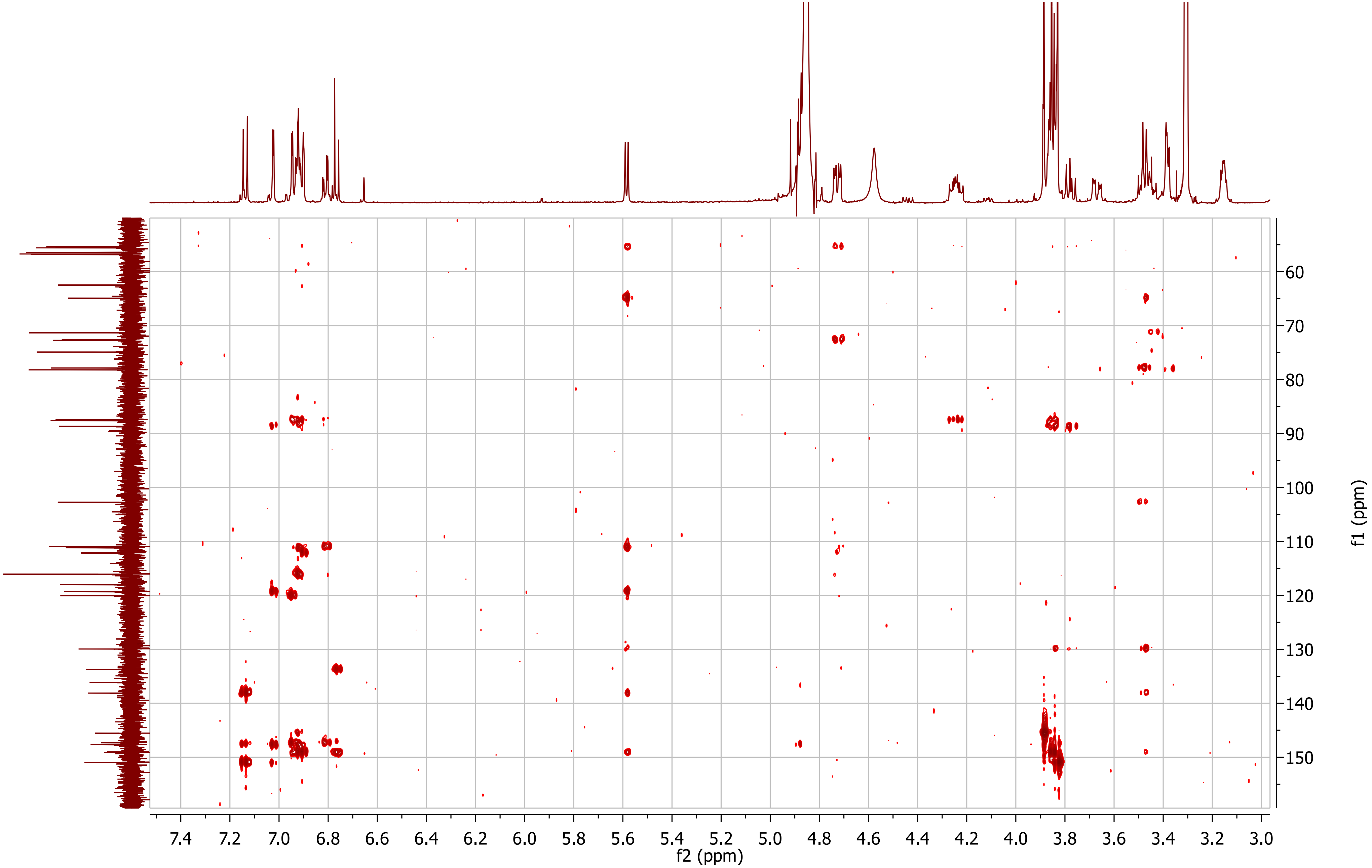

Figure S16. Expanded HMBC spectrum of compound 1

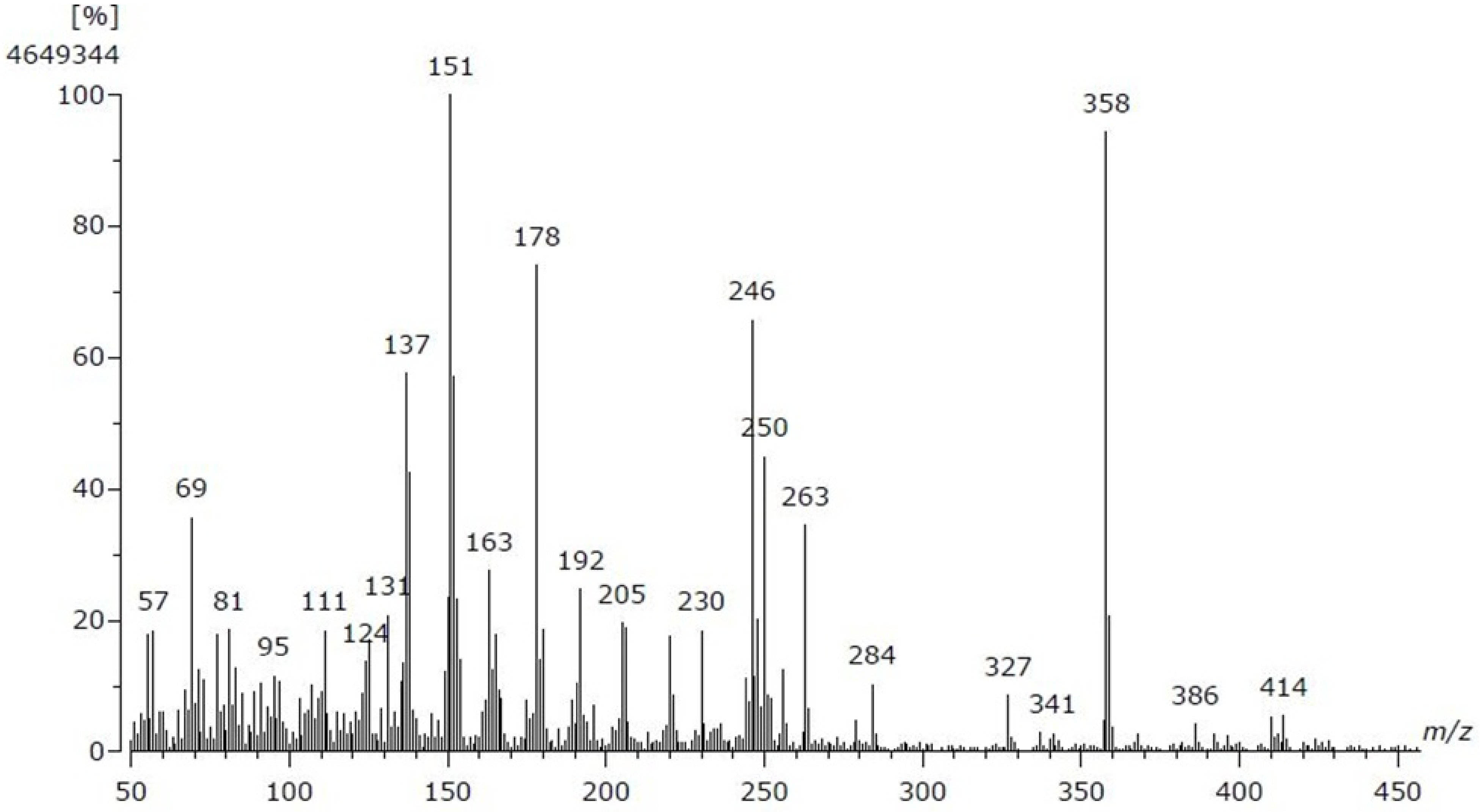

Figure S17. EI-MS spectrum of compound 2

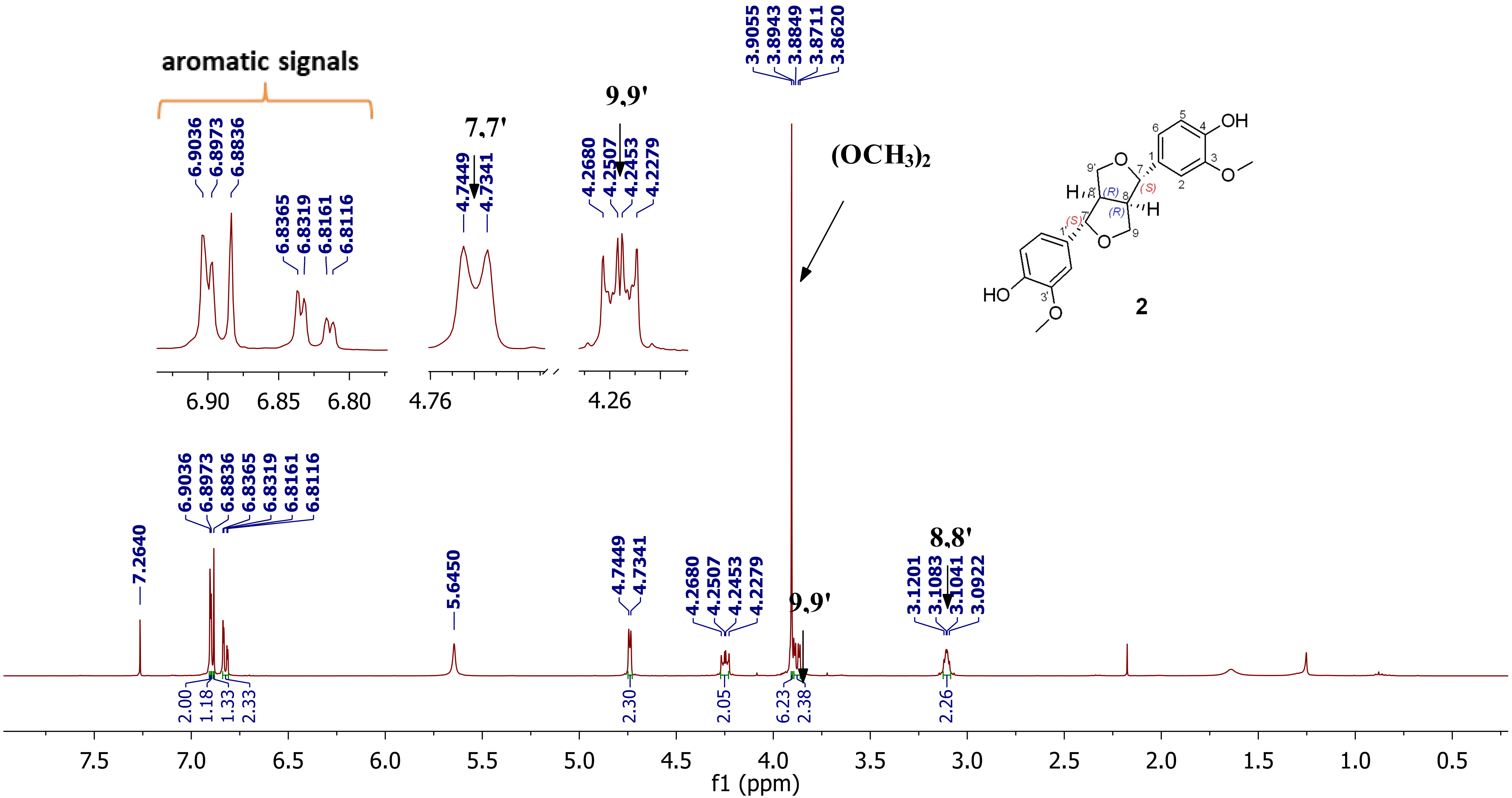

Figure S18. ^{1}HNMR spectrum of compound 2 (CD_3OD, 400 MHz)

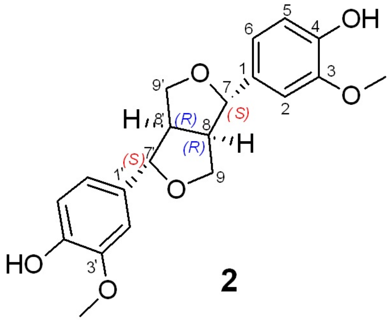

2

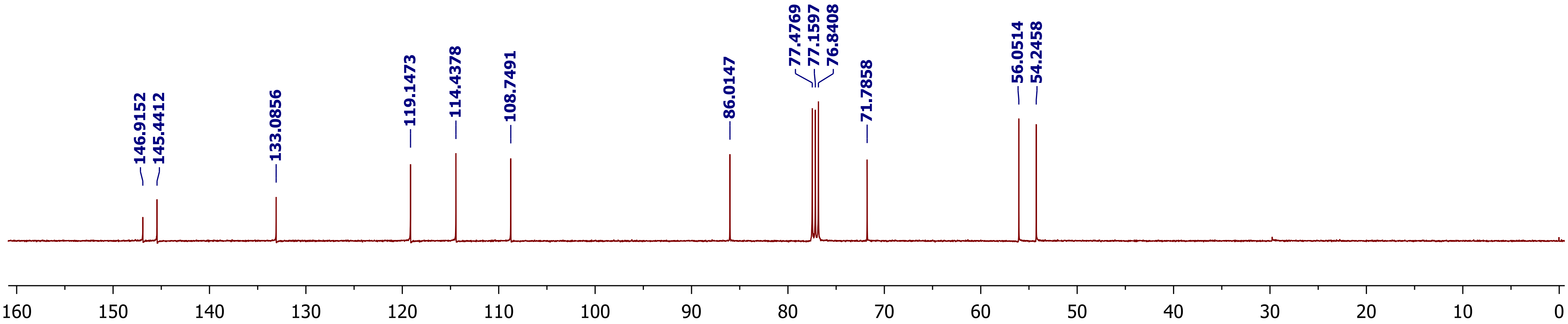

Figure S19. ^{13}CNMR spectrum of compound 2 (CD_3OD, 100 MHz)

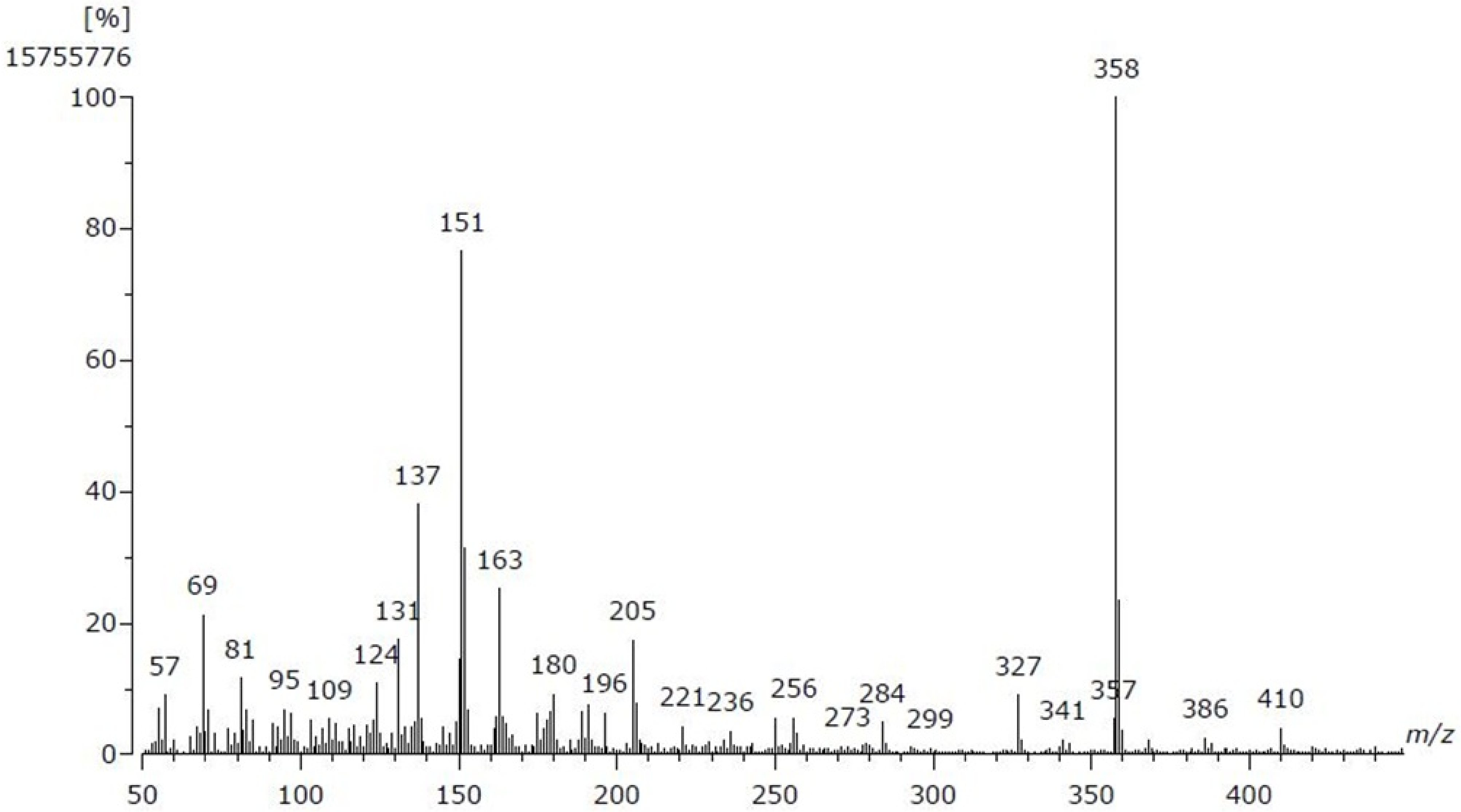

Figure S20. EI-MS spectrum of compound 3

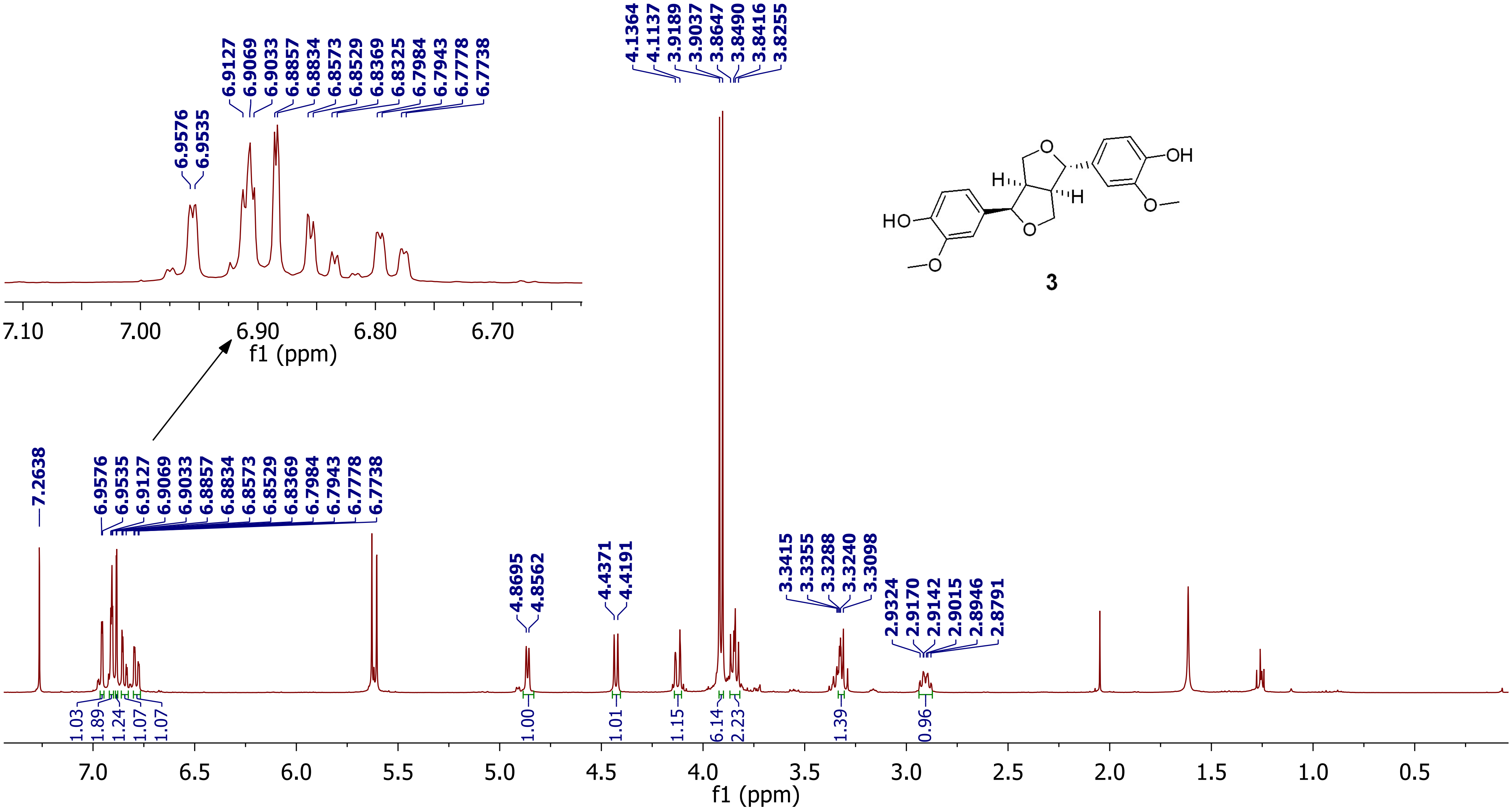

3

Figure S21. ^{1}**HNMR spectrum of compound 3 (CDCL$_3$, 100 MHz)**

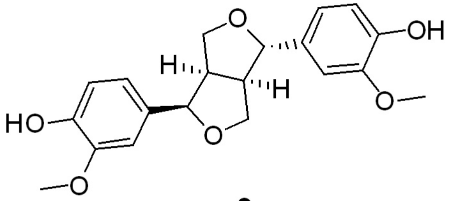

3

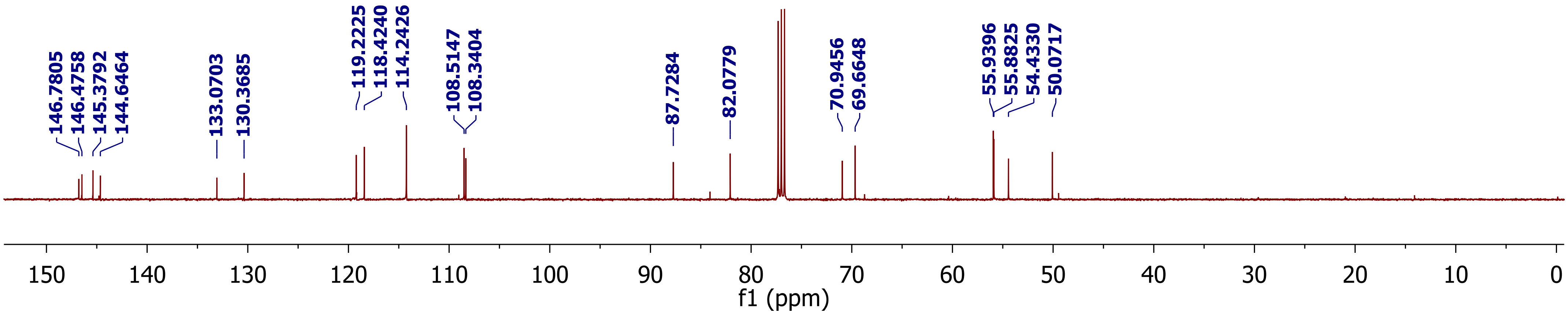

Figure S22. ^{13}**CNMR spectrum of compound 3 (CDCL$_3$, 100 MHz)**

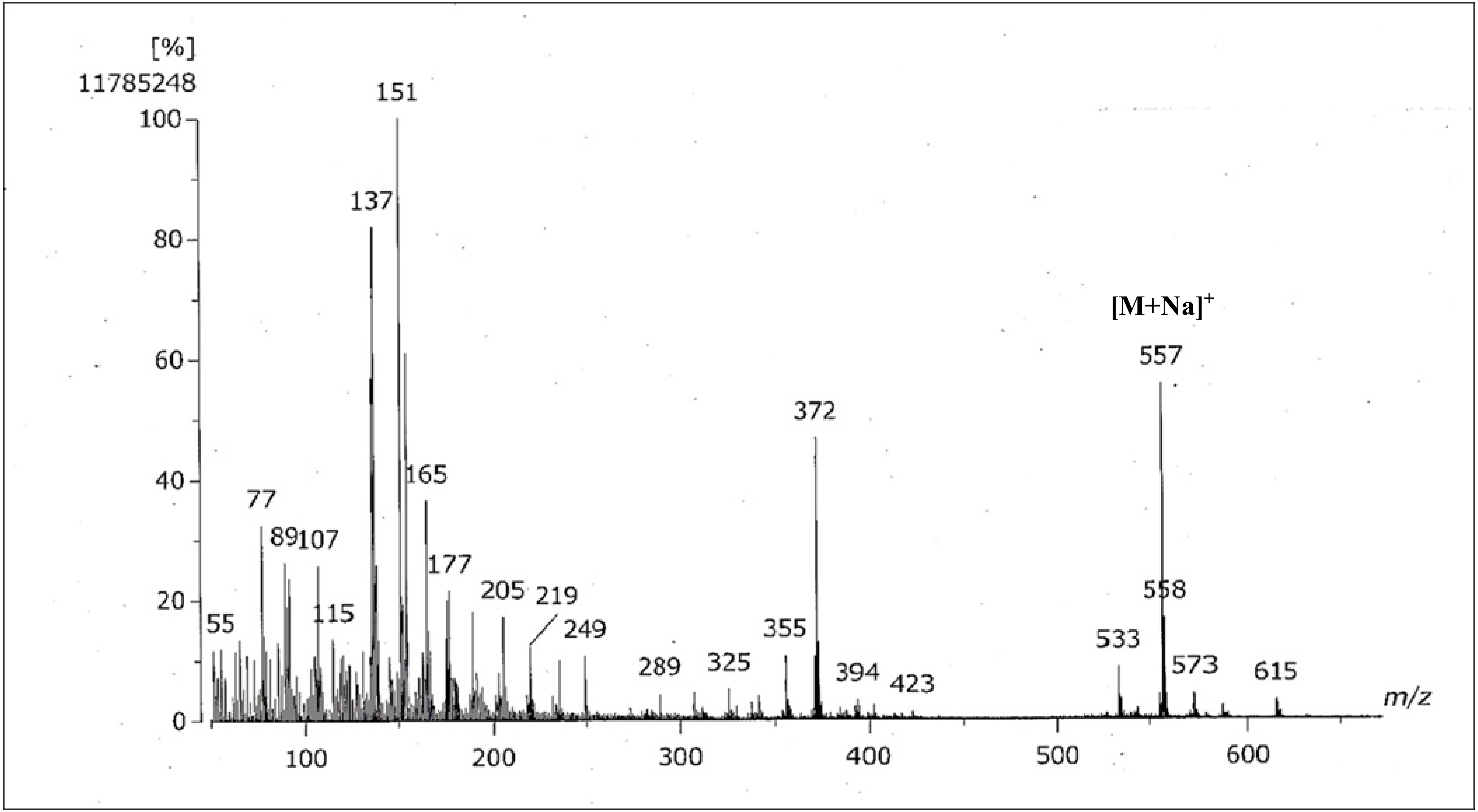

Figure S23. Positive FAB-MS spectrum of compound 4

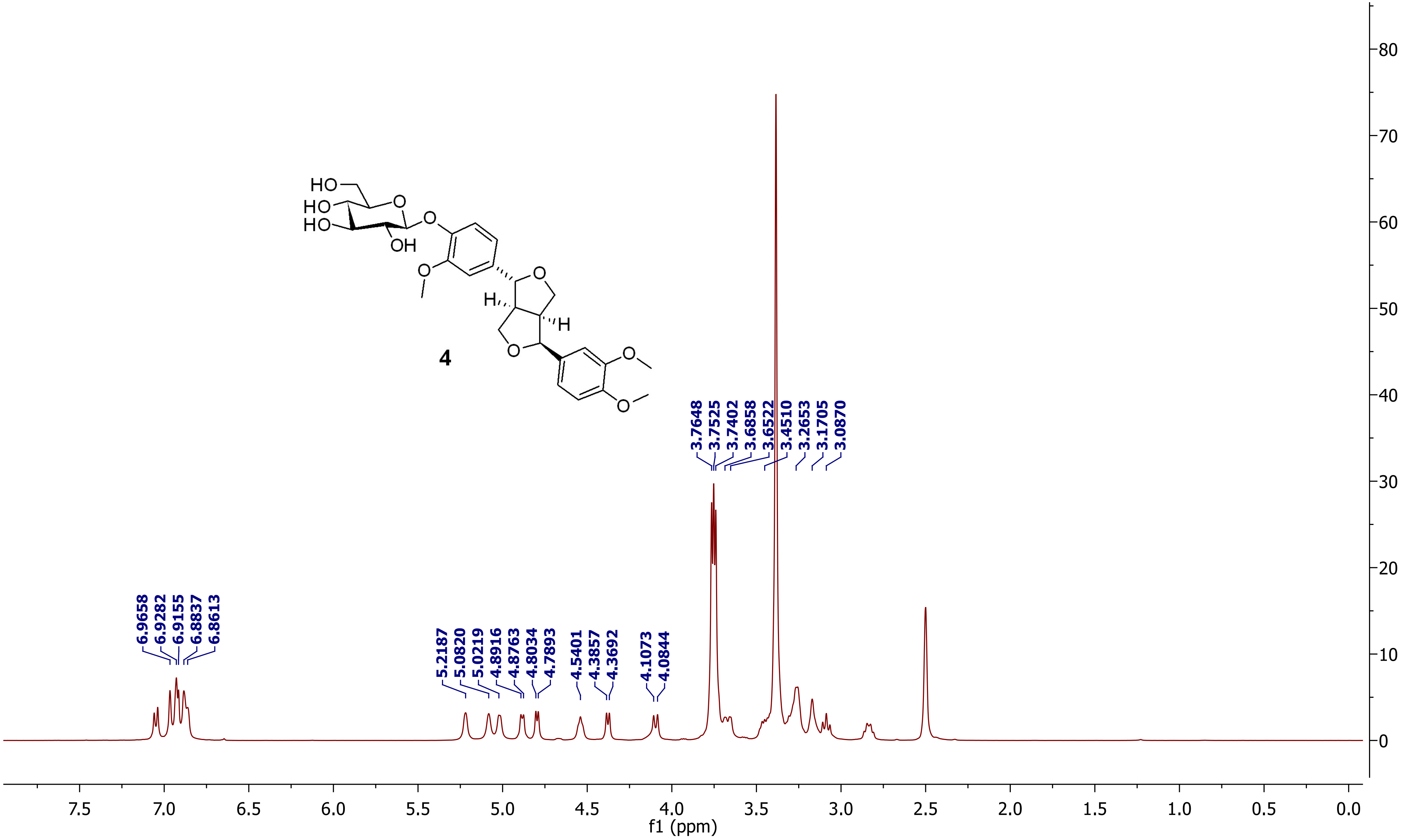

Figure S24. ^{1}HNMR spectrum of compound 4 (DMSO-d_6, 400 MHz)

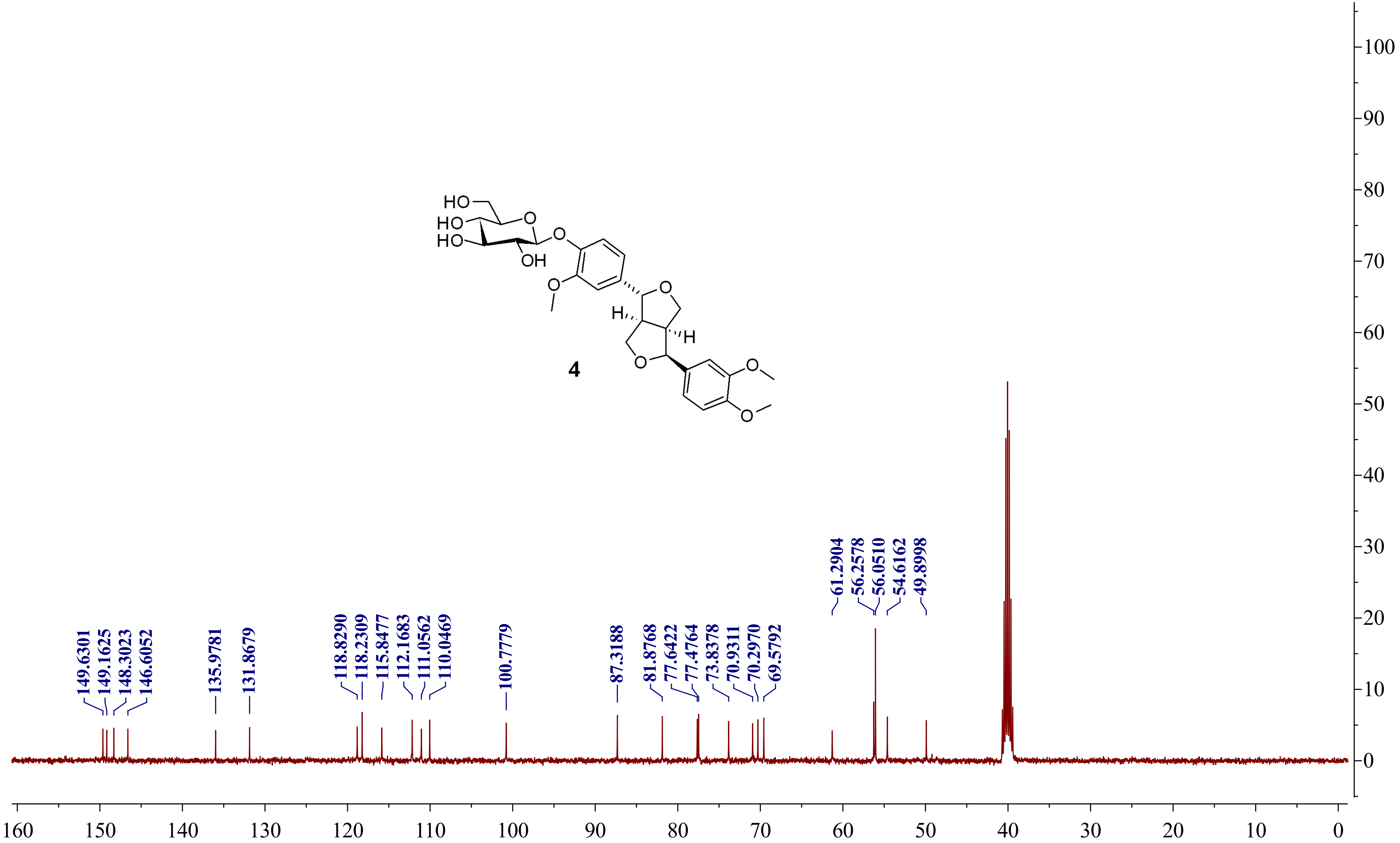

Figure S25. ^{13}**CNMR spectrum of compound 4 (DMSO-d_6, 100 MHz)**

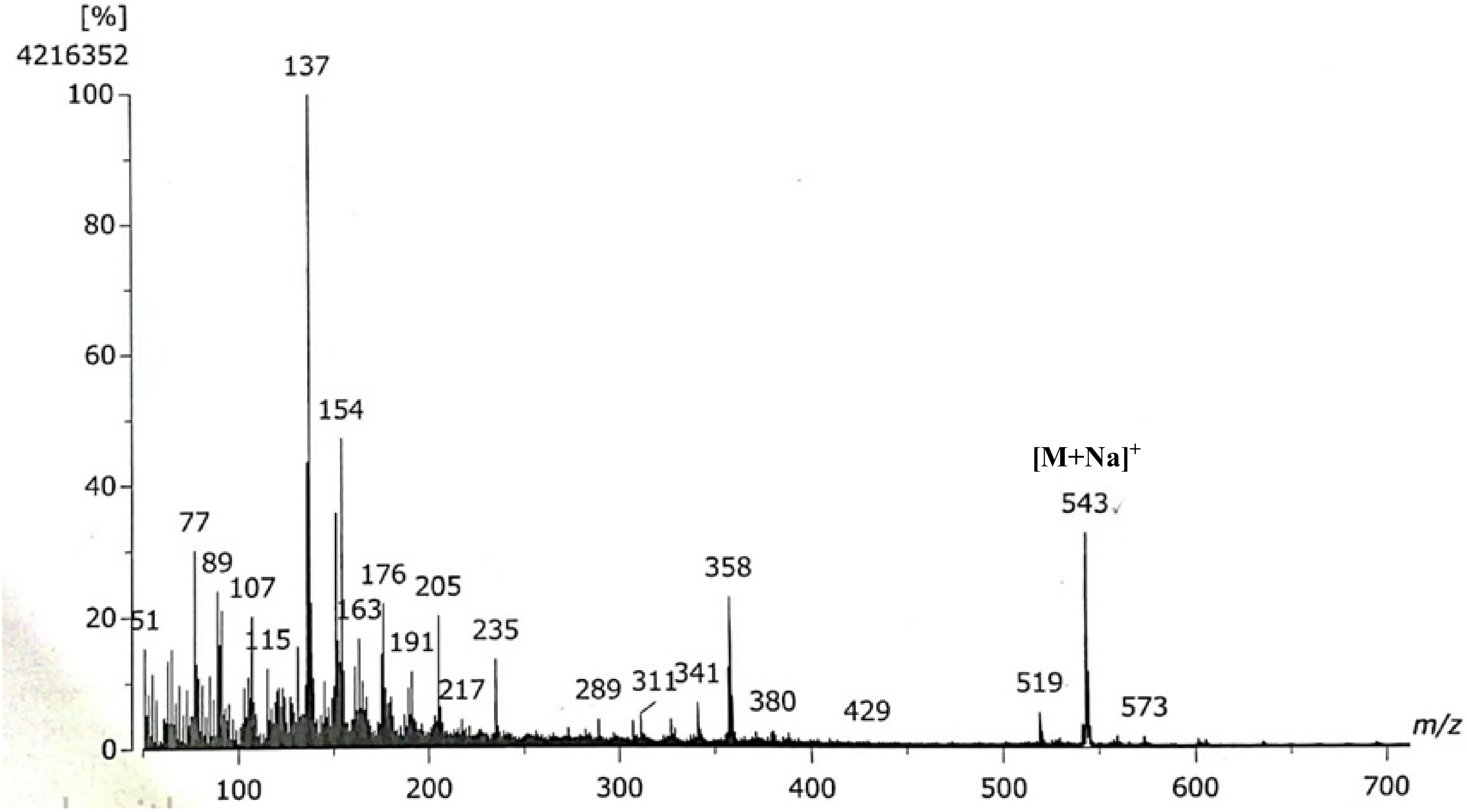

Figure S26. Positive FAB-MS spectrum of compound 5

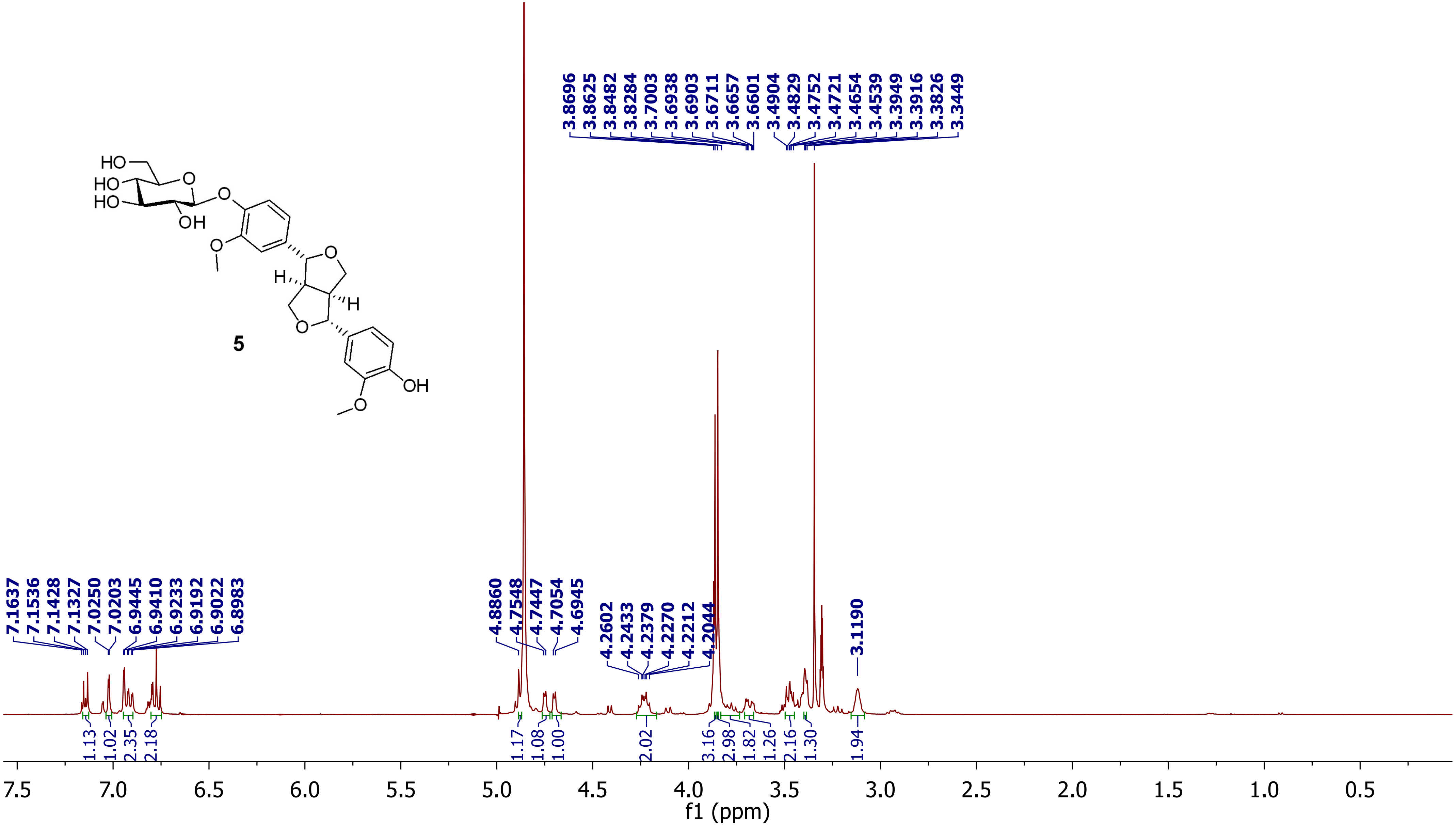

Figure S27. [1]**HNMR spectrum of compound 5 (CD$_3$OD, 400 MHz)**

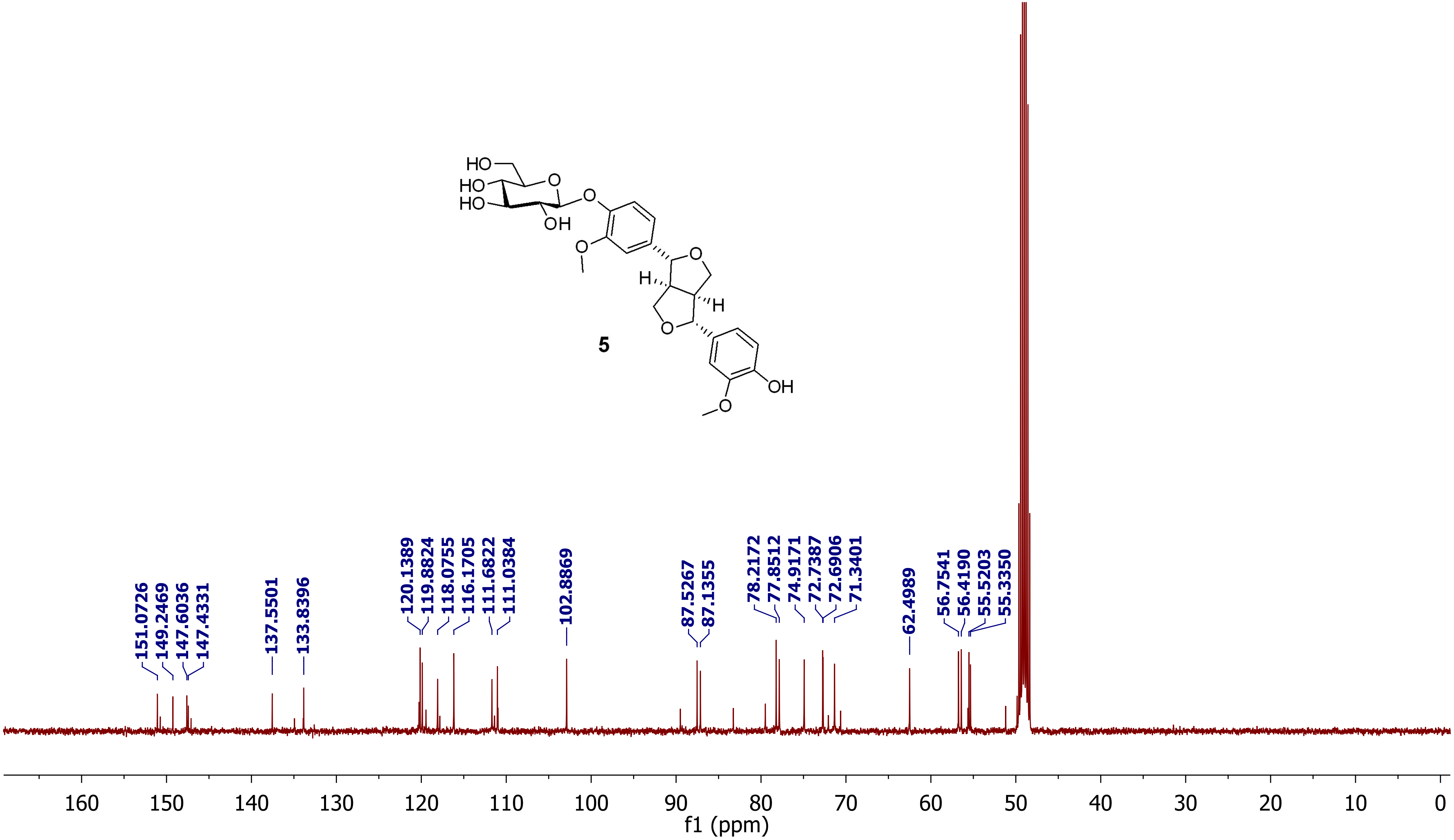

Figure S28. ^{13}CNMR spectrum of compound 5 (CD_3OD, 100 MHz)

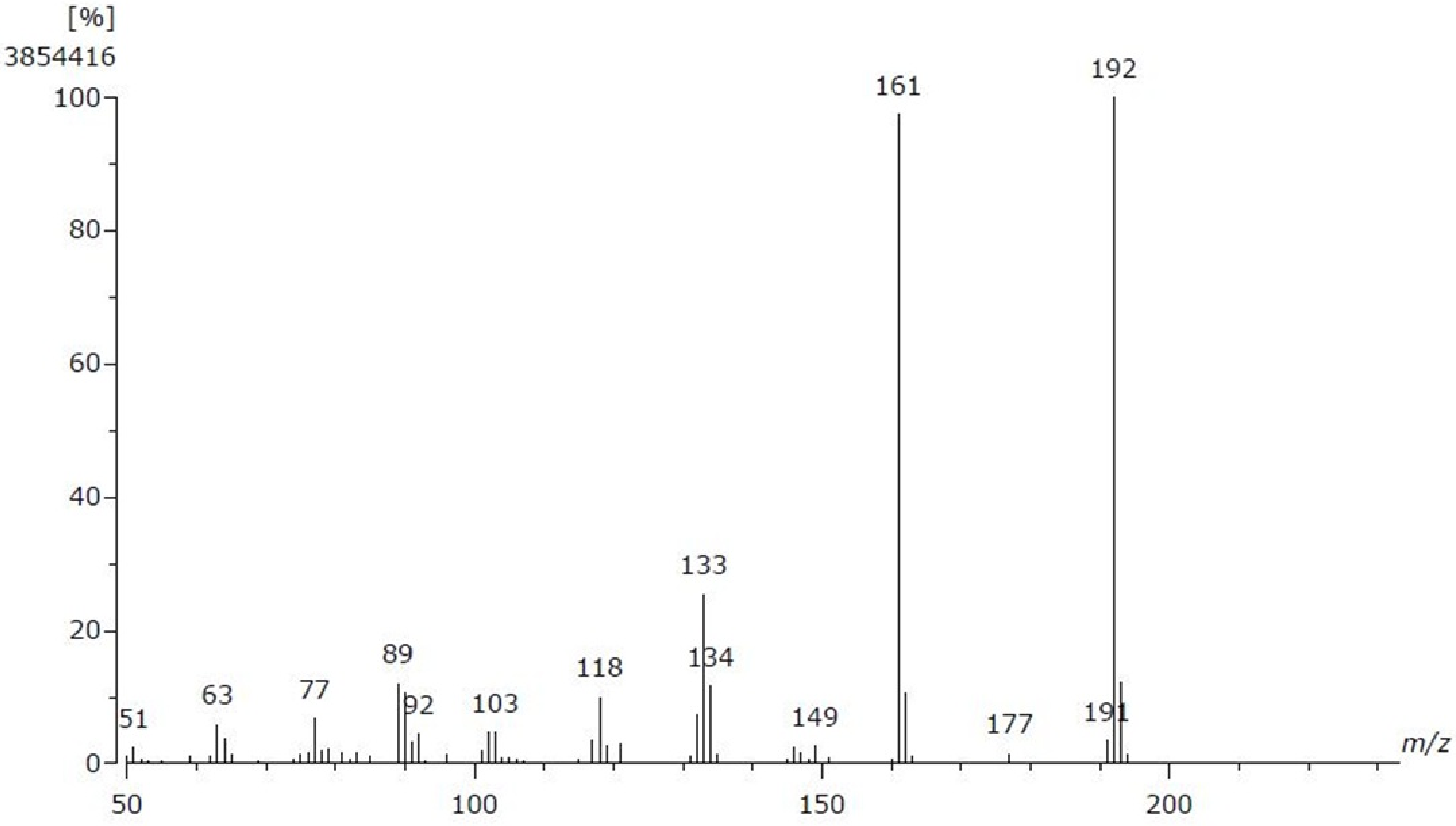

Figure S29. EI-MS spectrum of compound 6

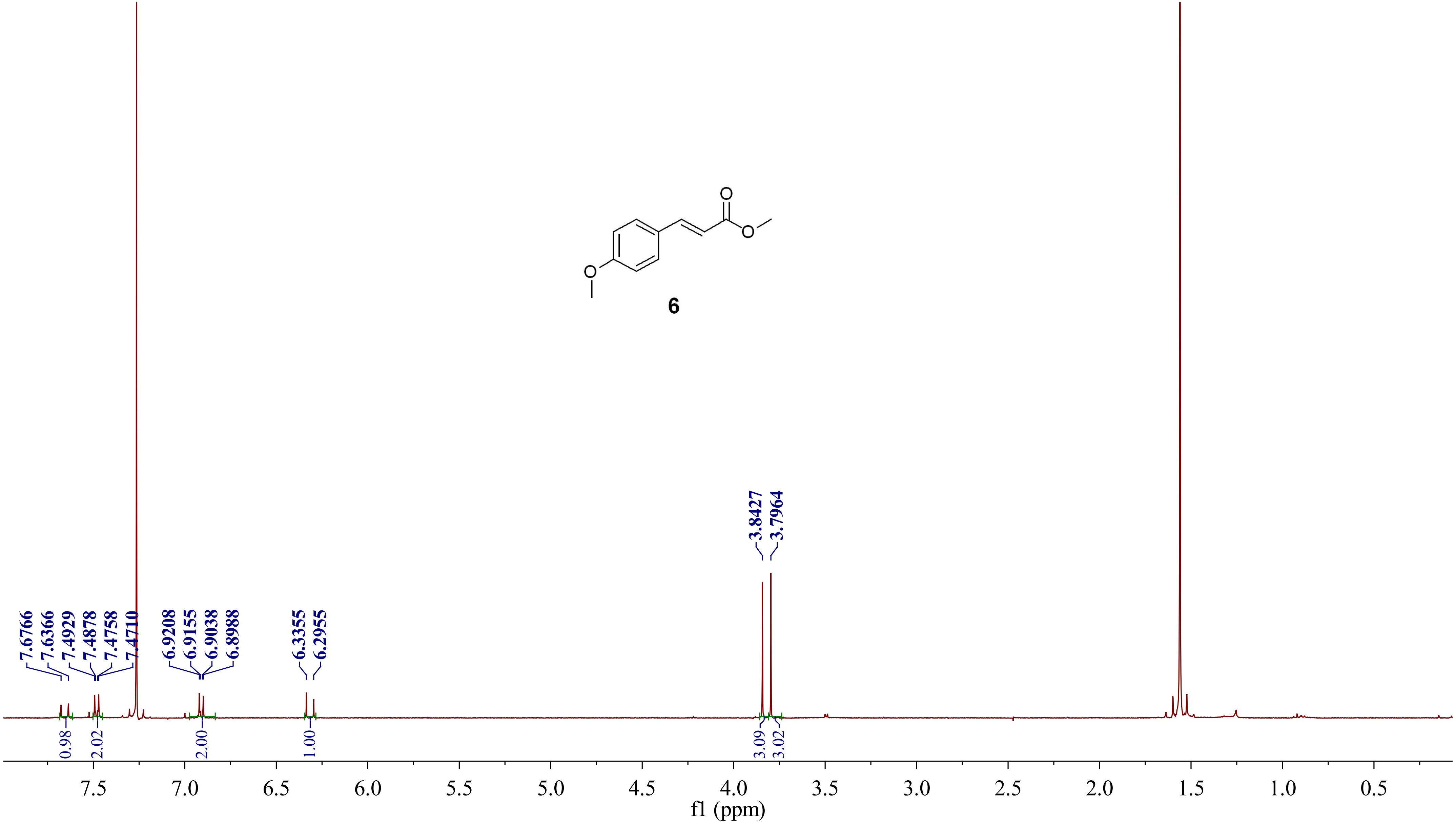

Figure S30. ^{1}HNMR spectrum of compound 6 ($CDCL_3$, 400 MHz)

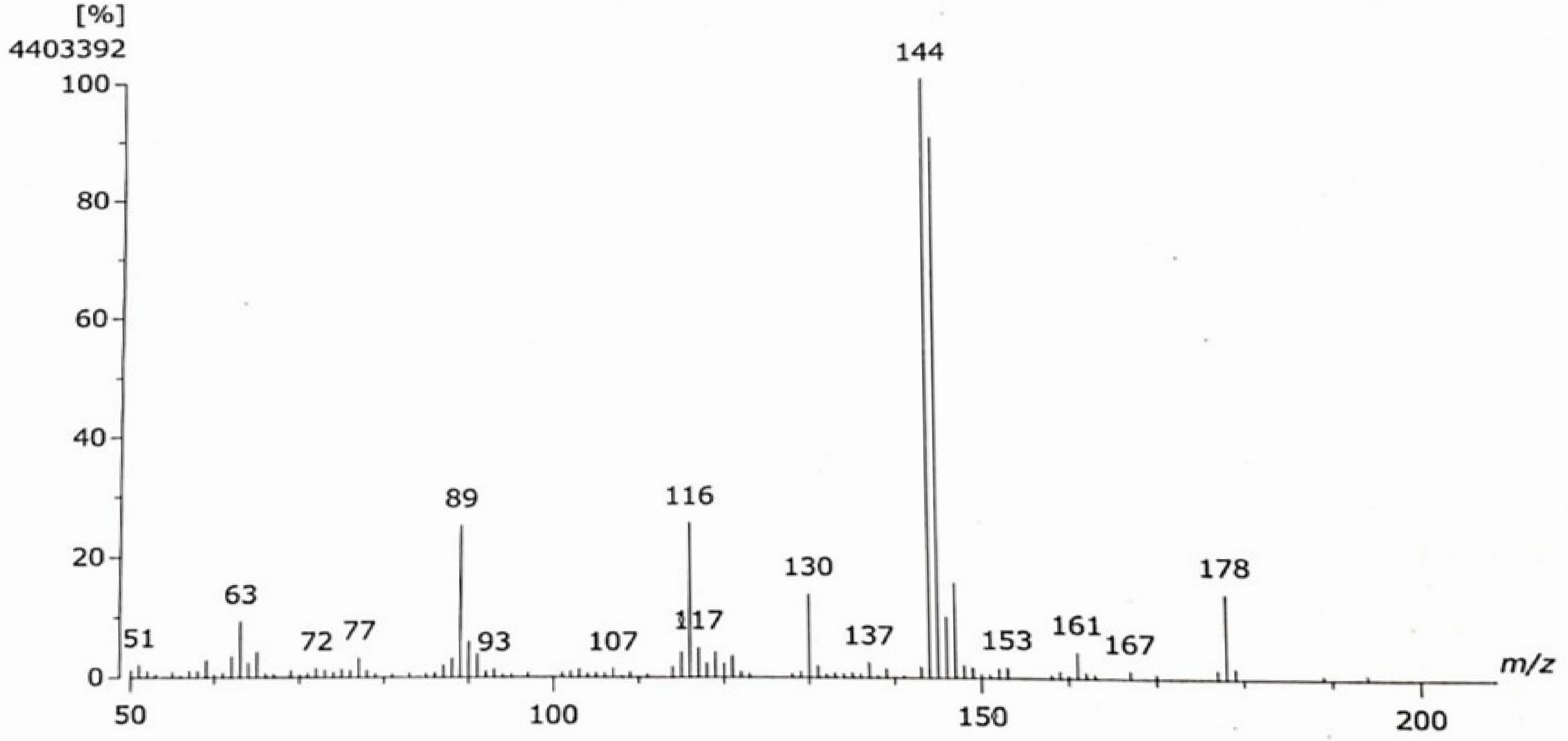

Figure S31. EI-MS spectrum of compound 7

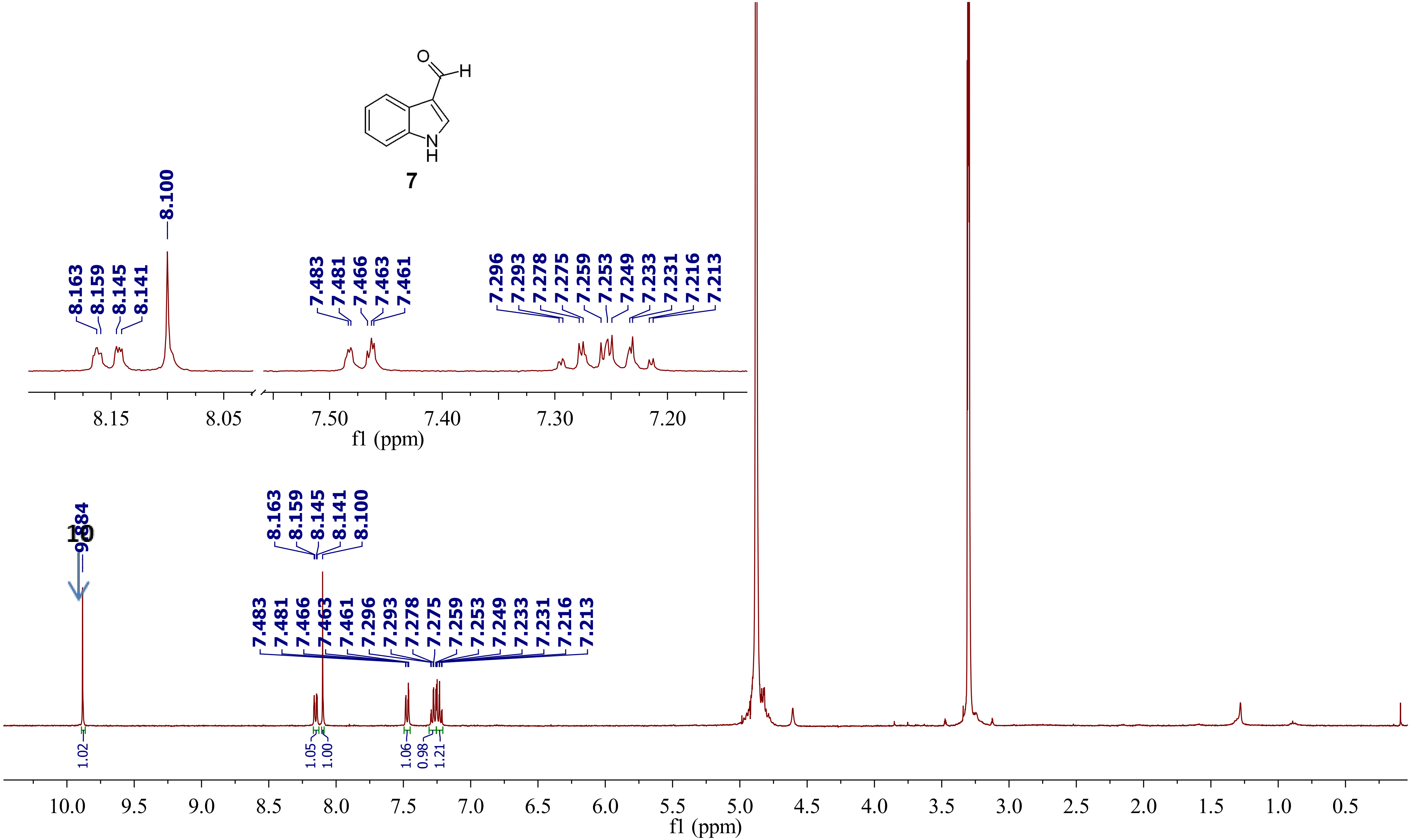

Figure S32. **^{1}HNMR spectrum of compound 7 (CD_3OD, 400 MHz)**

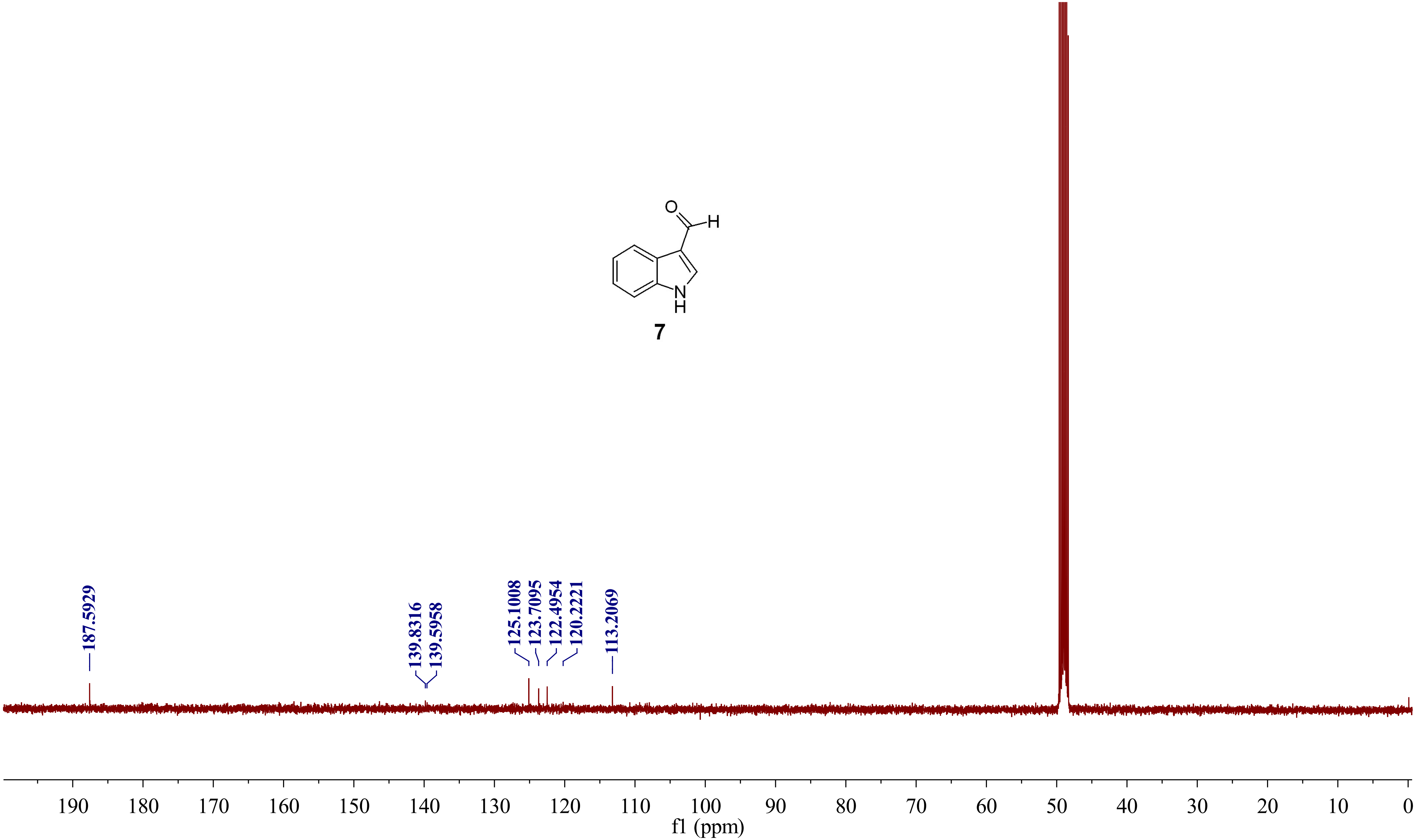

Figure S33. ^{13}**CNMR spectrum of compound 7 (CD$_3$OD, 100 MHz)**

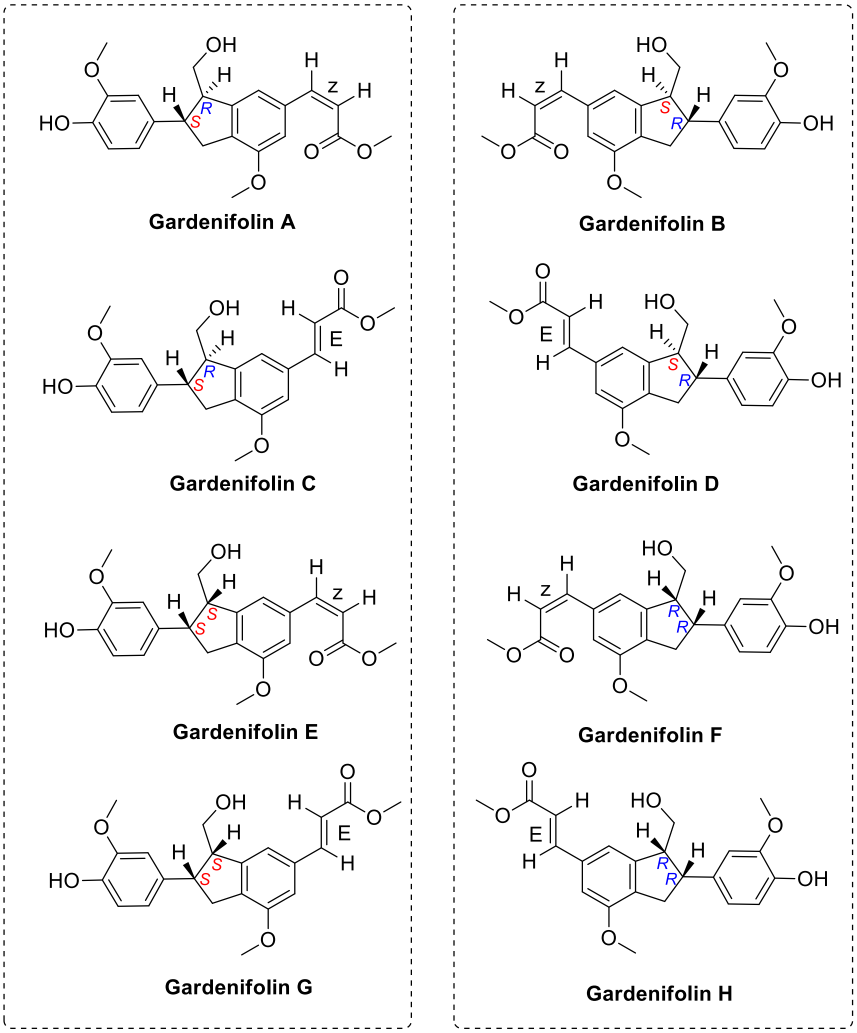

Figure S34. Structures of gardenifolins A, C, E, and G and their respective mirror images gardenifolins B, D, F, and H [S1].

Spectroscopic data of the isolated known compounds **2**–**7**.

- Pinoresinol (**2**): A colorless needles, ^{1}H NMR ($CDCl_3$, 400 MHz): δ_H 6.87 (2H, d, *J* = 2.5 Hz, H-2 and H-2′), 6.86 (2H, d, *J* = 8.1 Hz, H-5 and H-5′), 6.80 (2H, dd, *J* = 8.1, 1.8 Hz, H-6 and H-6′), 4.71 (2H, d, *J* = 4.3 Hz, H-7 and H-7′), 3.08 (2H, m, H-8 and H-8′), 4.22 (2H, dd, *J* = 9.1, 6.9 Hz, H_2-9 and H_2-9′), 3.86 (2H, d, *J* = 3.6, Hz, H_2-9 and H_2-9′), and 3.88 [6H, s, $(O\text{-}CH_3)_2$]. ^{13}C NMR ($CDCl_3$, 100 MHz): δ_C 133.0 (2C, C-1 and C-1′), 108.7 (2C, C-2, and C-2′), 146.9 (2S, C-3 and C-3′), 145.4 (2C, C-4 and C-4′), 114.4 (2C, C-5 and C-5′), 119.1 (2C, C-6 and C-6′), 86.0 (2C, C-7 and C-7′), 54.2 (2C, C-8 and C-8′), 71.7 (2C, C-9 and C-9′), and 56.0[2C, $(O\text{-}CH_3)_2$]. EI-MS *m*/*z* 358 $[M]^+$ [S2].

- Epipinoresinol (**3**): A colorless needles, ^{1}H NMR ($CDCl_3$, 400 MHz): δ_H 6.95 (1H, d, *J* = 1.6 Hz, H-2), 6.88 (1H, d, *J* = 8.4 Hz, H-5), 6.84 (1H, dd, *J* = 8.4, 1.6 Hz, H-6), 4.43 (1H, d, *J* = 7.2 Hz, H-7), 2.90 (1H, m, H-8), 4.12 (1H, d, *J* = 9.1 Hz, H_2-9), 3.83 (1H, d, *J* = 6.4, Hz, H_2-9), 6.90 (1H, d, *J* = 1.6 Hz, H-2′), 6.88 (1H, d, *J* = 8.4 Hz, H-5′), 6.78 (1H, dd, *J* = 8.2, 1.6 Hz, H-6′), 4.85 (1H, d, *J* = 5.3 Hz, H-7′), 3.32 (1H, m, H-8′), 3.33 (1H, m, H_2-9′), 3.85 (1H, d, *J* = 6.2 Hz, H_2-9′), 3.89 (3H, s, $O\text{-}CH_3$), and 3.91 (3H, s, $O\text{-}CH_3$). ^{13}C NMR ($CDCl_3$, 100 MHz): δ_C 133.0 (C-1), 108.5 (C-2), 146.7 (C-3), 145.3 (C-4), 114.2 (C-5), 119.2 (C-6), 87.7 (C-7), 54.4 (C-8), 70.9 (C-9), 130.3 (C-1′), 108.3 (C-2′), 146.4 (C-3′), 144.6 (C-4′), 114.2 (C-5′), 118.4 (C-6′), 82.0 (C-7′), 50.0 (C-8′), 69.6 (C-9′) 55.8 ($O\text{-}CH_3$), and 55.9 ($O\text{-}CH_3$). EI-MS *m*/*z* 358 $[M]^+$ [3].

- Phillyrin (**4**): A yellowish white powder, ^{1}H NMR (DMSO-d_6, 400 MHz): δ_H 6.96 (^{1}H, brs, H-2), 7.05 (1H, d, *J* = 8.5 Hz, H-5), 6.90 (1H, br.d, *J* = 8.5, Hz, H-6), 4.37 (1H, d, *J* = 6.6 Hz, H-7), 2.83 (1H, dd, *J* = 14.7, 6.7 Hz, H-8), 4.09 (1H, d, *J* = 9.1 Hz, H_2-9), 3.79 (1H, m, H_2-9), 6.92 (1H, brs, H-2′), 6.87 (1H, d, *J* = 8.5 Hz, H-5′), 6.90 (1H, brd, *J* = 8.5 Hz, H-6′), 4.79 (1H, d, *J* = 5.7 Hz, H-7′), 3.30 (1H, m, H-8′), 3.35 (1H, m, H_2-9′), 3.79 (1H, m, H_2-9′), 4.88 (1H, d, *J* = 6.1 Hz, H-1″), 3.25 (2H, brs, H-2″, H-3″), 3.05 (1H, brs, H-4″), 3.06 (1H, t, *J* = 8.5 Hz, H-5″), 3.45 (1H, d, *J* = 5.9 Hz, H_2-6″), 3.65 (1H, m, H_2-6″), 3.74 (3H, s, $O\text{-}CH_3$), 3.75 (3H, s, $O\text{-}CH_3$), and 3.76 (3H, s, $O\text{-}CH_3$). ^{13}C NMR (DMSO-d_6, 100 MHz): δ_C 135.9 (C-1), 111.0 (C-2), 149.6 (C-3), 146.6 (C-4), 115.8 (C-5), 118.8 (C-6), 87.3 (C-7), 54.6 (C-8), 70.9 (C-9), 131.8 (C-1′), 110.0 (C-2′), 149.1 (C-3′), 148.3 (C-4′), 112.1 (C-5′), 118.2 (C-6′), 81.8 (C-7′), 49.8 (C-8′), 69.5 (C-9′), 100.7 (C-1″), 73.8 (C-2″), 77.4 (C-3″), 70.2

(C-4″), 77.6 (C-5″), 61.2 (C-6″), 56.0 [2C, (O-CH_3)$_2$], and 56.2 (O-CH_3). Positive FABMS *m/z* 557 $[M+Na]^+$ [4].

- Pinoresinol-4-*O*-β-D-glucoside (**5**): A yellowish residue, ^{1}H NMR (CD_3OD, 400 MHz): δ_H 7.02 (1H, d, *J* = 1.8 Hz, H-2), 7.13 (1H, d, *J* = 8.4 Hz, H-5), 6.91 (1H, dd, *J* = 8.4, 1.6 Hz, H-6), 4.74 (1H, d, *J* = 4.4 Hz, H-7), 3.19 (1H, m, H-8), 3.84 (1H, H_2-9), 4.22 (1H, dd, *J* = 9.0, 6.7 Hz, H_2-9), 6.94 (1H, d, *J* = 1.6 Hz, H-2′), 6.75 (1H, d, *J* = 8.5 Hz, H-5′), 6.79 (1H, dd, *J* = 8.5, 1.6 Hz, H-6′), 4.69 (1H, d, *J* = 4.3 Hz, H-7′), 3.11 (1H, m, H-8′), 3.84 (1H, m, H_2-9′), 4.22 (1H, dd, *J* = 9.0, 6.7 Hz H_2-9′), 4.88 (1H, d, *J* = 7.4 Hz, H-1″), 3.47 (1H, m, H-2″) 3.46(1H, m, H-3″), 3.39 (2H, m, H-4″, H-5″), 3.68 (1H, dd, *J* = 12.0, 5.3 Hz, H_2-6″), 3.85 (1H, m, H_2-6″), 3.86 (3H, s, O-CH_3), and 3.84 (3H, s, O-CH_3). ^{13}C NMR (CD_3OD, 100 MHz): δ_C 137.5 (C-1), 111.6 (C-2), 147.6 (C-3), 151.0 (C-4), 118.0 (C-5), 119.8 (C-6), 87.1 (C-7), 55.5 (C-8), 72.6 (C-9), 133.8 (C-1′), 111.0 (C-2′), 147.4 (C-3′), 149.2 (C-4′), 116.1 (C-5′), 120.1 (C-6′), 87.5 (C-7′), 55.3 (C-8′), 72.7 (C-9′), 102.8 (C-1″), 74.9 (C-2″), 77.8 (C-3″), 71.3 (C-4″), 78.2 (C-5″), 62.4 (C-6″), 56.4 (O-CH_3), and 56.7 (O-CH_3). Positive FABMS *m/z* 543 $[M+Na]^+$ [5].

- *p*-Methoxy-*trans*-methyl cinnamate (**6**): A white powder, ^{1}H NMR ($CDCl_3$, 400 MHz): δ_H 7.48 (2H, d, *J* = 8.8 Hz, H-2/H-6), 6.91 (2H, d, *J* = 8.8 Hz, H-3/H-5), 7.65 (1H, d, *J* = 16.0 Hz, H-7), 6.31(1H, d, *J* = 16.0 Hz, H-8), 3.49 (3H, s, O-CH_3), and 3.84 (3H, s, O-CH_3). EI-MS *m/z* 192 $[M]^+$ [6].

- 1H-indole-3-carboxaldehyde (**7**): A white powder, ^{1}H NMR (DMSO-d_6, 400 MHz): δ_H 8.10 (1H, s, H-2), 8.15 (1H, brd, J = 7.2 Hz, H-4), 7.28 (1H, ddd, J = 8.8, 7.2, 1.2 Hz, H-5), 7.23 (1H, ddd, J = 8.0, 7.2, 1.2 Hz, H-6), 7.47 (1H, brd, J = 7.2 Hz, H-7), and 9.88 (1H, H-10). EI-MS m/z 145 $[M]^+$ and positive FABMS m/z 146 $[M+H]^+$ [7,8].

code	IC50	conc.uM	log conc	%inh	T2	T1	ΔT	RFU2	RFU1	ΔRFU	slope	K.Activity	EC
Compound 1		10	4	82.67538	30	0	30	0.136	0	0.136	0.026167	20.78954	120
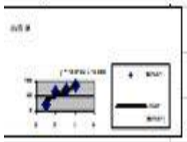		1	3	74.77739	30	0	30	0.198	0	0.198	0.026167	30.26713	120
		0.1	2	60.89222	30	0	30	0.307	0	0.307	0.026167	46.92934	120
		0.01	1	24.58695	30	0	30	0.592	0	0.592	0.026167	90.49566	120
EC				0	30	0	30	0.785	0	0.785	0.026167	120	120

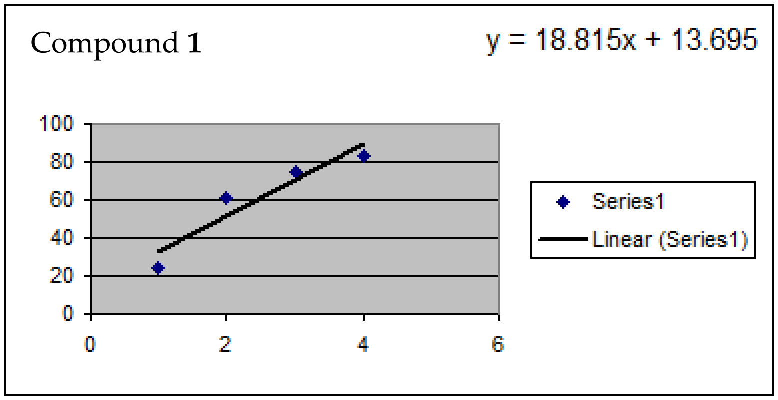

Figure S35. Dose-response curve of the anti-AChE assay of compound **1**.

code	IC50	conc.uM	log conc	%inh	T2	T1	ΔT	RFU2	RFU1	ΔRFU	slope	K.Activity	EC
Compound 2		10	4	75.54171	30	0	30	0.192	0	0.192	0.026167	29.34994	120
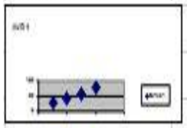		1	3	60.00051	30	0	30	0.314	0	0.314	0.026167	47.99939	120
		0.1	2	40.76509	30	0	30	0.465	0	0.465	0.026167	71.0819	120
		0.01	1	25.9882	30	0	30	0.581	0	0.581	0.026167	88.81416	120
EC				0	30	0	30	0.785	0	0.785	0.026167	120	120

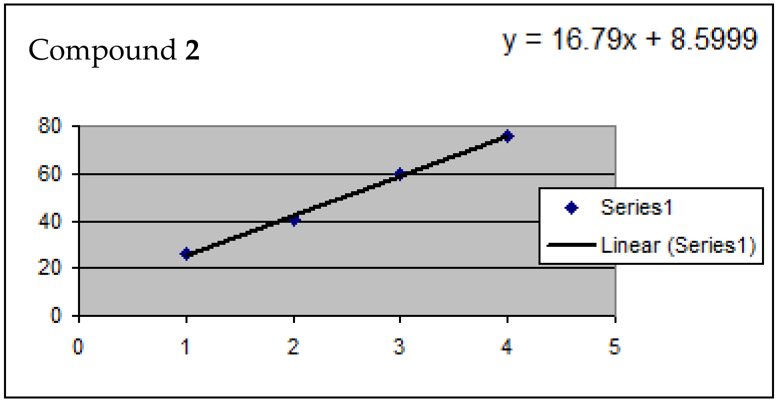

Figure S36. Dose-response curve of the anti-AChE assay of compound **2**.

code	IC50	conc.uM	log conc	%inh	T2	T1	ΔT	RFU2	RFU1	ΔRFU	slope	K.Activity	EC
Compound 3		10	4	81.78367	30	0	30	0.143	0	0.143	0.026167	21.85959	120
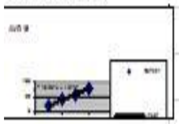		1	3	60.38267	30	0	30	0.311	0	0.311	0.026167	47.5408	120
		0.1	2	43.5676	30	0	30	0.443	0	0.443	0.026167	67.71888	120
		0.01	1	23.18569	30	0	30	0.603	0	0.603	0.026167	92.17717	120
EC				0	30	0	30	0.785	0	0.785	0.026167	120	120

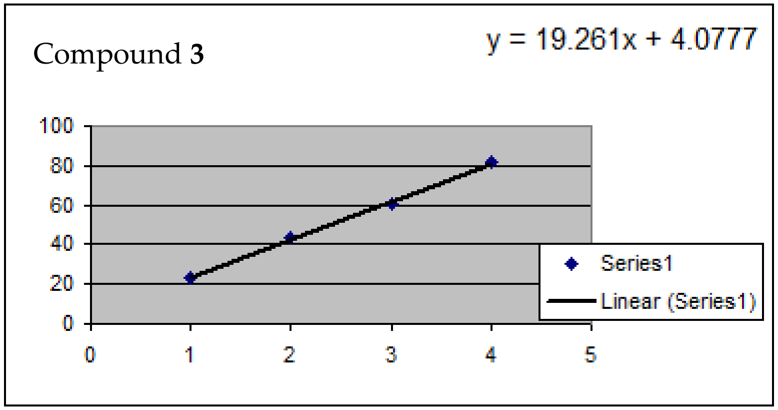

Figure S37. Dose-response curve of the anti-AChE assay of compound **3**.

code	IC50	conc.uM	log conc	%inh	T2	T1	ΔT	RFU2	RFU1	ΔRFU	slope	K.Activity	EC
Compound 4		10	4	72.86659	30	0	30	0.213	0	0.213	0.026167	32.56009	120
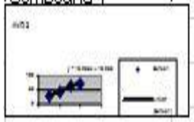		1	3	63.69473	30	0	30	0.285	0	0.285	0.026167	43.56632	120
		0.1	2	40.6377	30	0	30	0.466	0	0.466	0.026167	71.23476	120
		0.01	1	26.37036	30	0	30	0.578	0	0.578	0.026167	88.35556	120
EC				0	30	0	30	0.785	0	0.785	0.026167	120	120

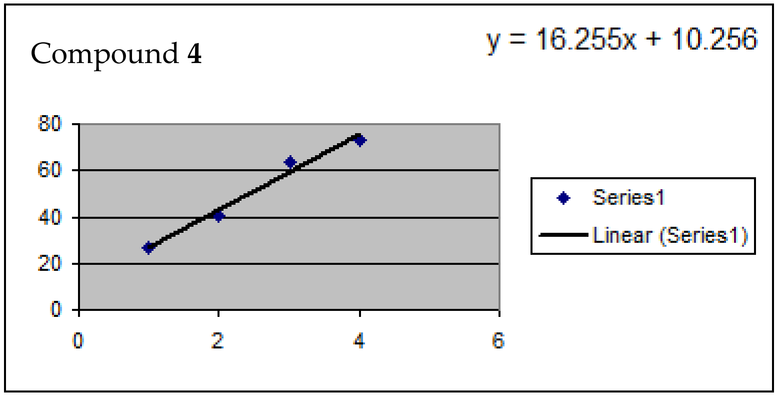

Figure S38. Dose-response curve of the anti-AChE assay of compound **4**.

code	IC50	conc.uM	log conc	%inh	T2	T1	ΔT	RFU2	RFU1	ΔRFU	slope	K.Activity	EC
Compound 5		10	4	87.77086	30	0	30	0.096	0	0.096	0.026167	14.67497	120
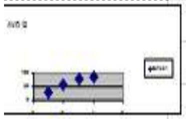		1	3	78.85377	30	0	30	0.166	0	0.166	0.026167	25.37547	120
		0.1	2	59.87312	30	0	30	0.315	0	0.315	0.026167	48.15225	120
		0.01	1	28.15378	30	0	30	0.564	0	0.564	0.026167	86.21546	120
EC				0	30	0	30	0.785	0	0.785	0.026167	120	120

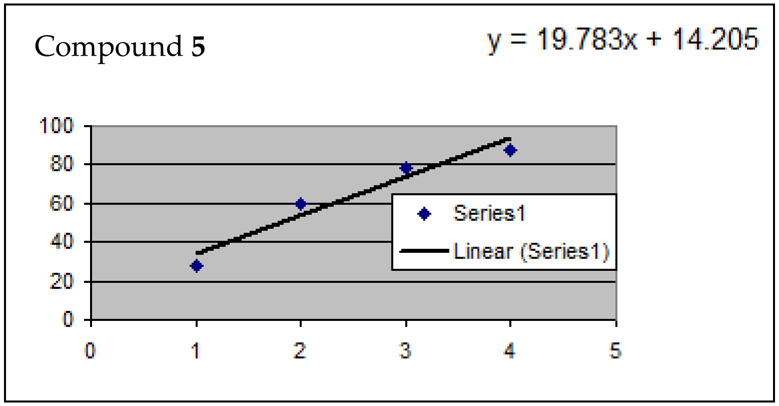

Figure S39. Dose-response curve of the anti-AChE assay of compound **5**.

A

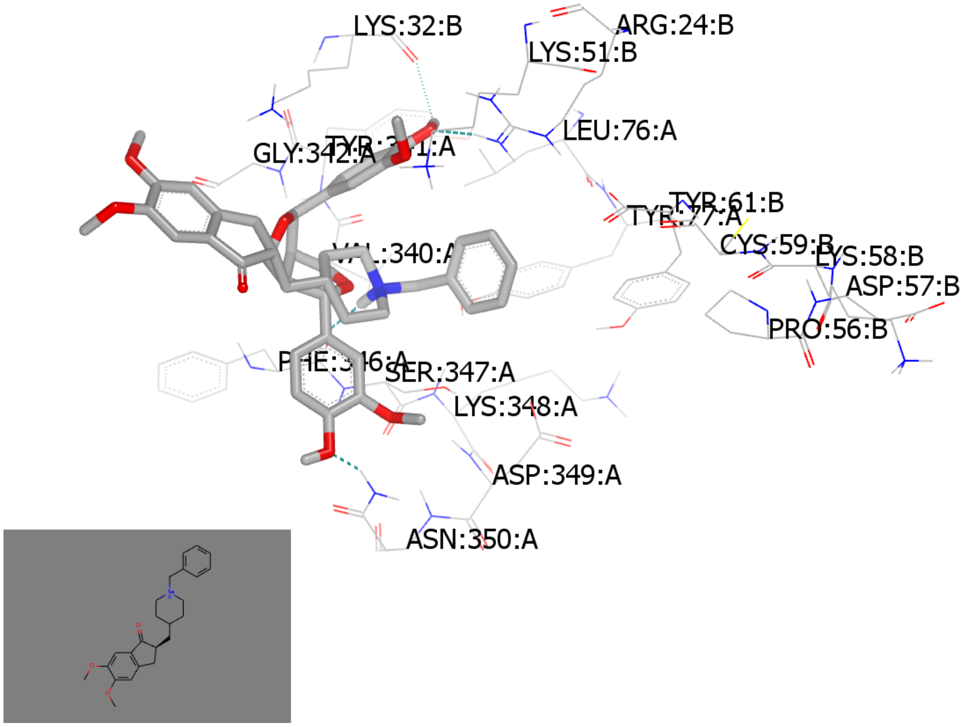

B

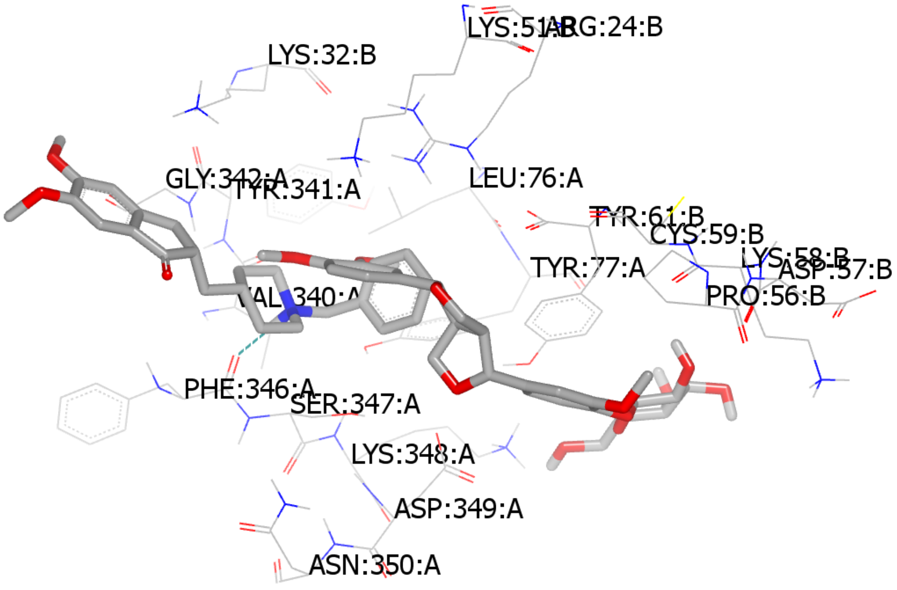

Figure S40: The overlay of donepezil with compounds **3** (A) and **4** (B).

Table S1. ^{1}H and ^{13}C NMR spectral data of compound **1** (CD$_3$OD, 500 and 125 MHz, respectively) and pinoresinol (**2**) ($CDCl_3$, 400 and 100, respectively).

Position	δ_H in ppm, multiplicity (J in Hz)		δ_C in ppm	
	**1**	**2**	**1**	**2**
1			136.1	133.0
2	6.95, d (J = 1.5)	6.87 (d, J = 2.5)	110.9	108.7
3			149.1	146.9
4			147.3	145.4
5	6.77, d (J = 8.0)	6.86 (d, J = 8.1)	116.0	114.4
6	6.92, dd (J = 8.0, 2.0)	6.80 (dd, J = 8.1, 1.8)	119.3	119.1
7	4.74[a], d (J = 4.5)	4.71 (d, J = 4.3)	87.6	86.0
8	3.17[b], m	3.08, m	55.6	54.2
9	4.23[c], ddd (J = 11.5, 7.0, 2.0)<br>3.85* (ov)[d]	4.22 (dd, J = 9.1, 6.9)<br>3.86 (d, J = 3.6)	72.7	71.7
1′	-		133.7	133.0
2′	6.92, brs.	6.87 (d, J = 2.5)	112.1	108.7
3′			145.5	146.9
4′			149.0	145.4
5′		6.86 (d, J = 8.1)	129.9	114.4
6′	6.90, brs.	6.80 (dd, J = 8.1, 1.8)	116.0	119.1
7′	4.72[a], d (J = 4.5)	4.71 (d, J = 4.3)	87.4	86.0
8′	3.15[b], m	3.08, m	55.3	54.2
9′	4.25[c], ddd (J = 11.5, 7.0, 2.0)<br>3.84* (ov)[d]	4.22 (dd, J = 9.1, 6.9)<br>3.84 (d, J = 3.6)	72.5	71.7
1″	-		138.1	
2″	7.03, d (J = 1.6)		111.1	
3″	-		150.9	
4″	-		147.6	
5″	7.14, d (J = 8.5)		118.0	
6″	6.82, dd (J = 8.5, 1.5)		120.0	
7″	5.58, d (J = 5.5)		88.6	
8″	3.48, m		55.6	
9″	3.78, dd (J = 11.0, 7.0)<br>3.85* (ov)[d]		64.9	
1‴	4.89, d (J = 7.5)		102.7	
2‴	3.47, dd (J = 10, 7.5)		74.9	
3‴	3.39, t (J = 10)		78.2	
4‴	3.39, m		71.3	
5‴	3.45, m		77.9	
6‴	3.68, ddd (J = 12.0, 5.5,1.5)<br>3.87* (ov)		62.4	
3′-OCH$_3$	3.85, s	3.88, s	56.4	56.0
3″-OCH$_3$	3.83, s		56.7	
3-OCH$_3$	3.88, s	3.88, s	56.8	56.0

*Multiplicity was not calculated because signals partially overlapped with methoxy signals.

[a,b,c] exchangeable.

(ov)[d] mean overlapped

Table S2. Chemical shift differences of H_2-9 and H_2-9′ (ΔδH-9 and ΔδH-9′) of compounds **1**–**5**.

Compound	$\Delta\delta_{H-9}$	$\Delta\delta_{H-9'}$	Deuterated solvent
**1**	0.38	0.41	CD_3OD
**2**	0.36	0.38	$CDCl_3$
**3**	0.29	0.52	$CDCl_3$
**4**	0.30	0.44	DMSO-d_6
**5**	0.38	0.38	CD_3OD

References

[S1] Tshitenge, D.T.; Feineis, D.; Awale, S. Bringmann, G. Gardenifolins A–H, scalemic neolignans from *Gardenia ternifolia:* Chiral resolution, configurational assignment, and cytotoxic activities against the HeLa cancer cell line. *J. Nat. Prod.* **2017**, *80*, 1604–1614 https://doi.org/10.1021/acs.jnatprod.7b00180

[S2] Tsukamoto, H.; Hisada, S.; Nishibe, S.; Lignans from bark of *Fraxinus mandshurica* var. *japonica* and F. japonica. *Chem. Phar. Bull.* **1984**, *32*, 4482–4489 https://doi.org/10.1248/cpb.32.4482.

[S3] Nishibe, S.; Tsukamoto, H.; Hisada, S. Effects of O-Methylation and O-Glucosylation on Carbon-13 Nuclear Magnetic Resonance Chemical Shifts of Matairesinol, (+)-Pinoresinol and (+)-Epipinoresinol. *Chem. Pharm. Bull.* **1984**, *32*, 4653–4657, https://doi.org/10.1248/CPB.32.4653.

[S4] Rahman, M. M. A., Dewick, P. M., Jackson, D. E., & Lucas, J. A. Lignans of *Forsythia intermedia*. *Phytochemistry*, 1990, *29*, 1971–1980 https://doi.org/10.1016/0031-9422(90)85050-P.

[S5] Dae, K. K.; Jong, P. L.; Jin, W. K.; Hee, W. P.; Jae, S. E. Antitumor and antiinflammatory constituents from *Celtis sinensis*. *Arch. Pharm. Res.* **2005**, *28*, 39–43 https://doi.org/10.1007/BF02975133.

[S6] Gao, L.; Xu, X.; Nan, H.; Yang, J.; Sun, G.; Wu, H.; Zhong, M. Isolation of cinnamic acid derivatives from the root of *Rheum tanguticum* Maxim. ex Balf. And its significance. *J. Med. Plant Res*, **2012**, *6*, 929–931 https://doi.org/10.5897/JMPR11.1497

[S7] El-Sawy, E.; Abo-Salem, H.; Mandour, A. 1H-Indole-3-carboxaldehyde: synthesis and reactions. *Egypt. J. Chem.* **2017,** *60*, 723–751 https://doi.org/10.21608/ejchem.2017.1097.1053.

[S8] Gutierrez-Lugo, M. T.; Woldemichael, G. M.; Singh, M. P.; Suarez, P. A.; Maiese, W. M.; Montenegro, G.; Timmermann, B. N.; Isolation of three new naturally occurring compounds from the culture of *Micromonospora sp*. P1068. *Nat. Prod. Res*. 2006, 19, 645–652 https://doi.org/10.1080/14786410412331272040
